# Comparison of Clinical Trial Changes in Primary Outcome and Reported Intervention Effect Size Between Trial Registration and Publication

**DOI:** 10.1001/jamanetworkopen.2019.7242

**Published:** 2019-07-19

**Authors:** Tao Chen, Chao Li, Rui Qin, Yang Wang, Dahai Yu, James Dodd, Duolao Wang, Victoria Cornelius

**Affiliations:** 1 Department of Epidemiology and Health Statistics, School of Public Health, Xi’an Jiaotong University Health Science Centre, Xi’an, China; 2 Tropical Clinical Trials Unit, Department of Clinical Sciences, Liverpool School of Tropical Medicine, Liverpool, United Kingdom; 3 Department of Health Education, Jiangsu Province Hospital on Integration of Chinese and Western Medicine, Nanjing, China; 4 Medical Research and Biometrics Centre, Fuwai Hospital, National Centre for Cardiovascular Disease, Peking Union Medical College and Chinese Academy of Medical Sciences, Mentougou District, Beijing, China; 5 Arthritis Research UK Primary Care Centre, Research Institute for Primary Care & Health Sciences, Keele University, Keele, United Kingdom; 6 Imperial Clinical Trials Unit, School of Public Health, Imperial College London, London, United Kingdom

## Abstract

**Importance:**

Primary outcome change could threaten the validity of a clinical trial; however, evidence about the consequences on the reported intervention effect size is unclear.

**Objectives:**

To examine the status of randomized clinical trials whose primary outcome changed between trial registration and publication and to quantify the association of this change with the reported intervention effect size.

**Design, Setting, and Participants:**

In this cross-sectional study on the primary report of randomized clinical trials with clear prospectively registered primary outcomes, PubMed and Embase were searched for articles published between January 1, 2011, and December 31, 2015. The search was conducted in January 2016, identifying randomized clinical trials and the combination of keywords and text words related to *registry*.

**Main Outcomes and Measures:**

Based on the developed approach, trials were classified as having primary outcome change when there was a major discrepancy between the registered and published primary outcomes. Intervention effect was estimated or recalculated using the odds ratio (OR) for each comparison. Each component OR is structured so that an OR is less than 1 if the intervention group has a more favorable result than the control group. The ratio of ORs (ROR), which is the summary OR for trials with primary outcome change divided by those without, and its 95% CI were calculated, with a value less than 1 indicating a larger reported intervention effect size in trials with primary outcome change than those without.

**Results:**

Among 29 749 searched articles (28 810 MEDLINE and 939 Embase), 1488 articles were randomly selected for review. Of 389 trials with clear primary outcomes prospectively described in the registry (416 outcomes reported), 33.4% (130 of 389) of trials had at least 1 primary outcome change. Most (66 of 130) of the changes were either not reporting or omitting the primary outcome. In total, 338 trials (365 outcomes and 487 comparisons) were available for quantitative analysis on the reported intervention effect size bias assessment. Compared with those without primary outcome change, trials with primary outcome change showed a 16% (pooled ROR, 0.84; 95% CI, 0.73-0.96) larger reported intervention effect size. The result persisted after adjustment for potential confounders (ROR, 0.81; 95% CI, 0.71-0.93) and other sensitivity and subgroup analyses.

**Conclusions and Relevance:**

Results of this study suggest that inconsistencies between registered and published primary outcomes of clinical trials are common, and trials with primary outcome change are likely to have a larger intervention effect than those without.

## Key Points

**Question:**

Does primary outcome change between trial registration and publication alter a randomized clinical trial’s reported intervention effect size?

**Findings:**

In this cross-sectional study that included 389 trials, 130 of them had at least 1 primary outcome change between registration and publication. This significantly overestimated the reported intervention effect size by 16% compared with those without primary outcome change.

**Meaning:**

Inconsistencies between registered and published primary outcomes in clinical trials are common, with a larger reported intervention effect size among those with primary outcome change than those without.

## Teaser

This cross-sectional study examines the status of randomized clinical trials (RCTs) whose primary outcome changed between trial registration and publication and quantifies the association of this change with the reported intervention effect size.

## Introduction

Randomized clinical trials (RCTs) have a crucial role in assessing the efficacy and safety of a treatment and in advancing medical knowledge.^1^ Clinical trials of investigational medicinal products have been legally required to be registered before participant enrollment since January 5, 2004; furthermore, to improve transparency of results, the International Committee of Medical Journal Editors member journals have required since January 7, 2005, that for publication clinical trials of any intervention should be preregistered.^2^ Registering a trial is mostly free, and options include ClinicalTrials.gov, EU Clinical Trials Register, and International Standard Randomized Controlled Trial Number (ISRCTN) register. Although the reported information differs between registries, a clearly defined and prespecified primary outcome is an important element.^3,4^ Discrepancies between registered and published outcomes may imply selective outcome reporting based on significant *P* values.^5,6^ This practice could threaten the validity of clinical trials by producing conclusions that may mislead physicians and policy makers.^6,7,8^

Although it is well recognized that registries are important tools to reduce the risk of selective reporting of outcomes, Jones et al^7^ found that consistency between planned and published outcomes varied substantially among 27 eligible studies, with a median consistency proportion of 31% (interquartile range, 17%-45%). Similarly, another study^9^ analyzing all interventional trials registered on ClinicalTrials.gov from 1999 to 2012 showed that 32% of trials had their primary outcomes altered between the listed study start and completion dates. However, previous studies in this area have focused on specific design characteristics (eg, pain or continuous outcomes) and have not attempted to quantify to what extent a primary outcome change will alter the invention size that is being estimated.

The objectives of this study were 2-fold. The first objective was to estimate the proportion of RCTs that had a primary outcome change, without restriction by journals, diseases, or registry entries. A second objective was to quantify the consequences associated with primary outcome change on the reported intervention effect size.

## Methods

This study followed the Strengthening the Reporting of Observational Studies in Epidemiology (STROBE) reporting guideline for cross-sectional studies. The study was not submitted for institutional review board approval because all data are publicly available.

### Search Strategy

In this cross-sectional study, we studied the primary report of registered RCTs published between January 1, 2011, and December 31, 2015. A search was conducted in January 2016 using the suggested filters from the Cochrane Collaboration (MEDLINE via PubMed) and the BMJ Evidence Centre (Embase via Ovid) for identifying RCTs and the combination of keywords and text words related to *registry*. No restrictions by journal, disease, or outcome type (ie, continuous or dichotomous outcome) were applied except that reports had to be in English.

We then randomly selected 5% of the retrieved records from each year. Details of the search strategy and sampling method are listed in eTables 1, 2, and 3 in the Supplement, and our study protocol was registered on PROSPERO.^10^

### Study Selection and Eligibility

Articles were screened for relevance by title and abstract and then by the full text to identify primary trial reports. During this process, one of us (T.C.) excluded duplicate publications, protocol studies and analysis plans, system reviews or meta-analyses, and feasibility, pilot, or phase 1 studies, as well as ancillary studies (eg, subgroup analyses, exploratory analyses, secondary outcome analyses, preliminary results, interim analyses, post hoc analyses, pooled analyses, cost-effectiveness analyses, and mechanism research). Next, for each published trial report, we identified the registration number via published articles or clinical trial registries (ClinicalTrials.gov, ISRCTN register, or country-specific registries). We only included trials that were registered before study completion and gave a clear description of the primary outcome in the registry. Two of us (T.C. and R.Q.) checked the full-text articles in the next 2 processes.

### Data Extraction and Risk of Bias (Quality) Assessment

We assigned a unique identification number to each trial included in this study. Data were extracted using a standardized extraction form. For published articles, we extracted the publication information (eg, author name and year of publication) and study characteristics, including study design (eg, noninferiority, superiority, or equivalence), sample size for each group, randomization method (eg, cluster or individual), and type of outcome (eg, time to event, binary, or continuous). For registered information, we extracted the following information: start and end dates of participant enrollment, registration date, the last amendment date, originally registered primary outcomes, and amended outcomes (if applicable).

The risk of bias (RoB) for each trial was assessed by the RoB tool as recommended by the Cochrane Collaboration. Overall RoB was assessed as low risk (low for all Cochrane Collaboration components), high risk (high for ≥1 Cochrane Collaboration component), or unclear risk (unclear for ≥1 Cochrane Collaboration component). Other RoB items included intent-to-treat (ITT) status, trial centers, and source of funding. We defined and classified these items according to published references as follows: deviation from ITT principle (ie, ITT, modified ITT, or no ITT/unknown),^11^ trial centers (ie, multiple centers or single center),^12^ and source of funding (ie, public funding, cofinanced, for-profit funding, not funded, or not reported) (with not-for-profit funding and not funded considered high risk).^13^

### Outcome of Interest

A major discrepancy was defined if the registered and published primary outcomes were different or were assessed at a different time point. This definition is according to a modified classification by Chan et al.^6^ Specifically, the following were considered major discrepancies: (1) a prespecified primary outcome in the trial registration protocol was subsequently reported as a secondary outcome in the final published article; (2) The published primary outcome was described as a secondary outcome in the registry; (3) The prespecified primary outcomes in the trial registration were either omitted or not reported or labeled from the published article; (4) a new primary outcome was introduced in the published article (eg, an outcome that does not appear at all in the registry but is introduced as primary in the article, or one of the components changed among a composite outcome); and (5) the timing of assessment of the primary outcome in the registered protocol and published article differed.

Inconsistencies were independently identified by 2 of us (T.C. and C.L.), and disagreements were resolved by discussion until consensus (κ coefficient, 0.92; 95% CI, 0.88-0.96). If changes to the registered primary outcome were made, they were further reviewed by another one of us (V.C.), and disagreements were resolved by consensus.

### Statistical Analysis

Categorical variables were described by frequencies and percentages and quantitative variables by medians and ranges. We used the κ coefficient to determine the degree of agreement between reviewers.

To quantify overall the consequences associated with primary outcome change on the reported intervention effect size across different types of outcomes, we assumed relative risk, hazard ratio, and odds ratio (OR) to be the same measure. This strategy has been used in published meta-analyses of observational studies.^14,15^ In our study, we considered ORs to be a common estimate, but heterogeneity by different types of outcomes was explored in subgroup analyses. For continuous outcomes, we converted them to ORs according to the method by Hasselblad and Hedges by multiplying the standardized mean differences and their SEs by 1.81 to calculate the log ORs and the corresponding SEs.^16^

For each comparison, we estimated the OR between the intervention group and the control group. Where necessary, we inverted the effect size so that each comparison was indicated by an OR less than 1 if the intervention group had a more favorable result than the control group.

Because an enrolled trial may contribute 2 or more comparisons due to multiple groups and/or outcomes, we used a linear mixed model with the log ORs of each comparison as the dependent variable, primary outcome change as a fixed effect, and study identification number as a random effect after weighting the inverse variation of the log OR of each comparison. Differences are presented by estimating the ratio of ORs (ROR) after anti–log transformation. The ROR is the summary OR for trials with primary outcome change divided by those without, with a value less than 1 indicating a larger reported intervention effect size in trials with primary outcome change than those without. To test the robustness of our study, we conducted 4 sensitivity analyses. First was a mixed model with adjustments for trial characteristics (ie, deviation from ITT principle, study design, trial centers, type of comparator, randomization method, type of outcome, source of funding, and RoB). Second was a mixed model using primary outcome change based on reviewer 1 assessment only or reviewer 2 assessment only. Third, we repeated the mixed model but excluded trials in turn with (1) different levels of RoB (low risk, high risk, or unclear risk), (2) different type of outcomes (time to event, binary, or continuous), and (3) multiple outcomes and/or multiple groups. Fourth was a mixed model with the inverse variation of the log OR as additional covariates rather than weights.

We also carried out subgroup analyses according to prespecified characteristics. These included deviation from ITT principle, study design, trial centers, randomization method, type of outcome, source of funding, and overall RoB.

All data analyses were performed using statistical software (SAS, version 9.4; SAS Institute Inc). *P* values were 2 tailed, and *P* < .05 was considered statistically significant.

## Results

**Figure 1.**
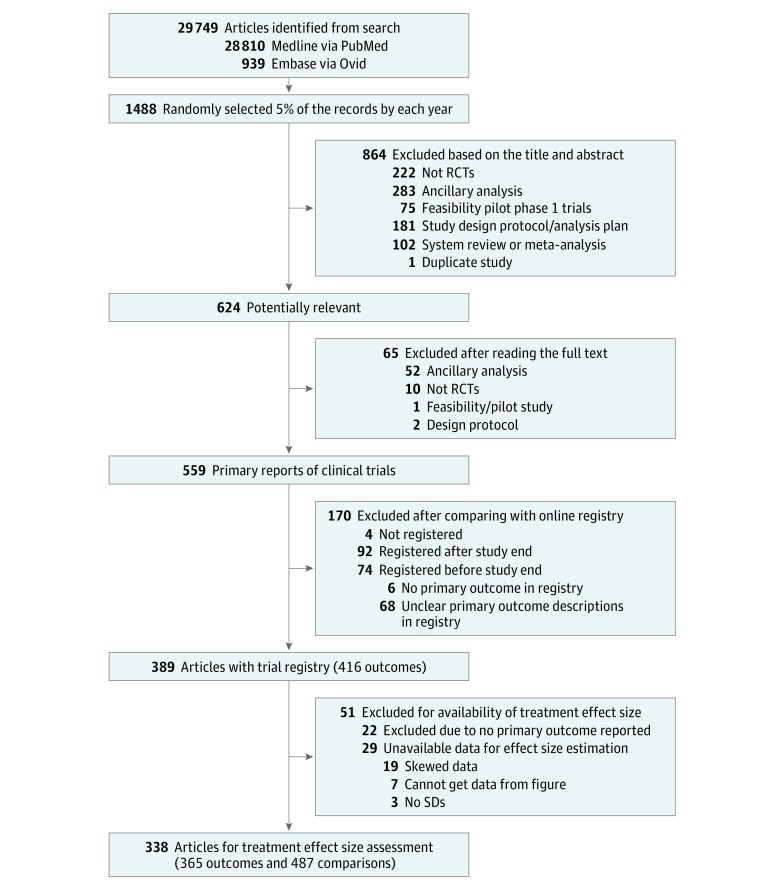
Flowchart of Article Selection RCTs indicates randomized clinical trials; SDs, standard deviations.

Among 29 749 searched articles (28 810 MEDLINE and 939 Embase), 1488 articles were randomly selected for review. After excluding 864 reports by reviewing the titles and abstracts, we identified 624 potentially eligible publications. Of them, 65 were further excluded after reading through the full text. After comparing the remaining 559 publications with online registry, we found that 4 trials (0.7%) were not registered, 92 trials (16.5%) trials were registered after completion of the study, and 74 trials (13.2%) were registered with no description or unclear description of the primary outcome. Figure 1 shows the screening process for both published RCTs and registry records.

### Prevalence of Primary Outcome Change and Its Type

Of 389 trials with clear primary outcomes prospectively described in the registry (416 outcomes reported), 33.4% (130 of 389) of trials had at least 1 primary outcome change. Among those studies with primary outcome change, we found that the most common discrepancy was either omission or not reporting or labeling a registered primary outcome (66 of 130). This was followed by publication of a new outcome (40 of 130), which included 9 composite outcomes with component changes; different timing of assessment in the article and the registry (17 of 130); a registered primary outcome reported as a secondary outcome in the article (6 of 130); and the published primary outcome registered as a secondary outcome (1 of 130). The detailed classification for those trials with primary outcome change and the type of change are listed in eTable 4 in the Supplement.

### Association of Primary Outcome Change With Intervention Effect

To quantify the consequences of the change in primary outcome on the reported intervention effect size, we calculated the OR for each trial. Of the 389 trials, 22 did not report a primary outcome in the publication, and 29 did not have a reproducible way to calculate an OR. As a result, we included 338 trials (365 outcomes and 487 comparisons) for quantitative analysis on the reported intervention effect size bias assessment. The characteristics of trials with and without primary outcome change are listed in Table 1.

**Table 1.** Characteristics of Included Randomized Clinical Trials With and Without Primary Outcome Change With Available Effect Size^a^

Characteristic	With Change (n = 100)	Without Change (n = 238)	*P* Value
Year of publication, No. (%)			
2011-2012	18 (18.0)	42 (17.6)	.93
2012-2013	20 (20.0)	49 (20.6)	
2013-2014	23 (23.0)	57 (23.9)	
2014-2015	27 (27.0)	55 (23.1)	
2015-2016	12 (12.0)	35 (14.7)	
ITT status, No. (%)			
ITT	44 (44.0)	104 (43.7)	.02
mITT	26 (26.0)	91 (38.2)	
No ITT/unknown	30 (30.0)	43 (18.1)	
Study design, No. (%)			
Noninferiority	5 (5.0)	26 (10.9)	.09
Superiority	95 (95.0)	212 (89.1)	
Use of placebo, No. (%)			
Yes	29 (29.0)	83 (34.9)	.30
No	71 (71.0)	155 (65.1)	
Sample size calculation, No. (%)			
Not reported	5 (5.0)	14 (5.9)	.75
Reported	95 (95.0)	224 (94.1)	
Trial centers, No. (%)			
Multiple centers	71 (71.0)	185 (77.7)	.19
Single center	29 (29.0)	53 (22.3)	
Randomization method, No. (%)			
Cluster	9 (9.0)	8 (3.4)	.03
Individual	91 (91.0)	230 (96.6)	
Comparison, No. (%)			
2 Groups and single outcome	66 (66.0)	183 (76.9)	.05
2 Groups but multiple outcomes	3 (3.0)	12 (5.0)	
Multiple groups and single outcome	28 (28.0)	36 (15.1)	
Multiple groups and multiple outcomes	3 (3.0)	7 (2.9)	
No. of outcomes, median (range)	1 (1-3)	1 (1-3)	.96
No. of comparisons, median (range)	1 (1-8)	1 (1-10)	.12
Sequence generation, No. (%)			
Low risk	74 (74.0)	155 (65.1)	.11
Unclear risk	26 (26.0)	83 (34.9)	
Allocation concealment, No. (%)			
Low risk	62 (62.0)	128 (53.8)	.03
High risk	2 (2.0)	0	
Unclear risk	36 (36.0)	110 (46.2)	
Masking of patients and personnel, No. (%)			
Low risk	73 (73.0)	186 (78.2)	.15
High risk	17 (17.0)	23 (9.7)	
Unclear risk	10 (10.0)	29 (12.2)	
Masking of outcome assessor, No./total No. (%)^b^			
Low risk	92/108 (85.2)	208/257 (80.9)	.26
High risk	9/108 (8.3)	13/257 (5.1)	
Unclear risk	7/108 (6.5)	36/257 (14.0)	
Incomplete outcome data, No./total No. (%)^b^			
Low risk	65/108 (60.2)	179/257 (69.6)	.08
High risk	12/108 (11.1)	29/257 (11.3)	
Unclear risk	31/108 (28.7)	49/257 (19.1)	
Type of outcome, No./total No. (%)^b^			
Time to event	11/108 (10.2)	54/257 (21.0)	.94
Binary	51/108 (47.2)	107/257 (41.6)	
Continuous	46/108 (42.6)	96/257 (37.4)	
Source of funding, No. (%)			
Public funding	49 (49.0)	83 (34.9)	.03
Cofinanced	17 (17.0)	35 (14.7)	
For-profit funding	26 (26.0)	106 (44.5)	
Not funded	1 (1.0)	2 (0.8)	
Not reported	7 (7.0)	12 (5.0)	
Overall risk of bias, No. (%)			
Low risk	33 (33.0)	68 (28.6)	.08
High risk	27 (27.0)	45 (18.9)	
Unclear risk	40 (40.0)	125 (52.5)	
Odds ratio, median (range)^c^	0.57 (0.00-2.25)	0.79 (0.01-5.54)	.01

Abbreviations: ITT, intent to treat; mITT, modified intent to treat.

^a^ Studies with no primary outcome and/or recalculable data for treatment effect estimation are excluded.

^b^ Numbers and percentages are based on number of outcomes (n = 365).

^c^ Based on number of comparisons (n = 487).

On average, the reported intervention effect size in trials with primary outcome change was found to be larger by 16% (pooled ROR, 0.84; 95% CI, 0.73-0.96) compared with those without change. This result persisted after adjustment for potential confounders (ROR, 0.81; 95% CI, 0.71-0.93) and using the classification of the primary outcome change from reviewer 1 (unadjusted ROR, 0.83; 95% CI, 0.73-0.95) and reviewer 2 (unadjusted ROR, 0.85; 95% CI, 0.75-0.97). Similarly, we did not find material changes in other sensitivity analyses, with RORs ranging from 0.73 after regarding the study weight as additional covariates rather than the weights in the mixed model to 0.96 after excluding studies with multiple groups (Table 2).

**Table 2.** Main and Sensitivity Analyses With ROR Between Randomized Clinical Trials With and Without Primary Outcome Change^a^

Analysis	No. of Trials	No. of Comparisons	ROR (95% CI)	*P* Value
**Main Analysis**				
Unadjusted	338	487	0.84 (0.73-0.96)	.01
**Sensitivity Analysis**				
Adjusted^b^	338	487	0.81 (0.71-0.93)	.002
Primary outcome change based on reviewer 1 assessment only	338	487	0.83 (0.73-0.95)	.007
Primary outcome change based on reviewer 2 assessment only	338	487	0.85 (0.75-0.97)	.02
Adjusted^c^	338	487	0.73 (0.60-0.89)	.002
Exclusion of studies with time-to-event outcome	279	409	0.81 (0.69-0.95)	.01
Exclusion of studies with binary outcomes	194	275	0.78 (0.65-0.93)	.007
Exclusion of studies with continuous outcomes	209	290	0.88 (0.74-1.05)	.16
Exclusion of low risk	238	348	0.77 (0.65-0.92)	.005
Exclusion of high risk	266	393	0.81 (0.69-0.95)	.008
Exclusion of unclear risk	173	233	0.95 (0.81-1.11)	.59
Exclusion of multiple outcome	313	411	0.80 (0.70-0.92)	.003
Exclusion of multiple groups	264	280	0.96 (0.83-1.11)	.55
Exclusion of multiple outcome or multiple groups	249	250	0.94 (0.82-1.07)	.36

Abbreviation: ROR, ratio of odds ratios.

^a^ Analyses were based on 338 studies with available effect size.

^b^ Based on weighted mixed model with covariates of deviation from intent-to-treat principle, study design, trial centers, type of comparator, randomization method, type of outcome, source of funding, and overall risk of bias.

^c^ Based on mixed model with the same covariates as in footnote b, plus inverse of variance.

### Subgroup Analyses

**Figure 2.**
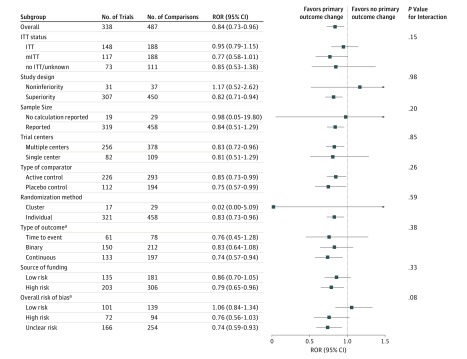
Subgroup Analyses by Various Study Characteristics ITT indicates intent to treat; mITT, modified intent to treat; and ROR, ratio of odds ratios. ^a^Different outcomes could be observed within the same trial.

For multicenter trials, the ROR between changed and unchanged primary outcomes was 0.83 (95% CI, 0.72-0.96). For studies assessing continuous outcomes, the corresponding result was 0.74 (95% CI, 0.57-0.94). For trials using a superiority study design, the ROR between changed and unchanged primary outcomes was 0.82 (95% CI, 0.71-0.94). Overestimation of the reported intervention effect size among trials with primary outcome change could be observed among other subgroups, although they did not all reach statistical significance. In addition, there was no evidence of an interaction between different trial characteristics (eg, study design, multiple centers or single center, and type of outcome) and the estimated intervention effects (Figure 2).

## Discussion

Our cross-sectional study was a survey of contemporary trials that included a broad range of medical conditions and interventions. We found that 33.4% of the sample had at least 1 primary outcome inconsistency between registration and publication. Among studies for which we could calculate an intervention effect, we demonstrated that trials with primary outcome change reported larger intervention effect sizes. This finding remained even after adjustment for RoB items and other potential bias (eg, deviation from ITT principle, multiple centers, and source of funding) and a series of sensitivity analyses (eg, exclusion of studies with binary outcomes). Because we were unable to include trials that either did not declare a primary outcome in the registry or did not register their protocol, this study may underestimate the true consequences of the practice of primary outcome change.

Several studies have assessed the discrepancy rates between registered and published clinical trial outcomes among specific clinical areas (eg, pain),^17,18,19,20,21,22^ journals (eg, general medical journals and high-impact journals),^5,23,24,25^ or registry entries (eg, ClinicalTrials.gov).^5,9,21,26^ A systematic review of studies up to 2014 that compared registered with reported primary outcomes demonstrated a median 31% rate of discrepancies.^7^ We found a similar rate in our study (33.4%), although it was lower than the 60% in recent study^27^ among 192 trials. These findings highlight this prevalent issue after publication of the Consolidated Standards of Reporting Trials (CONSORT) 2010 Statement.^28^ Notably, our analysis found that the 2 most common discrepancies were omission of registered primary outcomes and inclusion of new unregistered outcomes, which have been repeatedly reported in other studies.^5,21,22,24,29,30,31^ Unlike other studies that compared published outcomes with those in the registry at the time of the search^18,22,25,32,33^ or used a specific function (eg, “History of Changes” in the ClinicalTrials.gov archive site),^9^ our study attempted to identify all outcome changes after the initial registration and excluded those registered after the end of the trial. This is because comparison with the original registered outcome is more relevant in understanding the true consequences of outcome switching on the validity of a clinical trial. However, we still observed high rates of trial registration after study completion (92 of 559) and no primary outcome or unclear primary outcome in the registry (74 of 559) in this survey of contemporary trials after the CONSORT 2010 Statement. Such poor quality of trial registration has been highlighted in previous studies.^24,27,32,34,35,36^ Although due to our study design we were unable to address the reasons for primary outcome change, some possibilities need to be assessed in future studies, such as pressure to publish positive results with public funding or high rates of nonpublication among industry-sponsored trials with primary outcome change.

Our study is the first to date to quantify the consequences of primary outcome change on the reported intervention effect size in individual RCTs. Some specific characteristics of a trial, such as deviation from ITT principle,^11,37^ small sample size,^38,39^ concealment of allocation,^40,41^ and single center,^12^ have been assessed in several meta-epidemiological studies. In general, various components of inadequate trial methods are associated with imprecision in the estimated intervention effects, but the magnitude and direction of the bias may vary depending on the medical conditions examined, the definition of inadequate methods, and analytic methods.^11,12,37,38,39,40,41^ In our study, we found that trials with discrepancies between registry and publication show more beneficial treatments than those without. Our results were robust to a series of supplementary adjusted analyses to adjust for potential confounding factors that may contribute to statistical precision in RCTs, as well as to sensitivity analyses to account for the classification of primary outcome change.

Our study is based on a large sample of individual RCTs rather than a meta-epidemiological approach. The study was performed across a range of medical disciplines, registries, and types of outcomes. Therefore, the trials included in our study are likely representative of a cross-section of the general population, and we believe that our results are generalizable to multiple settings. To explore the association of primary outcome change with the reported intervention effect size, we used several analytic approaches, which gave consistent results.

### Limitations

Some caveats should be recognized in our study. First, similar to other meta-epidemiological studies, our study mainly used published information and compared it with records in registries or protocols if necessary. Consequently, our results largely depended on the quality of reporting, which is often unsatisfactory.^42^ Second, almost 40% (221 of 559) of trials could not be assessed due to unavailable or insufficient information on primary outcomes from both articles and registries (n = 170) and unavailability of treatment effect size estimates (n = 51). Together with the reported publication bias (eg, trials sponsored by industry are less likely to be published), this probably led to an underestimation of the proportion of trials with primary outcome change and their association with the reported intervention effect size. Third, although the potential for selective reporting of primary outcomes was discernible in published trials, we first extracted the adjusted treatment differences and their SEs and then the unadjusted ones if there was no clear statement to specify which was the primary analysis result. This is because few trials released their original protocol and statistical analysis plan.

## Conclusions

Results of this study suggest that primary outcome change in RCTs is common and likely overestimates intervention effects. Trial sponsors and investigators should register the primary outcomes, justify changes (if they occur), and report the results accordingly. This will allow the reader to critically appraise and interpret the trial results without bias. Reviewers and editors should routinely use prospectively registered data to avoid changes in primary outcomes during peer review, a practice that has been adopted by leading journals (eg, *JAMA* and *BMJ*). Readers and clinicians must be cautious about interpreting trial results and should be aware that trials with primary outcome change could lead to an overestimation of intervention effects.

## Notes

Supplement. **eTable 1.** Search History in PubMed **eTable 2.** Search History in Embase via Ovid After Excluding Medline Journals **eTable 3.** Number of Hits by Year **eTable 4.** List of Included Randomized Clinical Trials With Classification and Type of Primary Outcome Change **eReferences**
