# Supplementary material for: Comparison of Clinical Trial Changes in Primary Outcome and Reported Intervention Effect Size Between Trial Registration and Publication
Source: JAMA Netw Open. 2019 Jul 19;2(7):e197242. doi: 10.1001/jamanetworkopen.2019.7242 (PMC6646984; doi:10.1001/jamanetworkopen.2019.7242)
Supplement: Supplement. — eTable 1. Search History in PubMed eTable 2. Search History in Embase via Ovid After Excluding Medline Journals eTable 3. Number of Hits by Year eTable 4. List of Included Randomized Clinical Trials With Classification and Type of Primary Outcome Change eReferences [file jamanetwopen-2-e197242-s001.pdf]

Supplementary Online Content

Chen T, Li C, Qin R, et al. Comparison of clinical trial changes in primary outcome and reported intervention effect size between trial registration and publication. *JAMA Netw Open*. 2019;2(7):e197242. doi:10.1001/jamanetworkopen.2019.7242

eTable 1. Search History in PubMed

eTable 2. Search History in EMBASE via Ovid After Excluding Medline Journals

eTable 3. Number of Hits by Year

eTable 4. List of Included Randomized Clinical Trials With Classification and Type of Primary Outcome Change

eReferences.

This supplementary material has been provided by the authors to give readers additional information about their work.

1. Electronic search strategies

We performed electronic search at both PubMed and EMBASE on 23 Jan 2016. The search history is listed in eTable 1 and eTable 2.

eTable 1. Search History in PubMed

Search	Query	Items found	Time
#3	Search (((((((Registries/standards [MESH Terms]) OR registration) OR registry) OR Clinicaltrials.gov) OR ICTRP) OR ISRCTN)) AND (((((((((randomized controlled trial [pt]) OR controlled clinical trial [pt]) OR randomized [tiab]) OR placebo [tiab]) OR clinical trials as topic [mesh: noexp]) OR randomly [tiab]) OR trial [ti])) NOT ((animals [mh] NOT humans [mh]))) Filters: Publication date from 2011/01/01 to 2015/12/31; English	28810	15:43:18
#2	Search ((((((((randomized controlled trial [pt]) OR controlled clinical trial [pt]) OR randomized [tiab]) OR placebo [tiab]) OR clinical trials as topic [mesh: noexp]) OR randomly [tiab]) OR trial [ti])) NOT ((animals [mh] NOT humans [mh]))	930151	15:42:21
#1	Search (((((Registries/standards [MESH Terms]) OR registration) OR registry) OR Clinicaltrials.gov) OR ICTRP) OR ISRCTN	197732	15:40:43

© 2019 Chen T et al. *JAMA Network Open*.

eTable 2. Search History in EMBASE via Ovid After Excluding Medline Journals

Search	Query	Results
1	(random$ or placebo$ or single blind$ or double blind$ or triple blind$).ti,ab.	1160063
2	(animal$ not human$).sh,hw.	3970214
3	(book or conference paper or editorial or letter or review).pt. not exp randomized controlled trial/	4295441
4	(random sampl$ or random digit$ or random effect$ or random survey or random regression).ti,ab. not exp randomized controlled trial/	68833
5	1 not (2 or 3 or 4)	892573
6	(registry or clinicaltrials gov or ictrp or isrctn or registration).tw.	166894
7	5 and 6	22816
8	limit 7 to (english language and exclude medline journals and yr="2011 - 2015")	939

© 2019 Chen T et al. *JAMA Network Open*.

2. Method

In total, there are 29,749 hits (see below).

eTable 3. Number of Hits by Year

Year	Number of hits
2011	5088
2012	5998
2013	7104
2014	7784
2015	3775
Overall	29749

Each item was assigned a number according to the order of appearance in PubMed/Embase by year. A computer-generated random sequence was then created within each year, and then ranked.

Since no similar study had been performed before and there was no standard way to calculate the sample size, we reviewed the sample size for previous studies regarding the association between study quality and treatment effect size and found that most of them included 150 to 310 studies.[1,2 3,4] Therefore, if the first 5% items selected included more than 300 eligible papers, we would not select another 5%.

3. Article listing

eTable 4. List of Included Randomized Clinical Trials With Classification and Type of Primary Outcome Change

© 2019 Chen T et al. *JAMA Network Open*.

First author	year of publication	Journal	Title	Classification	type of changes
F. Cognetti[5]	2013	Gynecol Oncol	A Phase II, randomized, double-blind study of zibotentan (ZD4054) in combination with carboplatin/paclitaxel versus placebo in combination with carboplatin/paclitaxel in patients with advanced ovarian cancer sensitive to platinum-based chemotherapy (AGO-OVAR 2.14).	no	
Arianne C. Lim[6]	2011	Obstet Gynecol	17Î±-hydroxyprogesterone caproate for the prevention of adverse neonatal outcome in multiple pregnancies: a randomized controlled trial.	no	
Christoph C Lees[7]	2015	Lancet	2 year neurodevelopmental and intermediate perinatal outcomes in infants with very preterm fetal growth restriction (TRUFFLE): a randomised trial.	yes	new outcome
Tiia Ngandu[8]	2015	Lancet	A 2 year multidomain intervention of diet, exercise, cognitive training, and vascular risk monitoring versus control to prevent cognitive decline in at-risk elderly people (FINGER): a randomised controlled trial.	yes	omission
Jinhyun Kim[9]	2013	J Korean Med Sci	A clinical trial and extension study of infliximab in Korean patients with active rheumatoid arthritis despite methotrexate treatment.	no	
Brett E. Skolnick[10]	2014	N Engl J Med	A clinical trial of progesterone for severe traumatic brain injury.	no	
Thomas C. Keyserling[11]	2014	JAMA Intern Med	A comparison of live counseling with a web-based lifestyle and medication intervention to reduce coronary heart disease risk: a randomized clinical trial.	no	
Jens Cosedis Nielsen[12]	2011	Eur Heart J	A comparison of single-lead atrial pacing with dual-chamber pacing in sick sinus syndrome.	no	
Juan-Osvaldo Talavera[13]	2013	Curr Med Res Opin	A double-blind, double-dummy, randomized, placebo-controlled trial to evaluate the effect of statin therapy on triglyceride levels in Mexican hypertriglyceridemic patients.	no	

© 2019 Chen T et al. *JAMA Network Open*.

Anthony Yuen Bun Teoh[14]	2012	Ann Surg	A double-blinded randomized controlled trial of laparoendoscopic single-site access versus conventional 3-port appendectomy.	no	
Marta Ramon-Krauel[15]	2013	Child Obes	A low-glycemic-load versus low-fat diet in the treatment of fatty liver in obese children.	no	
Hanik K.Yoo[16]	2013	J Clin Psychiatry	A multicenter, randomized, double-blind, placebo-controlled study of aripiprazole in children and adolescents with Tourette's disorder.	no	
Pierre Quartier[17]	2011	Ann Rheum Dis	A multicentre, randomised, double-blind, placebo-controlled trial with the interleukin-1 receptor antagonist anakinra in patients with systemic-onset juvenile idiopathic arthritis (ANAJIS trial).	yes	new outcome
Dennis G. Maki[18]	2011	Crit Care Med	A novel antimicrobial and antithrombotic lock solution for hemodialysis catheters: a multi-center, controlled, randomized trial.	no	
Andrew C. Chen[19]	2015	N Engl J Med	A Phase 3 Randomized Trial of Nicotinamide for Skin-Cancer Chemoprevention.	yes	new outcome
Rachelle S. Doody[20]	2013	N Engl J Med	A phase 3 trial of semagacestat for treatment of Alzheimer's disease.	no	
Kiran Nanchahal[21]	2012	BMJ Open	A pragmatic randomised controlled trial in primary care of the Camden Weight Loss (CAMWEL) programme.	yes	omission
Michael G. Sarr[22]	2014	Surgery	A prospective, randomized, multicenter trial of Surgisis Gold, a biologic prosthetic, as a sublay reinforcement of the fascial closure after open bariatric surgery.	no	
Ranjit Lall[23]	2015	Health Technol Assess	A randomised controlled trial and cost-effectiveness analysis of high-frequency oscillatory ventilation against conventional artificial ventilation for adults with acute respiratory distress syndrome. The OSCAR (OSCillation in ARDS) study.	no	

© 2019 Chen T et al. *JAMA Network Open*.

Lesley M Roberts[24]	2013	BMC Gastroenterol	A randomised controlled trial of a probiotic 'functional food' in the management of irritable bowel syndrome.	no	
Ramachandran Venkitaraman[25]	2015	Eur Urol	A randomised phase 2 trial of dexamethasone versus prednisolone in castration-resistant prostate cancer.	no	
Bruce E Sands[26]	2013	Gut	A randomised, double-blind, sham-controlled study of granulocyte/monocyte apheresis for moderate to severe Crohn's disease.	yes	new outcome
Thomas Yeoh[27]	2011	Heart Lung Circ	A randomised, placebo-controlled trial of carvedilol in early familial dilated cardiomyopathy.	yes	omission
Hawk Kim[28]	2012	Ann Hematol	A randomized comparison of cyclophosphamide vs. reduced dose cyclophosphamide plus fludarabine for allogeneic hematopoietic cell transplantation in patients with aplastic anemia and hypoplastic myelodysplastic syndrome.	yes	new outcome
Neil stollman[29]	2013	J Clin Gastroenterol	A randomized controlled study of mesalamine after acute diverticulitis: results of the DIVA trial.	yes	time changes
Angelo E. Volandes[30]	2012	J Palliat Med	A randomized controlled trial of a goals-of-care video for elderly patients admitted to skilled nursing facilities.	no	
Mark D Farrar[31]	2015	Am J Clin Nutr	A randomized controlled trial of green tea catechins in protection against ultraviolet radiation-induced cutaneous inflammation.	no	
Kieran J. Phelan[32]	2011	Arch Pediatr Adolesc Med	A randomized controlled trial of home injury hazard reduction: the HOME injury study.	yes	new outcome
Sagar V.Parkh[33]	2012	J Clin Psychiatry	A randomized controlled trial of psychoeducation or cognitive-behavioral therapy in bipolar disorder: a Canadian Network for Mood and Anxiety treatments (CANMAT) study [CME].	yes	omission

© 2019 Chen T et al. *JAMA Network Open*.

Paul Y. Takahashi[34]	2012	Arch Intern Med	A randomized controlled trial of telemonitoring in older adults with multiple health issues to prevent hospitalizations and emergency department visits.	yes	omission
Corrine I. Voils[35]	2013	Prev Med	A randomized controlled trial to evaluate the effectiveness of CouPLES: a spouse-assisted lifestyle change intervention to improve low-density lipoprotein cholesterol.	no	
Myriam Harfterkamp[36]	2012	J Am Acad Child Adolesc Psychiatry	A randomized double-blind study of atomoxetine versus placebo for attention-deficit/hyperactivity disorder symptoms in children with autism spectrum disorder.	no	
C. H. Smorenburg[37]	2014	Ann Oncol	A randomized phase III study comparing pegylated liposomal doxorubicin with capecitabine as first-line chemotherapy in elderly patients with metastatic breast cancer: results of the OMEGA study of the Dutch Breast Cancer Research Group BOOG.	no	
George Van Buren II[38]	2014	Ann Surg	A randomized prospective multicenter trial of pancreaticoduodenectomy with and without routine intraperitoneal drainage.	no	
Angelo Naselli[39]	2012	European Urology, Supplements	A randomized prospective trial to assess impact of TUR performed in narrow band imaging modality on non muscle invasive bladder cancer recurrence.	no	
Charles W. Lynde[40]	2012	J Dermatolog Treat	A randomized study comparing the combination of nbUVB and etanercept to etanercept monotherapy in patients with psoriasis who do not exhibit an excellent response after 12 weeks of etanercept.	no	
Andrea O. Rossetti[41]	2011	Neurocrit Care	A randomized trial for the treatment of refractory status epilepticus.	no	
Massimo Imazio[42]	2013	N Engl J Med	A randomized trial of colchicine for acute pericarditis.	yes	new outcome
Tina M. Slusher[43]	2015	N Engl J Med	A Randomized Trial of Phototherapy with Filtered Sunlight in African Neonates.	yes	new outcome

© 2019 Chen T et al. *JAMA Network Open*.

N. Low, A. Bavdekar[44]	2015	N Engl J Med	A randomized, controlled trial of an aerosolized vaccine against measles.	no	
David A. Berntsen[45]	2012	Invest Ophthalmol Vis Sci	A randomized trial using progressive addition lenses to evaluate theories of myopia progression in children with a high lag of accommodation.	no	
Hideaki Katagiri[46]	2013	BMC Psychiatry	A randomized, double-blind, placebo-controlled study of rapid-acting intramuscular olanzapine in Japanese patients for schizophrenia with acute agitation.	no	
J. L. C. Borges[47]	2011	Diabetes Obes Metab	A randomized, parallel group, double-blind, multicentre study comparing the efficacy and safety of Avandamet (rosiglitazone/metformin) and metformin on long-term glycaemic control and bone mineral density after 80 weeks of treatment in drug-naÃ¯ve type 2 diabetes mellitus patients.	no	
Katy E. Mitchell[48]	2014	Eur Respir J	A self-management programme for COPD: a randomised controlled trial.	no	
J. Tan[49]	2014	Br J Dermatol	A treatment for severe nodular acne: a randomized investigator-blinded, controlled, noninferiority trial comparing fixed-dose adapalene/benzoyl peroxide plus doxycycline vs. oral isotretinoin.	no	
Philip Mease[50]	2011	Arthritis Rheum	Abatacept in the treatment of patients with psoriatic arthritis: results of a six-month, multicenter, randomized, double-blind, placebo-controlled, phase II trial.	yes	new times
Kamran Siddiqi[51]	2013	Ann Intern Med	Action to stop smoking in suspected tuberculosis (ASSIST) in Pakistan: a cluster randomized, controlled trial.	yes	omission
Willem Pieter Brouwer[52]	2015	Hepatology	Adding pegylated interferon to entecavir for hepatitis B e antigen-positive chronic hepatitis B: A multicenter randomized trial (ARES study).	no	
Lotte M. Kruidenier[53]	2011	J Vasc Interv Radiol	Additional supervised exercise therapy after a percutaneous vascular intervention for peripheral arterial disease: a randomized clinical trial.	yes	time changes

© 2019 Chen T et al. *JAMA Network Open*.

Johan Vansteenkiste[54]	2013	J Clin Oncol	Adjuvant MAGE-A3 immunotherapy in resected non-small-cell lung cancer: phase II randomized study results.	yes	new outcome
Jordi Bruix[55]	2015	Lancet Oncol	Adjuvant sorafenib for hepatocellular carcinoma after resection or ablation (STORM): a phase 3, randomised, double-blind, placebo-controlled trial.	no	
JM Watson[56]	2013	Health Technol Assess	AESOPS: a randomised controlled trial of the clinical effectiveness and cost-effectiveness of opportunistic screening and stepped care interventions for older hazardous alcohol users in primary care.	no	
Graziella Badin Aliti[57]	2013	JAMA Intern Med	Aggressive fluid and sodium restriction in acute decompensated heart failure: a randomized clinical trial.	yes	time changes
P Higgins[58]	2014	Heart	Allopurinol reduces brachial and central blood pressure, and carotid intima-media thickness progression after ischaemic stroke and transient ischaemic attack: a randomised controlled trial.	no	
C. Parker[59]	2013	N Engl J Med	Alpha emitter radium-223 and survival in metastatic prostate cancer.	no	
John J.V. McMurray[60]	2014	N Engl J Med	Angiotensin-neprilysin inhibition versus enalapril in heart failure.	no	
Stuart J. Connolly[61]	2011	N Engl J Med	Apixaban in patients with atrial fibrillation.	no	
Richard K Burt[62]	2011	Lancet	Autologous non-myeloablative haemopoietic stem-cell transplantation compared with pulse cyclophosphamide once per month for systemic sclerosis (ASSIST): an open-label, randomised phase 2 trial.	yes	omission
Takahiko Kojima[63]	2011	Circ J	Azelnidipine and amlodipine anti-coronary atherosclerosis trial in hypertensive patients undergoing coronary intervention by serial volumetric intravascular ultrasound analysis in Juntendo University (ALPS-J).	no	

© 2019 Chen T et al. *JAMA Network Open*.

Richard K. Albert[64]	2011	N Engl J Med	Azithromycin for prevention of exacerbations of COPD.	no	
Alberto Papi[65]	2013	Lancet Respir Med	Beclometasone-formoterol as maintenance and reliever treatment in patients with asthma: a double-blind, randomised controlled trial.	no	
Nicola A. Hanania[66]	2012	Respir Med	Benefits of adding fluticasone propionate/salmeterol to tiotropium in moderate to severe COPD.	no	
J. E. Foley[67]	2011	Diabetologia	Beta cell function following 1 year vildagliptin or placebo treatment and after 12 week washout in drug-naive patients with type 2 diabetes and mild hyperglycaemia: a randomised controlled trial.	no	
Mabrouk M. Elgadi[68]	2011	Drugs R D	Boosted tipranavir versus darunavir in treatment-experienced patients: observational data from the randomized POTENT trial.	no	
Tamera J. Corte[69]	2014	Am J Respir Crit Care Med	Bosentan in pulmonary hypertension associated with fibrotic idiopathic interstitial pneumonia.	no	
i.c.Lawrance [70]	2011	Endoscopy	Bowel cleansing for colonoscopy: prospective randomized assessment of efficacy and of induced mucosal abnormality with three preparation agents.	yes	new outcome
Robert E. Coleman[71]	2011	N Engl J Med	Breast-cancer adjuvant therapy with zoledronic acid.	no	
Ian H Kunkler[72]	2015	Lancet Oncol	Breast-conserving surgery with or without irradiation in women aged 65 years or older with early breast cancer (PRIME II): a randomised controlled trial.	no	
Nusrat Husain[73]	2014	Br J Psychiatry	Brief psychological intervention after self-harm: randomised controlled trial from Pakistan.	yes	time changes

H. Gelderblom[74]	2014	Eur J Cancer	Brostallicin versus doxorubicin as first-line chemotherapy in patients with advanced or metastatic soft tissue sarcoma: an European Organisation for Research and Treatment of Cancer Soft Tissue and Bone Sarcoma Group randomised phase II and pharmacogenetic study.	no	
Woon Geon Shin[75]	2012	Gastrointest Endosc	Can rebamipide and proton pump inhibitor combination therapy promote the healing of endoscopic submucosal dissection-induced ulcers? A randomized, prospective, multicenter study.	yes	time changes
Jithin Yohannan[76]	2013	JAMA Ophthalmol	Can we stop mass drug administration prior to 3 annual rounds in communities with low prevalence of trachoma?: PRET Ziada trial results.	yes	time changes
Harriette R. Mogul[77]	2014	PLoS One	Carbohydrate modified diet & insulin sensitizers reduce body weight & modulate metabolic syndrome measures in EMPOWIR (enhance the metabolic profile of women with insulin resistance): a randomized trial of normoglycemic women with midlife weight gain.	yes	omission
Elisabeth Quoix[78]	2011	Lancet	Carboplatin and weekly paclitaxel doublet chemotherapy compared with monotherapy in elderly patients with advanced non-small-cell lung cancer: IFCT-0501 randomised, phase 3 trial.	no	
Nicholas A Zwar[79]	2012	Med J Aust	Care of patients with a diagnosis of chronic obstructive pulmonary disease: a cluster randomised controlled trial.	yes	omission
JA Costa [80]	2013	Spinal Cord	Catheter length preference in wheelchair-using men who perform routine clean intermittent catheterization.	no	
Nicholas D James[81]	2012	Lancet Oncol	Celecoxib plus hormone therapy versus hormone therapy alone for hormone-sensitive prostate cancer: first results from the STAMPEDE multiarm, multistage, randomised controlled trial.	no	
I.-J. Wang[82]	2015	Clin Exp Allergy	Children with atopic dermatitis show clinical improvement after Lactobacillus exposure.	yes	omission
Rebecca J Kean[83]	2015	Am J Clin Nutr	Chronic consumption of flavanone-rich orange juice is associated with cognitive benefits: an 8-wk, randomized, double-blind, placebo-controlled trial in healthy older adults.	yes	omission

Paul L.P. Brand[84]	2011	Respir Med	Ciclesonide in wheezy preschool children with a positive asthma predictive index or atopy.	yes	new outcome
Dermot Ryan[85]	2012	BMJ	Clinical and cost effectiveness of mobile phone supported self monitoring of asthma: multicentre randomised controlled trial.	no	
David L. Wessel[86]	2013	N Engl J Med	Clopidogrel in infants with systemic-to-pulmonary-artery shunts.	yes	new outcome
Anne Forster[87]	2015	Stroke	Cluster Randomized Controlled Trial: Clinical and Cost-Effectiveness of a System of Longer-Term Stroke Care.	no	
Thomas K Houston[88]	2013	BMC Oral Health	Cluster-randomized trial of a web-assisted tobacco quality improvement intervention of subsequent patient tobacco product use: a National Dental PBRN study.	yes	secondary to primary
Jeffrey J Wood[89]	2015	Behav Ther	Cognitive behavioral therapy for early adolescents with autism spectrum disorders and clinical anxiety: a randomized, controlled trial.	no	
Josep M Ramon[90]	2014	BMC Med	Combining varenicline and nicotine patches: a randomized controlled trial study in smoking cessation.	no	
Charlotte Gry Harmsen[91]	2014	Br J Gen Pract	Communicating risk using absolute risk reduction or prolongation of life formats: cluster-randomised trial in general practice.	yes	registry to primary but reported as secondary
Brian I Rini[92]	2011	Lancet	Comparative effectiveness of axitinib versus sorafenib in advanced renal cell carcinoma (AXIS): a randomised phase 3 trial.	no	
M. R. OSTOVANEH[93]	2014	Neurogastroenterol Motil	Comparing omeprazole with fluoxetine for treatment of patients with heartburn and normal endoscopy who failed once daily proton pump inhibitors: double-blind placebo-controlled trial.	yes	secondary to primary

© 2019 Chen T et al. *JAMA Network Open*.

Ensieh Shahrokh Tehraninejad[94]	2012	J Assist Reprod Genet	Comparison of cabergoline and intravenous albumin in the prevention of ovarian hyperstimulation syndrome: a randomized clinical trial.	yes	omission
T.J. Hilton[95]	2013	J Dent Res	Comparison of CaOH with MTA for direct pulp capping: a PBRN randomized clinical trial.	no	
Richard P. Allen[96]	2014	N Engl J Med	Comparison of pregabalin with pramipexole for restless legs syndrome.	yes	time changes
Heng Zhang[97]	2015	Arthritis Res Ther	Comparison of two hyaluronic acid formulations for safety and efficacy (CHASE) study in knee osteoarthritis: a multicenter, randomized, double-blind, 26-week non-inferiority trial comparing Durolane to Artz.	no	
Anders Prestmo[98]	2015	Lancet	Comprehensive geriatric care for patients with hip fractures: a prospective, randomised, controlled trial.	yes	omission
Romana Dmitrovic[99]	2012	Obstet Gynecol	Continuous compared with cyclic oral contraceptives for the treatment of primary dysmenorrhea: a randomized controlled trial.	no	
Rony Carlos Preti[100]	2013	Br J Ophthalmol	Contrast sensitivity evaluation in high risk proliferative diabetic retinopathy treated with panretinal photocoagulation associated or not with intravitreal bevacizumab injections: a randomised clinical trial.	no	
Gareth J. Morgan[101]	2011	Blood	Cyclophosphamide, thalidomide, and dexamethasone (CTD) as initial therapy for patients with multiple myeloma unsuitable for autologous transplantation.	no	
Benedikt Huttner[102]	2013	J Antimicrob Chemother	Decolonization of intestinal carriage of extended-spectrum Î²-lactamase-producing Enterobacteriaceae with oral colistin and neomycin: a randomized, double-blind, placebo-controlled trial.	no	
Timothy P. Hughes[103]	2014	Blood	Deep molecular responses achieved in patients with CML-CP who are switched to nilotinib after long-term imatinib.	no	

Selim Corbacioglu[104]	2012	Lancet	Defibrotide for prophylaxis of hepatic veno-occlusive disease in paediatric haemopoietic stem-cell transplantation: an open-label, phase 3, randomised controlled trial.	yes	omission
Chung-Hua Hsu[105]	2011	Altern Med Rev	Does supplementation with green tea extract improve insulin resistance in obese type 2 diabetics? A randomized, double-blind, and placebo-controlled clinical trial.	no	
Carmen A. Prieto-Jimenez[106]	2011	J Pediatr Gastroenterol Nutr	Double-blind randomized trial of quadruple sequential Helicobacter pylori eradication therapy in asymptomatic infected children in El Paso, Texas.	yes	secondary to primary
Irina Mezhebovsky[107]	2013	Int J Geriatr Psychiatry	Double-blind, randomized study of extended release quetiapine fumarate (quetiapine XR) monotherapy in older patients with generalized anxiety disorder.	no	
Ian Judson[108]	2014	Lancet Oncol	Doxorubicin alone versus intensified doxorubicin plus ifosfamide for first-line treatment of advanced or metastatic soft-tissue sarcoma: a randomised controlled phase 3 trial.	no	
Jean-Louis Trouillet[109]	2011	Ann Intern Med	Early percutaneous tracheotomy versus prolonged intubation of mechanically ventilated patients after cardiac surgery: a randomized trial.	no	
Martin L. Blakely[110]	2011	Arch Surg	Early vs interval appendectomy for children with perforated appendicitis.	no	
Robert P. Giugliano[111]	2013	N Engl J Med	Edoxaban versus warfarin in patients with atrial fibrillation.	no	
Kenneth D. Coburn[112]	2012	PLoS Med	Effect of a community-based nursing intervention on mortality in chronically ill older adults: a randomized controlled trial.	no	
Simon J Griffin[113]	2011	Lancet	Effect of early intensive multifactorial therapy on 5-year cardiovascular outcomes in individuals with type 2 diabetes detected by screening (ADDITION-Europe): a cluster-randomised trial.	no	

© 2019 Chen T et al. *JAMA Network Open*.

Rehana A Salam[114]	2015	Arch Dis Child Fetal Neonatal Ed	Effect of emollient therapy on clinical outcomes in preterm neonates in Pakistan: a randomised controlled trial.	no	
Kalathil K. Sureshkumar[115]	2012	Clin J Am Soc Nephrol	Effect of high-dose erythropoietin on graft function after kidney transplantation: a randomized, double-blind clinical trial.	no	
Sophie Boisson[116]	2013	PLoS Med	Effect of household-based drinking water chlorination on diarrhoea among children under five in Orissa, India: a double-blind randomised placebo-controlled trial.	no	
Nele Devoogdt[117]	2011	BMJ	Effect of manual lymph drainage in addition to guidelines and exercise therapy on arm lymphoedema related to breast cancer: randomised controlled trial.	no	
Jean Reignier[118]	2013	JAMA	Effect of not monitoring residual gastric volume on risk of ventilator-associated pneumonia in adults receiving mechanical ventilation and early enteral feeding: a randomized controlled trial.	no	
Marian Wiegersma[119]	2014	BMJ	Effect of pelvic floor muscle training compared with watchful waiting in older women with symptomatic mild pelvic organ prolapse: randomised controlled trial in primary care.	yes	omission
Margaret M. Redfield[120]	2013	JAMA	Effect of phosphodiesterase-5 inhibition on exercise capacity and clinical status in heart failure with preserved ejection fraction: a randomized clinical trial.	no	
Kim L. Bennell[121]	2014	JAMA	Effect of physical therapy on pain and function in patients with hip osteoarthritis: a randomized clinical trial.	yes	omission
Maryam Eftekhar[122]	2013	International Journal of Fertility and Sterility	Effect of progesterone supplementation on natural frozen-thawed embryo transfer cycles: A randomized controlled trial.	yes	secondary to primary
Harsha Thirumurthy[123]	2014	JAMA	Effect of providing conditional economic compensation on uptake of voluntary medical male circumcision in Kenya: a randomized clinical trial.	no	

Sayeed Ikramuddin[124]	2014	JAMA	Effect of reversible intermittent intra-abdominal vagal nerve blockade on morbid obesity: the ReCharge randomized clinical trial.	no	
Miles D. Witham[125]	2013	Atherosclerosis	Effect of short-term vitamin D supplementation on markers of vascular health in South Asian women living in the UK--a randomised controlled trial.	no	
Germar-Michael Pinggera[126]	2014	Urology	Effect of tadalafil once daily on prostate blood flow and perfusion in men with lower urinary tract symptoms secondary to benign prostatic hyperplasia: a randomized, double-blind, multicenter, placebo-controlled trial.	no	
Stephen J. Nicholls[127]	2011	N Engl J Med	Effect of two intensive statin regimens on progression of coronary disease.	no	
Mihai Gheorghiade[128]	2015	JAMA	Effect of Vericiguat, a Soluble Guanylate Cyclase Stimulator, on Natriuretic Peptide Levels in Patients With Worsening Chronic Heart Failure and Reduced Ejection Fraction: The SOCRATES-REDUCED Randomized Trial.	no	
Theresa M. Marteau[129]	2012	PLoS One	Effect on adherence to nicotine replacement therapy of informing smokers their dose is determined by their genotype: a randomised controlled trial.	no	
Brian Yoshio Laing[130]	2014	Ann Intern Med	Effectiveness of a smartphone application for weight loss compared with usual care in overweight primary care patients: a randomized, controlled trial.	no	
Jennifer K. Coffeng[131]	2014	PLoS One	Effectiveness of a worksite social & physical environment intervention on need for recovery, physical activity and relaxation; results of a randomized controlled trial.	yes	omission
Alan N. Barkun[132]	2013	CMAJ	Effectiveness of disseminating consensus management recommendations for ulcer bleeding: a cluster randomized trial.	no	
Philipp Zanger[133]	2013	Lancet Infect Dis	Effectiveness of rifaximin in prevention of diarrhoea in individuals travelling to south and southeast Asia: a randomised, double-blind, placebo-controlled, phase 3 trial.	no	

Ronald I. Shorr[134]	2012	Ann Intern Med	Effects of an intervention to increase bed alarm use to prevent falls in hospitalized patients: a cluster randomized trial.	no	
Beatriz Grinsztejn[135]	2014	Lancet Infect Dis	Effects of early versus delayed initiation of antiretroviral treatment on clinical outcomes of HIV-1 infection: results from the phase 3 HPTN 052 randomised controlled trial.	yes	new outcome
Chris T. Bolliger[136]	2011	Clin Ther	Effects of varenicline in adult smokers: a multinational, 24-week, randomized, double-blind, placebo-controlled study.	no	
Joachim Sieper[137]	2013	Ann Rheum Dis	Efficacy and safety of adalimumab in patients with non-radiographic axial spondyloarthritis: results of a randomised placebo-controlled trial (ABILITY-1).	no	
Jun Ishigooka[138]	2015	Schizophr Res	Efficacy and safety of aripiprazole once-monthly in Asian patients with schizophrenia: a multicenter, randomized, double-blind, non-inferiority study versus oral aripiprazole.	no	
S. Whittaker[139]	2012	Br J Dermatol	Efficacy and safety of bexarotene combined with psoralen-ultraviolet A (PUVA) compared with PUVA treatment alone in stage IB-IIA mycosis fungoides: final results from the EORTC Cutaneous Lymphoma Task Force phase III randomized clinical trial (NCT00056056).	yes	secondary to primary
José A. Vazquez[140]	2012	Curr Med Res Opin	Efficacy and safety of ceftazidime-avibactam versus imipenem-cilastatin in the treatment of complicated urinary tract infections, including acute pyelonephritis, in hospitalized adults: results of a prospective, investigator-blinded, randomized study.	no	
Xavier Girerd[141]	2011	Blood Press Suppl	Efficacy and safety of early versus late titration of fixed-dose irbesartan/hydrochlorothiazide: ACTUAL study.	no	
Ashley Woodcock[142]	2013	Chest	Efficacy and safety of fluticasone furoate/vilanterol compared with fluticasone propionate/salmeterol combination in adult and adolescent patients with persistent asthma: a randomized trial.	no	
J Sieper[143]	2014	Ann Rheum Dis	Efficacy and safety of infliximab plus naproxen versus naproxen alone in patients with early, active axial spondyloarthritis: results from the double-blind, placebo-controlled INFAST study, Part 1.	no	

Roland Buhl[144]	2015	Thorax	Efficacy and safety of once-daily QVA149 compared with the free combination of once-daily tiotropium plus twice-daily formoterol in patients with moderate-to-severe COPD (QUANTIFY): a randomised, non-inferiority study.	yes	new outcome
Vivian Fonseca[145]	2013	J Diabetes Complications	Efficacy and safety of sitagliptin added to ongoing metformin and pioglitazone combination therapy in a randomized, placebo-controlled, 26-week trial in patients with type 2 diabetes.	no	
Johann Altenberger[146]	2014	Eur J Heart Fail	Efficacy and safety of the pulsed infusions of levosimendan in outpatients with advanced heart failure (LevoRep) study: a multicentre randomized trial.	no	
Paul W. Jones[147]	2012	Eur Respir J	Efficacy and safety of twice-daily aclidinium bromide in COPD patients: the ATTAIN study.	yes	omission
B. Charbonnel[148]	2013	Diabetologia	Efficacy and safety over 26 weeks of an oral treatment strategy including sitagliptin compared with an injectable treatment strategy with liraglutide in patients with type 2 diabetes mellitus inadequately controlled on metformin: a randomised clinical trial.	no	
Rainard Fuhr[149]	2012	Chest	Efficacy of aclidinium bromide 400 Î¼g twice daily compared with placebo and tiotropium in patients with moderate to severe COPD.	no	
Taciana Duque Braga[150]	2011	Am J Clin Nutr	Efficacy of Bifidobacterium breve and Lactobacillus casei oral supplementation on necrotizing enterocolitis in very-low-birth-weight preterm infants: a double-blind, randomized, controlled trial	no	
A.S. Papas[151]	2012	J Dent Res	Efficacy of chlorhexidine varnish for the prevention of adult caries: a randomized trial.	no	
P Noel Barrett[152]	2011	Lancet	Efficacy, safety, and immunogenicity of a Vero-cell-culture-derived trivalent influenza vaccine: a multicentre, double-blind, randomised, placebo-controlled trial.	yes	omission
T.M. Beer[153]	2014	N Engl J Med	Enzalutamide in metastatic prostate cancer before chemotherapy.	no	

© 2019 Chen T et al. *JAMA Network Open*.

Philippe Jouve[154]	2013	Anesthesiology	Epidural versus continuous preperitoneal analgesia during fast-track open colorectal surgery: a randomized controlled trial.	no	
W. SONGPRAKUN[155]	2012	J Psychiatr Ment Health Nurs	Evaluation of a cognitive behavioural self-help manual for reducing depression: a randomized controlled trial.	yes	omission
Gaurang R Shah[156]	2012	BMC Complement Altern Med	Evaluation of a multi-herb supplement for erectile dysfunction: a randomized double-blind, placebo-controlled study.	no	
Jeffrey Kaine[157]	2012	Ann Rheum Dis	Evaluation of abatacept administered subcutaneously in adults with active rheumatoid arthritis: impact of withdrawal and reintroduction on immunogenicity, efficacy and safety (phase Iiib ALLOW study).	yes	omission
Eric S. Orwoll[158]	2014	J Clin Invest	Evaluation of teriparatide treatment in adults with osteogenesis imperfecta.	yes	new outcome
Alice D. Domar[159]	2015	Fertil Steril	Exploratory randomized trial on the effect of a brief psychological intervention on emotions, quality of life, discontinuation, and pregnancy rates in in vitro fertilization patients.	no	
D.L. Morton[160]	2014	N Engl J Med	Final trial report of sentinel-node biopsy versus nodal observation in melanoma.	yes	new outcome
Sanne K Poulsen[161]	2014	Am J Clin Nutr	Health effect of the New Nordic Diet in adults with increased waist circumference: a 6-mo randomized controlled trial.	yes	omission
Prabaharan Chellamuthu[162]	2014	Epilepsy Res	High dose (4 mg/kg/day) versus usual dose (2 mg/kg/day) oral prednisolone for treatment of infantile spasms: an open-label, randomized controlled trial.	no	
Pierre Asfar[163]	2014	N Engl J Med	High versus low blood-pressure target in patients with septic shock.	no	

Naoyoshi Nagata[164]	2015	Ann Surg	High-dose barium impaction therapy for the recurrence of colonic diverticular bleeding: a randomized controlled trial.	no	
S. Tiplady[165]	2013	Hum Reprod	Home ovulation tests and stress in women trying to conceive: a randomized controlled trial.	yes	omission
Michael Teut[166]	2013	Trials	Homeopathic drug proving of Okoubaka aubrevillei: a randomised placebo-controlled trial.	no	
Clifton O Bingham III[167]	2015	Ann Rheum Dis	Humoral immune response to vaccines in patients with rheumatoid arthritis treated with tocilizumab: results of a randomised controlled trial (VISARA).	no	
Jörg Hausleiter[168]	2012	JACC Cardiovasc Imaging	Image quality and radiation exposure with prospectively ECG-triggered axial scanning for coronary CT angiography: the multicenter, multivendor, randomized PROTECTION-III study.	no	
Feng-Cai Zhu[169]	2013	Lancet	Immunogenicity and safety of an enterovirus 71 vaccine in healthy Chinese children and infants: a randomised, double-blind, placebo-controlled phase 2 clinical trial.	yes	time changes
Peter Nash[170]	2013	Arthritis Care Res (Hoboken)	Immunogenicity, safety, and efficacy of abatacept administered subcutaneously with or without background methotrexate in patients with rheumatoid arthritis: results from a phase III, international, multicenter, parallel-arm, open-label study.	no	
Howman, Andrew [171]	2013	Lancet	Immunosuppression for progressive membranous nephropathy: a UK randomised controlled trial.	no	
Romain Dugravier[172]	2013	PLoS One	Impact of a manualized multifocal perinatal home-visiting program using psychologists on postnatal depression: the CAPEDP randomized controlled trial.	yes	omission
Annie Y S Lau[173]	2012	J Am Med Inform Assoc	Impact of a web-based personally controlled health management system on influenza vaccination and health services utilization rates: a randomized controlled trial.	no	

© 2019 Chen T et al. *JAMA Network Open*.

Stephen M. Cohn[174]	2011	World J Surg	Impact of low-dose vasopressin on trauma outcome: prospective randomized study.	yes	omission
Swagata Ganguly[175]	2013	Antimicrob Agents Chemother	In vivo therapeutic efficacy of chloroquine alone or in combination with primaquine against vivax malaria in Kolkata, West Bengal, India, and polymorphism in pvmdr1 and pvcrt-o genes.	yes	omission
Timothy S.Walsh[176]	2015	JAMA Intern Med	Increased Hospital-Based Physical Rehabilitation and Information Provision After Intensive Care Unit Discharge: The RECOVER Randomized Clinical Trial.	no	
Peter Hajek[177]	2015	JAMA Intern Med	Increasing varenicline dose in smokers who do not respond to the standard dosage: a randomized clinical trial.	yes	time changes
Bruno Mourvillier[178]	2013	JAMA	Induced hypothermia in severe bacterial meningitis: a randomized clinical trial.	no	
Jacqueline Detert[179]	2013	Ann Rheum Dis	Induction therapy with adalimumab plus methotrexate for 24 weeks followed by methotrexate monotherapy up to week 48 versus methotrexate therapy alone for DMARD-naive patients with early rheumatoid arthritis: HIT HARD, an investigator-initiated study.	no	
R. Ratner[180]	2011	Diabetes Obes Metab	Influence of preprandial vs. postprandial insulin glulisine on weight and glycaemic control in patients initiating basal-bolus regimen for type 2 diabetes: a multicenter, randomized, parallel, open-label study (NCT00135096).	no	
D. Bilton[181]	2011	Eur Respir J	Inhaled dry powder mannitol in cystic fibrosis: an efficacy and safety study.	no	
Gwyneth Vance[182]	2014	Health Policy Plan	Integrating family planning messages into immunization services: a cluster-randomized trial in Ghana and Zambia.	no	
Holger Thiele[183]	2012	Lancet	Intracoronary versus intravenous bolus abciximab during primary percutaneous coronary intervention in patients with acute ST-elevation myocardial infarction: a randomised trial.	no	

J. Curtis Nickel[184]	2012	J Urol	Investigation of a Ca2+ channel Î±2Î´ ligand for the treatment of interstitial cystitis: results of a randomized, double-blind, placebo controlled phase II trial.	no	
F. Stephen Hodi[185]	2014	JAMA	Ipilimumab plus sargramostim vs ipilimumab alone for treatment of metastatic melanoma: a randomized clinical trial.	no	
Claire Harrison[186]	2012	N Engl J Med	JAK inhibition with ruxolitinib versus best available therapy for myelofibrosis.	no	
Judith L. Kristeller[187]	2013	Pharmacotherapy	Lack of effectiveness of sodium bicarbonate in preventing kidney injury in patients undergoing cardiac surgery: a randomized controlled trial.	no	
Janet T. Holbrook[188]	2012	JAMA	Lansoprazole for children with poorly controlled asthma: a randomized controlled trial.	no	
Jean-Paul Galmiche[189]	2011	JAMA	Laparoscopic antireflux surgery vs esomeprazole treatment for chronic GERD: the LOTUS randomized clinical trial.	no	
Gavin J. Murphy[190]	2015	N Engl J Med	Liberal or restrictive transfusion after cardiac surgery.	yes	new outcome
Jeffrey L. Carson[191]	2011	N Engl J Med	Liberal or restrictive transfusion in high-risk patients after hip surgery.	yes	new outcome
N. Østerås[192]	2014	Osteoarthritis Cartilage	Limited effects of exercises in people with hand osteoarthritis: results from a randomized controlled trial.	yes	time changes
Martin Fassnacht[193]	2015	Lancet Oncol	Linsitinib (OSI-906) versus placebo for patients with locally advanced or metastatic adrenocortical carcinoma: a double-blind, randomised, phase 3 study.	no	

Tom Van Herpe[194]	2013	Diabetes Care	LOGIC-insulin algorithm-guided versus nurse-directed blood glucose control during critical illness: the LOGIC-1 single-center, randomized, controlled clinical trial.	no	
Jean-Louis Pepin[195]	2014	Chest	Long-acting bronchodilators and arterial stiffness in patients with COPD: a comparison of fluticasone furoate/vilanterol with tiotropium.	no	
Kenneth L. Subotnik[196]	2015	JAMA Psychiatry	Long-Acting Injectable Risperidone for Relapse Prevention and Control of Breakthrough Symptoms After a Recent First Episode of Schizophrenia. A Randomized Clinical Trial.	yes	seondary to primary
Moira L. Aitken[197]	2012	Am J Respir Crit Care Med	Long-term inhaled dry powder mannitol in cystic fibrosis: an international randomized study.	no	
Roland Tubiana[198]	2013	Clin Infect Dis	Lopinavir/ritonavir monotherapy as a nucleoside analogue-sparing strategy to prevent HIV-1 mother-to-child transmission: the ANRS 135 PRIMEVA phase 2/3 randomized trial.	no	
JamesM. Chamberlain[199]	2014	JAMA	Lorazepam vs diazepam for pediatric status epilepticus: a randomized clinical trial.	no	
Timothy A. Brighton[200]	2012	N Engl J Med	Low-dose aspirin for preventing recurrent venous thromboembolism.	yes	new outcome
Horng H. Chen[201]	2013	JAMA	Low-dose dopamine or low-dose nesiritide in acute heart failure with renal dysfunction: the ROSE acute heart failure randomized trial.	no	
Frank Buttgereit[202]	2013	Ann Rheum Dis	Low-dose prednisone chronotherapy for rheumatoid arthritis: a randomised clinical trial (CAPRA-2).	no	
Joseph Gligorov[203]	2014	Lancet Oncol	Maintenance capecitabine and bevacizumab versus bevacizumab alone after initial first-line bevacizumab and docetaxel for patients with HER2-negative metastatic breast cancer (IMELDA): a randomised, open-label, phase 3 trial.	no	

Roger Stupp[204]	2015	JAMA	Maintenance Therapy With Tumor-Treating Fields Plus Temozolomide vs Temozolomide Alone for Glioblastoma: A Randomized Clinical Trial.	no	
Martine Vercelletto[205]	2011	J Alzheimers Dis	Memantine in behavioral variant frontotemporal dementia: negative results.	no	
Amitabh Chak[206]	2015	Clin Gastroenterol Hepatol	Metformin does not reduce markers of cell proliferation in esophageal tissues of patients with Barrett's esophagus.	no	
Axel Finckh[207]	2014	Arthritis Res Ther	Methotrexate in chronic-recurrent calcium pyrophosphate deposition disease: no significant effect in a randomized crossover trial.	yes	omission
Kate Hallsworth[208]	2015	Clin Sci (Lond)	Modified high-intensity interval training reduces liver fat and improves cardiac function in non-alcoholic fatty liver disease: a randomized controlled trial.	yes	new outcome
Luis Ostrosky-Zeichner[209]	2014	Clin Infect Dis	MSG-01: A randomized, double-blind, placebo-controlled trial of caspofungin prophylaxis followed by preemptive therapy for invasive candidiasis in high-risk adults in the critical care setting.	no	
Carol E. Smith[210]	2014	Heart Rhythm	New image processing and noise reduction technology allows reduction of radiation exposure in complex electrophysiologic interventions while maintaining optimal image quality: a randomized clinical trial.	yes	new outcome
Lukas R.C.Dekker[211]	2013	Gastroenterology	No difference between high-fructose and high-glucose diets on liver triacylglycerol or biochemistry in healthy overweight men.	no	
Alice K. Jacobs[212]	2013	N Engl J Med	Nonemergency PCI at hospitals with or without on-site cardiac surgery.	no	
Jean-Christian Borel[213]	2012	Chest	Noninvasive ventilation in mild obesity hypoventilation syndrome: a randomized controlled trial.	no	

© 2019 Chen T et al. *JAMA Network Open*.

Jan van Lunzen[214]	2012	Lancet Infect Dis	Once daily dolutegravir (S/GSK1349572) in combination therapy in antiretroviral-naive adults with HIV: planned interim 48 week results from SPRING-1, a dose-ranging, randomised, phase 2b trial.	yes	omission
WILLIAM J. SANDBORN[215]	2012	Gastroenterology	Once-daily budesonide MMXÂ® extended-release tablets induce remission in patients with mild to moderate ulcerative colitis: results from the CORE I study.	no	
Nobuya Inagaki[216]	2015	Lancet Diabetes Endocrinol	Once-weekly trelagliptin versus daily alogliptin in Japanese patients with type 2 diabetes: a randomised, double-blind, phase 3, non-inferiority study.	no	
Heikki Joensuu[217]	2012	JAMA	One vs three years of adjuvant imatinib for operable gastrointestinal stromal tumor: a randomized trial.	no	
Tom Adriaenssens[218]	2014	EuroIntervention	Optical coherence tomography study of healing characteristics of paclitaxel-eluting balloons vs. everolimus-eluting stents for in-stent restenosis: the SEDUCE (Safety and Efficacy of a Drug elUting balloon in Coronary artery rEstenosis) randomised clinical trial.	yes	omission
Ambrose Agweyu[219]	2015	Clin Infect Dis	Oral amoxicillin versus benzyl penicillin for severe pneumonia among kenyan children: a pragmatic randomized controlled noninferiority trial.	no	
Shirley S.L. Leung[220]	2011	J Public Health (Oxf)	Outcome of a postnatal depression screening programme using the Edinburgh Postnatal Depression Scale: a randomized controlled trial.	yes	omission
Yong Sang Hong[221]	2014	Lancet Oncol	Oxaliplatin, fluorouracil, and leucovorin versus fluorouracil and leucovorin as adjuvant chemotherapy for locally advanced rectal cancer after preoperative chemoradiotherapy (ADORE): an open-label, multicentre, phase 2, randomised controlled trial.	no	
Monique van Nielen[222]	2014	J Nutr	Partly replacing meat protein with soy protein alters insulin resistance and blood lipids in postmenopausal women with abdominal obesity.	no	
Sara Bristol Calvert[223]	2012	Am Heart J	Patient-focused intervention to improve long-term adherence to evidence-based medications: a randomized trial.	no	

Peter A Calabresi[224]	2014	Lancet Neurol	Pegylated interferon Î²-1a for relapsing-remitting multiple sclerosis (ADVANCE): a randomised, phase 3, double-blind study.	no	
Chen-Hua Liu[225]	2013	Ann Intern Med	Pegylated interferon-Î±2a with or without low-dose ribavirin for treatment-naive patients with hepatitis C virus genotype 1 receiving hemodialysis: a randomized trial.	yes	time changes
Jacqueline A. French[226]	2015	Neurology	Perampanel for tonic-clonic seizures in idiopathic generalized epilepsy A randomized trial.	no	
LCDR Thomas Q. Gallagher[227]	2012	JAMA	Perioperative dexamethasone administration and risk of bleeding following tonsillectomy in children: a randomized controlled trial.	no	
Neena Duggal[228]	2013	Obstet Gynecol	Perioperative oxygen supplementation and surgical site infection after cesarean delivery: a randomized trial.	yes	new outcome
Raffaele Marfella[229]	2012	J Clin Endocrinol Metab	Peri-procedural tight glycemic control during early percutaneous coronary intervention is associated with a lower rate of in-stent restenosis in patients with acute ST-elevation myocardial infarction.	no	
A. Ruzsa[230]	2014	Invest New Drugs	Phase 2, open-label, 1:1 randomized controlled trial exploring the efficacy of EMD 1201081 in combination with cetuximab in second-line cetuximab-naÃ¯ve patients with recurrent or metastatic squamous cell carcinoma of the head and neck (R/M SCCHN).	no	
Andreas Pfu¨ tzner[231]	2011	Diabetes Technol Ther	PIOfix-study: effects of pioglitazone/metformin fixed combination in comparison with a combination of metformin with glimepiride on diabetic dyslipidemia.	no	
Ping-Yen Liu[232]	2013	PLoS One	Pitavastatin and Atorvastatin double-blind randomized comPArative study among hiGh-risk patients, including thOse with Type 2 diabetes mellitus, in Taiwan (PAPAGO-T Study).	no	
Michelle A. O. Kinney[233]	2012	Pain Pract	Preoperative gabapentin for acute post-thoracotomy analgesia: a randomized, double-blinded, active placebo-controlled study.	no	

James M Scheiman[234]	2011	Heart	Prevention of peptic ulcers with esomeprazole in patients at risk of ulcer development treated with low-dose acetylsalicylic acid: a randomised, controlled trial (OBERON).	no	
Ian T. Meredith[235]	2012	J Am Coll Cardiol	Primary endpoint results of the EVOLVE trial: a randomized evaluation of a novel bioabsorbable polymer-coated, everolimus-eluting coronary stent.	no	
Ralph G. Zinner[236]	2015	J Thorac Oncol	PRONOUNCE: randomized, open-label, phase III study of first-line pemetrexed + carboplatin followed by maintenance pemetrexed versus paclitaxel + carboplatin + bevacizumab followed by maintenance bevacizumab in patients ith advanced nonsquamous non-small-cell lung cancer.	no	
Kyoung Heo[237]	2014	J Korean Med Sci	Pro+E1092phylactic effect of erythropoietin injection to prevent acute mountain sickness: an open-label randomized controlled trial.	yes	omission
Mario A. Rojas[238]	2012	Pediatrics	Prophylactic probiotics to prevent death and nosocomial infection in preterm infants.	no	
Roman Prymula[239]	2014	Lancet	Protection against varicella with two doses of combined measles-mumps-rubella-varicella vaccine versus one dose of monovalent varicella vaccine: a multicentre, observer-blind, randomised, controlled trial.	yes	omission
Tim R H Read[240]	2013	BMJ	Provision of rapid HIV tests within a health service and frequency of HIV testing among men who have sex with men: randomised controlled trial.	no	
Diego Galace de Freitas[241]	2014	Arch Phys Med Rehabil	Pulsed electromagnetic field and exercises in patients with shoulder impingement syndrome: a randomized, double-blind, placebo-controlled clinical trial.	yes	new outcome
Gilda Kianimehr[242]	2014	Daru : journal of Faculty of Pharmacy, Tehran University of Medical Sciences	Raloxifene adjunctive therapy for postmenopausal women suffering from chronic schizophrenia: a randomized double-blind and placebo controlled trial.	no	
J.J. De Waele[243]	2013	Int J Antimicrob Agents	Randomised clinical trial of moxifloxacin versus ertapenem in complicated intra-abdominal infections: results of the PROMISE study.	no	

© 2019 Chen T et al. *JAMA Network Open*.

Niels R. Holm[244]	2014	EuroIntervention	Randomised comparison of manual compression and FemoSealâ„¢ vascular closure device for closure after femoral artery access coronary angiography: the CLOSure dEvices Used in everyday Practice (CLOSE-UP) study.	no	
Sharon E Cox[245]	2013	BMJ Open	Randomised controlled trial of weekly chloroquine to re-establish normal erythron iron flux and haemoglobin recovery in postmalarial anaemia.	no	
Paramdeep Singh Bilkhu[246]	2014	Br J Ophthalmol	Randomised masked clinical trial of the MGDRx EyeBag for the treatment of meibomian gland dysfunction-related evaporative dry eye.	yes	new outcome
J Souglakos[247]	2012	Br J Cancer	Randomised phase-II trial of CAPIRI (capecitabine, irinotecan) plus bevacizumab vs FOLFIRI (folinic acid, 5-fluorouracil, irinotecan) plus bevacizumab as first-line treatment of patients with unresectable/metastatic colorectal cancer (mCRC).	yes	new outcome
Patricia Echeverrı´a[248]	2014	PLoS One	Randomised study to assess the efficacy and safety of once-daily etravirine-based regimen as a switching strategy in HIV-infected patients receiving a protease inhibitor-containing regimen. Etraswitch study.	no	
A. M. Wolthuis[249]	2015	Br J Surg	Randomized clinical trial of laparoscopic colectomy with or without natural-orifice specimen extraction.	no	
L. S. Sorensen[250]	2013	Br J Surg	Randomized clinical trial of perioperative omega-3 fatty acid supplements in elective colorectal cancer surgery.	no	
Ning Liu[251]	2014	Stroke	Randomized controlled trial of early rehabilitation after intracerebral hemorrhage stroke: difference in outcomes within 6 months of stroke.	no	
Yi-Hsiang Huang[252]	2013	J Clin Oncol	Randomized controlled trial of entecavir prophylaxis for rituximab-associated hepatitis B virus reactivation in patients with lymphoma and resolved hepatitis B.	no	
P. T. McCollum[253]	2011	Br J Surg	Randomized phase II clinical trial of avotermin versus placebo for scar improvement.	yes	omission

Chigusa Morizane[254]	2013	Cancer Sci	Randomized phase II study of gemcitabine plus S-1 versus S-1 in advanced biliary tract cancer: a Japan Clinical Oncology Group trial (JCOG 0805).	no	
Robert D Reid[255]	2011	Eur J Prev Cardiol	Randomized trial of an internet-based computer-tailored expert system for physical activity in patients with heart disease.	yes	omission
Jean-Pierre Bronowicki[256]	2014	J Hepatol	Randomized trial of asunaprevir plus peginterferon alfa and ribavirin for previously untreated genotype 1 or 4 chronic hepatitis C.	no	
Nathalie Bocquet[257]	2012	Pediatrics	Randomized trial of oral versus sequential IV/oral antibiotic for acute pyelonephritis in children.	no	
K. Steindorf[258]	2014	Ann Oncol	Randomized, controlled trial of resistance training in breast cancer patients receiving adjuvant radiotherapy: results on cancer-related fatigue and quality of life.	no	
Peter V. Draganov[259]	2012	Gastrointest Endosc	Randomized, controlled trial of standard, large-capacity versus jumbo biopsy forceps for polypectomy of small, sessile, colorectal polyps.	no	
David Oldach[260]	2013	Antimicrob Agents Chemother	Randomized, double-blind, multicenter phase 2 study comparing the efficacy and safety of oral solithromycin (CEM-101) to those of oral levofloxacin in the treatment of patients with community-acquired bacterial pneumonia.	no	
Martin Bornhäuser[261]	2012	Lancet Oncol	Reduced-intensity conditioning versus standard conditioning before allogeneic haemopoietic cell transplantation in patients with acute myeloid leukaemia in first complete remission: a prospective, open-label randomised phase 3 trial.	no	
Kristina Dupont Hougaard[262]	2014	Stroke	Remote ischemic perconditioning as an adjunct therapy to thrombolysis in patients with acute ischemic stroke: a randomized trial.	yes	new outcome
Timo Vesikari[263]	2011	Clin Vaccine Immunol	Results from a randomized clinical trial of coadministration of RotaTeq, a pentavalent rotavirus vaccine, and NeisVac-C, a meningococcal serogroup C conjugate vaccine.	no	

Cheryl L. Rock[264]	2015	J Clin Oncol	Results of the Exercise and Nutrition to Enhance Recovery and Good Health for You (ENERGY) Trial: A Behavioral Weight Loss Intervention in Overweight or Obese Breast Cancer Survivors.	no	
Sanne M. van der Made[265]	2015	PLoS One	Resveratrol does not influence metabolic risk markers related to cardiovascular health in overweight and slightly obese subjects: a randomized, placebo-controlled crossover trial.	no	
Te-Fa Chiu[266]	2013	BMC Complement Altern Med	Rhodiola crenulata extract for prevention of acute mountain sickness: a randomized, double-blind, placebo-controlled, crossover trial.	no	
Timothy Iveson[267]	2014	Lancet Oncol	Rilotumumab in combination with epirubicin, cisplatin, and capecitabine as first-line treatment for gastric or oesophagogastric junction adenocarcinoma: an open-label, dose de-escalation phase 1b study and a double-blind, randomised phase 2 study.	no	
Jolien E J Boesten[268]	2012	Fam Pract	Rimonabant improves obesity but not the overall cardiovascular risk and quality of life; results from CARDIO-REDUSE (CArdiometabolic Risk reDuctIOn by Rimonabant: the Effectiveness in Daily practice and its USE).	yes	omission
Hossein-Ardeschir Ghofrani[269]	2013	N Engl J Med	Riociguat for the treatment of chronic thromboembolic pulmonary hypertension.	no	
L. Guillevin[270]	2014	N Engl J Med	Rituximab versus azathioprine for maintenance in ANCA-associated vasculitis.	no	
David Devos[271]	2013	J Neurol Neurosurg Psychiatry	Rivastigmine in apathetic but dementia and depression-free patients with Parkinson's disease: a double-blind, placebo-controlled, randomised clinical trial.	no	
Lorenzo Capussotti[272]	2012	J Gastrointest Surg	Routine anterior approach during right hepatectomy: results of a prospective randomised controlled trial.	no	
J M Munster[273]	2013	Euro Surveill	Routine screening for Coxiella burnetii infection during pregnancy: a clustered randomised controlled trial during an outbreak, the Netherlands, 2010.	no	

© 2019 Chen T et al. *JAMA Network Open*.

J. McCready[274]	2014	Europace	Safety and efficacy of multipolar pulmonary vein ablation catheter vs. irrigated radiofrequency ablation for paroxysmal atrial fibrillation: a randomized multicentre trial.	yes	omission
W. Rigby[275]	2012	Arthritis Rheum	Safety and efficacy of ocrelizumab in patients with rheumatoid arthritis and an inadequate response to methotrexate: results of a forty-eight-week randomized, double-blind, placebo-controlled, parallel-group phase III trial.	no	
GE Gray[276]	2011	Lancet Infect Dis	Safety and efficacy of the HVTN 503/Phambili study of a clade-B-based HIV-1 vaccine in South Africa: a double-blind, randomised, placebo-controlled test-of-concept phase 2b study.	no	
Mario Siebler[277]	2011	Stroke	Safety of Tirofiban in acute Ischemic Stroke: the SaTIS trial.	no	
Olivier Sitbon[278]	2015	N Engl J Med	Selexipag for the Treatment of Pulmonary Arterial Hypertension.	no	
Manna Zhang[279]	2015	J Clin Endocrinol Metab	Sequential Versus Continual Purified Urinary FSH/hCG in Men With Idiopathic Hypogonadotropic Hypogonadism.	no	
Rachel Jenkins[280]	2013	International Journal of Mental Health Systems	Short structured general mental health in service training programme in Kenya improves patient health and social outcomes but not detection of mental health problems - a pragmatic cluster randomised controlled trial.	yes	new outcomes
Arkaitz Imaz[281]	2013	Antivir Ther	Short-term and long-term clinical and immunological consequences of stopping antiretroviral therapy in HIV-infected patients with preserved immune function.	yes	new outcome
James W Denham[282]	2014	Lancet Oncol	Short-term androgen suppression and radiotherapy versus intermediate-term androgen suppression and radiotherapy, with or without zoledronic acid, in men with locally advanced prostate cancer (TROG 03.04 RADAR): an open-label, randomised, phase 3 factorial trial.	yes	omission
Mads J. Andersen[283]	2013	Circulation	Sildenafil and diastolic dysfunction after acute myocardial infarction in patients with preserved ejection fraction: the Sildenafil and Diastolic Dysfunction After Acute Myocardial Infarction (SIDAMI) trial.	yes	omission

© 2019 Chen T et al. *JAMA Network Open*.

Seung Tae Kim[284]	2014	Eur J Cancer	Simvastatin plus capecitabine-cisplatin versus placebo plus capecitabine-cisplatin in patients with previously untreated advanced gastric cancer: a double-blind randomised phase 3 study.	no	
S. Friman[285]	2011	Am J Transplant	Sotrastaurin, a novel small molecule inhibiting protein-kinase C: randomized phase II study in renal transplant recipients.	no	
Ian M Gralnek[286]	2014	Lancet Oncol	Standard forward-viewing colonoscopy versus full-spectrum endoscopy: an international, multicentre, randomised, tandem colonoscopy trial.	yes	new outcome
Ashlesha Patel[287]	2014	Vaccine	Staying on track: a cluster randomized controlled trial of automated reminders aimed at increasing human papillomavirus vaccine completion.	yes	time changes
Edoardo Camenzind[288]	2012	Lancet	Stent thrombosis and major clinical events at 3 years after zotarolimus-eluting or sirolimus-eluting coronary stent implantation: a randomised, multicentre, open-label, controlled trial.	no	
ALAN KIVITZ[289]	2014	Arthritis Care Res (Hoboken)	Subcutaneous tocilizumab versus placebo in combination with disease-modifying antirheumatic drugs in patients with rheumatoid arthritis.	no	
P.K. Smith[290]	2014	N Engl J Med	Surgical treatment of moderate ischemic mitral regurgitation.	no	
A. Dhanya Mackeen[291]	2014	Obstet Gynecol	Suture compared with staple skin closure after cesarean delivery: a randomized controlled trial.	yes	new outcome
Gonzague Jourdain[292]	2013	PLoS Med	Switching HIV treatment in adults based on CD4 count versus viral load monitoring: a randomized, non-inferiority trial in Thailand.	no	
Yong-Chul Kim[293]	2013	PLoS One	Tacrolimus decreases albuminuria in patients with IgA nephropathy and normal blood pressure: a double-blind randomized controlled trial of efficacy of tacrolimus on IgA nephropathy.	no	

© 2019 Chen T et al. *JAMA Network Open*.

David Biondi[294]	2013	Pain Physician	Tapentadol immediate release versus oxycodone immediate release for treatment of acute low back pain.	yes	time changes
Lisanne M Verweij[295]	2012	Occup Environ Med	The application of an occupational health guideline reduces sedentary behaviour and increases fruit intake at work: results from an RCT.	yes	omission
George Ou[296]	2014	Gastrointest Endosc	The effect of chewing gum on small-bowel transit time in capsule endoscopy: a prospective, randomized trial.	no	
Ilsoon Son[297]	2014	ScientificWorldJournal	The effect of sufentanil administration on remifentanil-based anaesthesia during laparoscopic gynaecological surgery: a double-blind randomized controlled trial.	yes	omission
Brian R Leaker[298]	2014	BMC Pulm Med	The effect of the novel phosphodiesterase-4 inhibitor MEM 1414 on the allergen induced responses in mild asthma.	yes	new outcome
Julie Kinley[299]	2014	Palliat Med	The effect of using high facilitation when implementing the Gold Standards Framework in Care Homes programme: a cluster randomised controlled trial.	no	
James A. Blumenthal[300]	2014	Psychosom Med	The effects of a telehealth coping skills intervention on outcomes in chronic obstructive pulmonary disease: primary results from the INSPIRE-II study.	no	
Eun Bong Lee[301]	2014	N Engl J Med	Tofacitinib versus methotrexate in rheumatoid arthritis.	yes	omission
Silvio Danese[302]	2015	Gut	Tralokinumab for moderate-to-severe UC: a randomised, double-blind, placebo-controlled, phase IIa study.	no	
BW Prick[303]	2014	BJOG	Transfusion policy after severe postpartum haemorrhage: a randomised non-inferiority trial.	no	

W. Jack Rejeski[304]	2011	Arch Intern Med	Translating weight loss and physical activity programs into the community to preserve mobility in older, obese adults in poor cardiovascular health.	yes	new outcome
Maggie C Walter[305]	2013	Orphanet J Rare Dis	Treatment of dysferlinopathy with deflazacort: a double-blind, placebo-controlled clinical trial.	no	
Ploenchan Chetchotisakd[306]	2014	Lancet	Trimethoprim-sulfamethoxazole versus trimethoprim-sulfamethoxazole plus doxycycline as oral eradicative treatment for melioidosis (MERTH): a multicentre, double-blind, non-inferiority, randomised controlled trial.	yes	omission
Marieke L Duiverman[307]	2011	Respir Res	Two-year home-based nocturnal noninvasive ventilation added to rehabilitation in chronic obstructive pulmonary disease patients: a randomized controlled trial.	no	
Lucy C Chappell[308]	2012	BMJ	Ursodeoxycholic acid versus placebo, and early term delivery versus expectant management, in women with intrahepatic cholestasis of pregnancy: semifactorial randomised clinical trial.	no	
John B. Buse[309]	2011	Ann Intern Med	Use of twice-daily exenatide in Basal insulin-treated patients with type 2 diabetes: a randomized, controlled trial.	no	
Tae Joon Yi[310]	2014	J Infect Dis	Valacyclovir therapy does not reverse herpes-associated alterations in cervical immunology: a randomized, placebo-controlled crossover trial.	yes	time changes
Michael Larsen[311]	2012	Ophthalmology	Verteporfin plus ranibizumab for choroidal neovascularization in age-related macular degeneration: twelve-month MONT BLANC study results.	no	
Tzu-Chieh Yu[312]	2013	Ann Surg	Warm, humidified carbon dioxide gas insufflation for laparoscopic appendicectomy in children: a double-blinded randomized controlled trial.	yes	omission
Marco Valgimigli[313]	2015	J Am Coll Cardiol	Zotarolimus-eluting versus bare-metal stents in uncertain drug-eluting stent candidates.	no	

© 2019 Chen T et al. *JAMA Network Open*.

John G.F. Cleland[314]	2011	Eur J Heart Fail	The Heart Failure Revascularisation Trial (HEART).	no	
Fa´bio Coelho Guarany[315]	2013	PLoS One	A double-blind, randomised, crossover trial of two botulinum toxin type a in patients with spasticity.	no	
N. Blanch[316]	2014	Nutr Metab Cardiovasc Dis	Effect of high potassium diet on endothelial function.	no	
Kevin C. Maki[317]	2011	J Cardiovasc Pharmacol	Effects of prescription omega-3-acid ethyl esters on fasting lipid profile in subjects with primary hypercholesterolemia.	yes	omission
Ari Shechter[318]	2013	Am J Clin Nutr	Experimental sleep curtailment causes wake-dependent increases in 24-h energy expenditure as measured by whole-room indirect calorimetry.	yes	omission
Elizabeth A Green[319]	2014	Cardiovasc Ther	Melatonin reduces tachycardia in postural tachycardia syndrome: a randomized, crossover trial.	no	
Helen Vlassara[320]	2012	Clin J Am Soc Nephrol	Effects of sevelamer on HbA1c, inflammation, and advanced glycation end products in diabetic kidney disease.	yes	omission
A.Nadjarzadeh[321]	2011	J Endocrinol Invest	Coenzyme Q10 improves seminal oxidative defense but does not affect on semen parameters in idiopathic oligoasthenoteratozoospermia: a randomized double-blind, placebo controlled trial.	yes	omission
Nasrin Saharkhiz[322]	2014	International Journal of Fertility and Sterility	Comparison between conventional blind embryo transfer and embryo transfer based on previously measured uterine length.	yes	omission
Mads Buhl[323]	2013	J Clin Endocrinol Metab	Direct effects of locally administered lipopolysaccharide on glucose, lipid, and protein metabolism in the placebo-controlled, bilaterally infused human leg.	yes	omission

© 2019 Chen T et al. *JAMA Network Open*.

Morten Petersen[324]	2012	Clin Exp Pharmacol Physiol	Effect of the Arg389Gly Î²â‚ -adrenoceptor polymorphism on plasma renin activity and heart rate, and the genotype-dependent response to metoprolol treatment.	yes	omission
Deok Man Hong[325]	2012	Circ J	Effects of remote ischemic preconditioning with postconditioning in patients undergoing off-pump coronary artery bypass surgery--randomized controlled trial.	no	
Ellen L. Poleshuck[326]	2014	J Psychosom Res	Randomized controlled trial of interpersonal psychotherapy versus enhanced treatment as usual for women with co-occurring depression and pelvic pain.	no	
Kamalesh J. Ramaiya[327]	2013	Ophthalmic Surg Lasers Imaging Retina	Ranibizumab versus photodynamic therapy for presumed ocular histoplasmosis syndrome.	no	
Sarojini Maria Dos Remedios Monteiro[328]	2014	Prev Med	Results of a randomized controlled trial to promote physical activity behaviours in mothers with young children.	yes	omission
Marc-Dennan Tensil[329]	2013	J Med Internet Res	Two fully automated web-based interventions for risky alcohol use: randomized controlled trial.	yes	omission
Eva Van Braeckel[330]	2011	Clin Infect Dis	An adjuvanted polyprotein HIV-1 vaccine induces polyfunctional cross-reactive CD4+ T cell responses in seronegative volunteers	yes	omission
R Landewé[331]	2013	Ann Rheum Dis	Efficacy of certolizumab pegol on signs and symptoms of axial spondyloarthritis including ankylosing spondylitis: 24-week results of a double-blind randomised placebo-controlled Phase 3 study.	no	
BRUCE E. SANDS[332]	2012	Gastroenterology	Basiliximab does not increase efficacy of corticosteroids in patients with steroid-refractory ulcerative colitis.	yes	new outcome
Banny S. Wong[333]	2011	Am J Gastroenterol	Effects of A3309, an ileal bile acid transporter inhibitor, on colonic transit and symptoms in females with functional constipation.	no	

Otilia-Constantina Rogoveanu[334]	2015	Biol Trace Elem Res	Effects of Calcium Fructoborate on Levels of C-Reactive Protein, Total Cholesterol, Low-Density Lipoprotein, Triglycerides, IL-1Î², IL-6, and MCP-1: a Double-blind, Placebo-controlled Clinical Study.	yes	omission
Edward M. Kerwin[335]	2012	COPD	Efficacy and safety of a 12-week treatment with twice-daily aclidinium bromide in COPD patients (ACCORD COPD I).	no	
Vik Khullar[336]	2013	Eur Urol	Efficacy and tolerability of mirabegron, a Î²(3)-adrenoceptor agonist, in patients with overactive bladder: results from a randomised European-Australian phase 3 trial.	no	
PING–I HSU[337]	2011	Gastroenterology	Esomeprazole with clopidogrel reduces peptic ulcer recurrence, compared with clopidogrel alone, in patients with atherosclerosis.	no	
Julian M. Somers[338]	2013	PLoS One	Housing first reduces re-offending among formerly homeless adults with mental disorders: results of a randomized controlled trial.	yes	new outcome
L.A. Ferrara[339]	2015	Nutr Metab Cardiovasc Dis	Low-lipid diet reduces frequency and severity of acute migraine attacks.	yes	omission
Michael A Roussell[340]	2012	Am J Clin Nutr	Beef in an Optimal Lean Diet study: effects on lipids, lipoproteins, and apolipoproteins.	yes	omission
T. R. Andersen[341]	2014	Scand J Med Sci Sports	Effect of football or strength training on functional ability and physical performance in untrained old men.	yes	omission
Adrienne Birnbaum[342]	2012	Acad Emerg Med	Efficacy of patient-controlled analgesia for patients with acute abdominal pain in the emergency department: a randomized trial.	yes	omission
Allison E. Aiello[343]	2012	PLoS One	Facemasks, hand hygiene, and influenza among young adults: a randomized intervention trial.	yes	new outcome

© 2019 Chen T et al. *JAMA Network Open*.

Patricia M. Flynn[344]	2011	J Acquir Immune Defic Syndr	Hepatitis B vaccination in HIV-infected youth: a randomized trial of three regimens.	no	
Lindy Clemson[345]	2012	BMJ	Integration of balance and strength training into daily life activity to reduce rate of falls in older people (the LiFE study): randomised parallel trial.	no	
André Robidoux[346]	2013	Lancet Oncol	Lapatinib as a component of neoadjuvant therapy for HER2-positive operable breast cancer (NSABP protocol B-41): an open-label, randomised phase 3 trial.	no	
Joachim von Pawel[347]	2014	Clin Lung Cancer	Phase II trial of mapatumumab, a fully human agonist monoclonal antibody to tumor necrosis factor-related apoptosis-inducing ligand receptor 1 (TRAIL-R1), in combination with paclitaxel and carboplatin in patients with advanced non-small-cell lung cancer.	no	
Tanya Simuni[348]	2015	Lancet Neurol	Pioglitazone in early Parkinson's disease: a phase 2, multicentre, double-blind, randomised trial.	no	
Tonine Bartelmaos[349]	2013	Transfusion	Plasma transfusion in liver transplantation: a randomized, double-blind, multicenter clinical comparison of three virally secured plasmas.	no	
Masunori Matsuzaki[350]	2011	J Hypertens	Prevention of cardiovascular events with calcium channel blocker-based combination therapies in patients with hypertension: a randomized controlled trial.	no	
Michael J Yelland[351]	2011	Br J Sports Med	Prolotherapy injections and eccentric loading exercises for painful Achilles tendinosis: a randomised trial.	yes	time changes
Deborah S. Hasin[352]	2013	Addiction	Reducing heavy drinking in HIV primary care: a randomized trial of brief intervention, with and without technological enhancement.	yes	omission
Veerachai Watanaveeradej[353]	2014	Am J Trop Med Hyg	Safety and immunogenicity of a rederived, live-attenuated dengue virus vaccine in healthy adults living in Thailand: a randomized trial.	yes	omission

Jacques p. Barber[354]	2012	J Clin Psychiatry	Short-term dynamic psychotherapy versus pharmacotherapy for major depressive disorder: a randomized, placebo-controlled trial.	yes	omission
Paul Emery[355]	2014	N Engl J Med	Sustained remission with etanercept tapering in early rheumatoid arthritis.	no	
Sylvie Négrier[356]	2011	Lancet Oncol	Temsirolimus and bevacizumab, or sunitinib, or interferon alfa and bevacizumab for patients with advanced renal cell carcinoma (TORAVA): a randomised phase 2 trial.	no	
Mitsuru Adachi[357]	2012	J Asthma	The efficacy and tolerability of intravenous montelukast in acute asthma exacerbations in Japanese patients.	no	
Mary Kay Koenig[358]	2012	Drugs R D	Topical rapamycin therapy to alleviate the cutaneous manifestations of tuberous sclerosis complex: a double-blind, randomized, controlled trial to evaluate the safety and efficacy of topically applied rapamycin.	yes	omission
Jacques Donnez[359]	2012	N Engl J Med	Ulipristal acetate versus leuprolide acetate for uterine fibroids.	no	
Kishore M. Gadde[360]	2012	Arch Intern Med	Zonisamide for weight reduction in obese adults: a 1-year randomized controlled trial.	no	
Donald A. Mahler[361]	2014	Eur Respir J	Dual bronchodilation with QVA149 reduces patient-reported dyspnoea in COPD: the BLAZE study.	no	
Paul L.Jacobsen[362]	2015	J Clin Psychiatry	A randomized, double-blind, placebo-controlled study of the efficacy and safety of 2 doses of vortioxetine in adults with major depressive disorder.	no	
Chuan-Mo Lee[363]	2014	J Interferon Cytokine Res	A double-blind randomized controlled study to evaluate the efficacy of low-dose oral interferon-alpha in preventing hepatitis C relapse.	no	

Richard D. Blondell[364]	2011	J Addict Dis	A randomized trial of two behavioral interventions to improve outcomes following inpatient detoxification for alcohol dependence.	no	
Christian Torp-Pedersen[365]	2011	Circ Arrhythm Electrophysiol	A randomized, placebo-controlled study of vernakalant (oral) for the prevention of atrial fibrillation recurrence after cardioversion.	no	
Lori A. Bateman[366]	2011	Am J Cardiol	Comparison of aerobic versus resistance exercise training effects on metabolic syndrome (from the Studies of a Targeted Risk Reduction Intervention Through Defined Exercise - STRRIDE-AT/RT).	yes	omission
Richard D. Blondell[367]	2011	Respir Med	Contrasting breathing retraining and helium-oxygen during pulmonary rehabilitation in COPD: a randomized clinical trial.	no	
Michal G Ison [368]	2012	Antivir Ther	Efficacy and safety of oral oseltamivir for influenza prophylaxis in transplant recipients.	no	
William C. Cushman[369]	2012	Hypertension	Azilsartan medoxomil plus chlorthalidone reduces blood pressure more effectively than olmesartan plus hydrochlorothiazide in stage 2 systolic hypertension.	no	
Christopher W Teshima[370]	2014	World J Gastroenterol	Magnetic imaging-assisted colonoscopy vs conventional colonoscopy: a randomized controlled trial.	yes	new outcome
N. Galiè[371]	2015	N Engl J Med	Initial Use of Ambrisentan plus Tadalafil in Pulmonary Arterial Hypertension.	no	
Ragaa Mansour[372]	2011	Fertil Steril	Intrauterine injection of human chorionic gonadotropin before embryo transfer significantly improves the implantation and pregnancy rates in inÂ vitro fertilization/intracytoplasmic sperm injection: a prospective randomized study.	no	
Guoqing Qian[373]	2012	PLoS One	Mitigation of oxidative damage by green tea polyphenols and Tai Chi exercise in postmenopausal women with osteopenia.	yes	new outcome

© 2019 Chen T et al. *JAMA Network Open*.

Kevin Patrick[374]	2013	J Diabetes Sci Technol	Outcomes of a 12-month technology-based intervention to promote weight loss in adolescents at risk for type 2 diabetes.	no	
Norman A Constantine[375]	2015	BMC Public Health	Short-term effects of a rights-based sexuality education curriculum for high-school students: a cluster-randomized trial.	yes	omission
Tia M Rains[376]	2015	Nutr J	A randomized, controlled, crossover trial to assess the acute appetitive and metabolic effects of sausage and egg-based convenience breakfast meals in overweight premenopausal women.	no	
Benjamin Speich[377]	2012	PLoS Negl Trop Dis	Efficacy and safety of nitazoxanide, albendazole, and nitazoxanide-albendazole against Trichuris trichiura infection: a randomized controlled trial.	yes	omission
Krysia Dziedzic[378]	2015	Ann Rheum Dis	Self-management approaches for osteoarthritis in the hand: a 2Ã—2 factorial randomised trial.	no	
George Du Toit[379]	2015	N Engl J Med	Randomized trial of peanut consumption in infants at risk for peanut allergy.	no	
Michael P. Diamond[380]	2014	Journal of Endometriosis	Treatment of endometriosis-associated pain with elagolix, an oral GnRH antagonist: Results from a phase 2, randomized controlled study.	yes	time change
Said A. Ibrahim[381]	2013	Arthritis Rheum	Willingness and access to joint replacement among African American patients with knee osteoarthritis: a randomized, controlled intervention.	no	
Michael W. Fried[382]	2013	Hepatology	Once-daily simeprevir (TMC435) with pegylated interferon and ribavirin in treatment-naÃ¯ve genotype 1 hepatitis C: the randomized PILLAR study.	yes	new outcome
R. L. Dobbins[383]	2013	Diabetes Obes Metab	GSK256073, a selective agonist of G-protein coupled receptor 109A (GPR109A) reduces serum glucose in subjects with type 2 diabetes mellitus.	no	

Jeanne M. Marrazzo[384]	2015	N Engl J Med	Tenofovir-based preexposure prophylaxis for HIV infection among African women.	no	
Fernando J. Martinez[385]	2013	Respir Med	Fluticasone furoate/vilanterol (100/25; 200/25 Î¼g) improves lung function in COPD: a randomised trial.	no	
Gemma Chiva-Blanch[386]	2013	Clin Nutr	Effects of red wine polyphenols and alcohol on glucose metabolism and the lipid profile: a randomized clinical trial.	yes	omission
Joseph E. Donnelly[387]	2013	Obesity (Silver Spring)	Aerobic exercise alone results in clinically significant weight loss for men and women: midwest exercise trial 2.	yes	new outcome
Harold E. Bays[388]	2013	Am J Cardiol	Efficacy and safety of ezetimibe added to atorvastatin versus atorvastatin uptitration or switching to rosuvastatin in patients with primary hypercholesterolemia.	no	
A. P. Sykes[389]	2016	Diabetes Obes Metab	Randomized efficacy and safety trial of once-daily remogliflozin etabonate for the treatment of type 2 diabetes.	no	
H. Asita de Silva[390]	2011	PLoS Med	Low-dose adrenaline, promethazine, and hydrocortisone in the prevention of acute adverse reactions to antivenom following snakebite: a randomised, double-blind, placebo-controlled trial.	yes	new outcome
Jeffrey T. Kullgren[391]	2014	Ann Intern Med	Financial incentives for completion of fecal occult blood tests among veterans: a 2-stage, pragmatic, cluster, randomized, controlled trial.	no	
Rakesh Lodha[392]	2014	Am J Clin Nutr	Effect of micronutrient supplementation on treatment outcomes in children with intrathoracic tuberculosis: a randomized controlled trial.	no	
Rakesh Lodha[393]	2014	Eur J Anaesthesiol	Low-dose neostigmine to antagonise shallow atracurium neuromuscular block during inhalational anaesthesia: A randomised controlled trial.	no	

© 2019 Chen T et al. *JAMA Network Open*.

4. Reference:

1. Abraha I, Cherubini A, Cozzolino F, et al. Deviation from intention to treat analysis in randomised trials and treatment effect estimates: meta-epidemiological study. *BMJ.* 2015;350:h2445.
2. Nuesch E, Trelle S, Reichenbach S, et al. The effects of excluding patients from the analysis in randomised controlled trials: meta-epidemiological study. *BMJ.* 2009;339:b3244.
3. Nuesch E, Trelle S, Reichenbach S, et al. Small study effects in meta-analyses of osteoarthritis trials: meta-epidemiological study. *BMJ.* 2010;341:c3515.
4. Bafeta A, Dechartres A, Trinquart L, Yavchitz A, Boutron I, Ravaud P. Impact of single centre status on estimates of intervention effects in trials with continuous outcomes: meta-epidemiological study. *BMJ.* 2012;344:e813.
5. Cognetti F, Bagnato A, Colombo N, et al. A Phase II, randomized, double-blind study of zibotentan (ZD4054) in combination with carboplatin/paclitaxel versus placebo in combination with carboplatin/paclitaxel in patients with advanced ovarian cancer sensitive to platinum-based chemotherapy (AGO-OVAR 2.14). *Gynecol Oncol.* 2013;130(1):31-37.
6. Lim AC, Schuit E, Bloemenkamp K, et al. 17alpha-hydroxyprogesterone caproate for the prevention of adverse neonatal outcome in multiple pregnancies: a randomized controlled trial. *Obstet Gynecol.* 2011;118(3):513-520.
7. Lees CC, Marlow N, van Wassenaer-Leemhuis A, et al. 2 year neurodevelopmental and intermediate perinatal outcomes in infants with very preterm fetal growth restriction (TRUFFLE): a randomised trial. *Lancet.* 2015;385(9983):2162-2172.
8. Ngandu T, Lehtisalo J, Solomon A, et al. A 2 year multidomain intervention of diet, exercise, cognitive training, and vascular risk monitoring versus control to prevent cognitive decline in at-risk elderly people (FINGER): a randomised controlled trial. *Lancet.* 2015;385(9984):2255-2263.
9. Kim J, Ryu H, Yoo DH, et al. A clinical trial and extension study of infliximab in Korean patients with active rheumatoid arthritis despite methotrexate treatment. *J Korean Med Sci.* 2013;28(12):1716-1722.
10. Skolnick BE, Maas AI, Narayan RK, et al. A clinical trial of progesterone for severe traumatic brain injury. *N Engl J Med.* 2014;371(26):2467-2476.
11. Keyserling TC, Sheridan SL, Draeger LB, et al. A comparison of live counseling with a web-based lifestyle and medication intervention to reduce coronary heart disease risk: a randomized clinical trial. *JAMA Intern Med.* 2014;174(7):1144-1157.
12. Nielsen JC, Thomsen PE, Hojberg S, et al. A comparison of single-lead atrial pacing with dual-chamber pacing in sick sinus syndrome. *Eur Heart J.* 2011;32(6):686-696.
13. Talavera JO, Martinez G, Cervantes JL, et al. A double-blind, double-dummy, randomized, placebo-controlled trial to evaluate the effect of statin therapy on triglyceride levels in Mexican hypertriglyceridemic patients. *Curr Med Res Opin.* 2013;29(4):379-386.
14. Teoh AY, Chiu PW, Wong TC, et al. A double-blinded randomized controlled trial of laparoendoscopic single-site access versus conventional 3-port appendectomy. *Ann Surg.* 2012;256(6):909-914.

© 2019 Chen T et al. *JAMA Network Open*.

15. Ramon-Krauel M, Salsberg SL, Ebbeling CB, et al. A low-glycemic-load versus low-fat diet in the treatment of fatty liver in obese children. *Child Obes.* 2013;9(3):252-260.
16. Yoo HK, Joung YS, Lee JS, et al. A multicenter, randomized, double-blind, placebo-controlled study of aripiprazole in children and adolescents with Tourette's disorder. *J Clin Psychiatry.* 2013;74(8):e772-780.
17. Quartier P, Allantaz F, Cimaz R, et al. A multicentre, randomised, double-blind, placebo-controlled trial with the interleukin-1 receptor antagonist anakinra in patients with systemic-onset juvenile idiopathic arthritis (ANAJIS trial). *Ann Rheum Dis.* 2011;70(5):747-754.
18. Maki DG, Ash SR, Winger RK, Lavin P, Investigators AT. A novel antimicrobial and antithrombotic lock solution for hemodialysis catheters: a multi-center, controlled, randomized trial. *Crit Care Med.* 2011;39(4):613-620.
19. Chen AC, Martin AJ, Choy B, et al. A Phase 3 Randomized Trial of Nicotinamide for Skin-Cancer Chemoprevention. *N Engl J Med.* 2015;373(17):1618-1626.
20. Doody RS, Raman R, Farlow M, et al. A phase 3 trial of semagacestat for treatment of Alzheimer's disease. *N Engl J Med.* 2013;369(4):341-350.
21. Nanchahal K, Power T, Holdsworth E, et al. A pragmatic randomised controlled trial in primary care of the Camden Weight Loss (CAMWEL) programme. *BMJ Open.* 2012;2(3).
22. Sarr MG, Hutcher NE, Snyder S, Hodde J, Carmody B. A prospective, randomized, multicenter trial of Surgisis Gold, a biologic prosthetic, as a sublay reinforcement of the fascial closure after open bariatric surgery. *Surgery.* 2014;156(4):902-908.
23. Lall R, Hamilton P, Young D, et al. A randomised controlled trial and cost-effectiveness analysis of high-frequency oscillatory ventilation against conventional artificial ventilation for adults with acute respiratory distress syndrome. The OSCAR (OSCillation in ARDS) study. *Health Technol Assess.* 2015;19(23):1-177, vii.
24. Roberts LM, McCahon D, Holder R, Wilson S, Hobbs FD. A randomised controlled trial of a probiotic 'functional food' in the management of irritable bowel syndrome. *BMC Gastroenterol.* 2013;13:45.
25. Venkitaraman R, Lorente D, Murthy V, et al. A randomised phase 2 trial of dexamethasone versus prednisolone in castration-resistant prostate cancer. *Eur Urol.* 2015;67(4):673-679.
26. Sands BE, Katz S, Wolf DC, et al. A randomised, double-blind, sham-controlled study of granulocyte/monocyte apheresis for moderate to severe Crohn's disease. *Gut.* 2013;62(9):1288-1294.
27. Yeoh T, Hayward C, Benson V, et al. A randomised, placebo-controlled trial of carvedilol in early familial dilated cardiomyopathy. *Heart Lung Circ.* 2011;20(9):566-573.
28. Kim H, Lee JH, Joo YD, et al. A randomized comparison of cyclophosphamide vs. reduced dose cyclophosphamide plus fludarabine for allogeneic hematopoietic cell transplantation in patients with aplastic anemia and hypoplastic myelodysplastic syndrome. *Ann Hematol.* 2012;91(9):1459-1469.
29. Stollman N, Magowan S, Shanahan F, Quigley EM, Group DI. A randomized controlled study of mesalamine after acute diverticulitis: results of the DIVA trial. *J Clin Gastroenterol.* 2013;47(7):621-629.
30. Volandes AE, Brandeis GH, Davis AD, et al. A randomized controlled trial of a goals-of-care video for elderly patients admitted to skilled nursing facilities. *J Palliat Med.* 2012;15(7):805-811.

© 2019 Chen T et al. *JAMA Network Open*.

31. Farrar MD, Nicolaou A, Clarke KA, et al. A randomized controlled trial of green tea catechins in protection against ultraviolet radiation-induced cutaneous inflammation. *Am J Clin Nutr.* 2015;102(3):608-615.
32. Phelan KJ, Khoury J, Xu Y, Liddy S, Hornung R, Lanphear BP. A randomized controlled trial of home injury hazard reduction: the HOME injury study. *Arch Pediatr Adolesc Med.* 2011;165(4):339-345.
33. Parikh SV, Zaretsky A, Beaulieu S, et al. A randomized controlled trial of psychoeducation or cognitive-behavioral therapy in bipolar disorder: a Canadian Network for Mood and Anxiety treatments (CANMAT) study [CME]. *J Clin Psychiatry.* 2012;73(6):803-810.
34. Takahashi PY, Pecina JL, Upatising B, et al. A randomized controlled trial of telemonitoring in older adults with multiple health issues to prevent hospitalizations and emergency department visits. *Arch Intern Med.* 2012;172(10):773-779.
35. Voils CI, Coffman CJ, Yancy WS, Jr., et al. A randomized controlled trial to evaluate the effectiveness of CouPLES: a spouse-assisted lifestyle change intervention to improve low-density lipoprotein cholesterol. *Prev Med.* 2013;56(1):46-52.
36. Harfterkamp M, van de Loo-Neus G, Minderaa RB, et al. A randomized double-blind study of atomoxetine versus placebo for attention-deficit/hyperactivity disorder symptoms in children with autism spectrum disorder. *J Am Acad Child Adolesc Psychiatry.* 2012;51(7):733-741.
37. Smorenburg CH, de Groot SM, van Leeuwen-Stok AE, et al. A randomized phase III study comparing pegylated liposomal doxorubicin with capecitabine as first-line chemotherapy in elderly patients with metastatic breast cancer: results of the OMEGA study of the Dutch Breast Cancer Research Group BOOG. *Ann Oncol.* 2014;25(3):599-605.
38. Van Buren G, 2nd, Bloomston M, Hughes SJ, et al. A randomized prospective multicenter trial of pancreaticoduodenectomy with and without routine intraperitoneal drainage. *Ann Surg.* 2014;259(4):605-612.
39. Naselli A, Introini C, Timossi L, et al. A randomized prospective trial to assess the impact of transurethral resection in narrow band imaging modality on non-muscle-invasive bladder cancer recurrence. *Eur Urol.* 2012;61(5):908-913.
40. Lynde CW, Gupta AK, Guenther L, Poulin Y, Levesque A, Bissonnette R. A randomized study comparing the combination of nbUVB and etanercept to etanercept monotherapy in patients with psoriasis who do not exhibit an excellent response after 12 weeks of etanercept. *J Dermatolog Treat.* 2012;23(4):261-267.
41. Rossetti AO, Milligan TA, Vulliemoz S, Michaelides C, Bertschi M, Lee JW. A randomized trial for the treatment of refractory status epilepticus. *Neurocrit Care.* 2011;14(1):4-10.
42. Imazio M, Brucato A, Cemin R, et al. A randomized trial of colchicine for acute pericarditis. *N Engl J Med.* 2013;369(16):1522-1528.
43. Slusher TM, Olusanya BO, Vreman HJ, et al. A Randomized Trial of Phototherapy with Filtered Sunlight in African Neonates. *N Engl J Med.* 2015;373(12):1115-1124.
44. Low N, Bavdekar A, Jeyaseelan L, et al. A randomized, controlled trial of an aerosolized vaccine against measles. *N Engl J Med.* 2015;372(16):1519-1529.
45. Berntsen DA, Sinnott LT, Mutti DO, Zadnik K. A randomized trial using progressive addition lenses to evaluate theories of myopia progression in children with a high lag of accommodation. *Invest Ophthalmol Vis Sci.* 2012;53(2):640-649.
46. Katagiri H, Fujikoshi S, Suzuki T, et al. A randomized, double-blind, placebo-controlled study of rapid-acting intramuscular olanzapine in Japanese patients for schizophrenia with acute agitation. *BMC Psychiatry.* 2013;13:20.

© 2019 Chen T et al. *JAMA Network Open*.

47. Borges JL, Bilezikian JP, Jones-Leone AR, et al. A randomized, parallel group, double-blind, multicentre study comparing the efficacy and safety of Avandamet (rosiglitazone/metformin) and metformin on long-term glycaemic control and bone mineral density after 80 weeks of treatment in drug-naive type 2 diabetes mellitus patients. *Diabetes Obes Metab.* 2011;13(11):1036-1046.
48. Mitchell KE, Johnson-Warrington V, Apps LD, et al. A self-management programme for COPD: a randomised controlled trial. *Eur Respir J.* 2014;44(6):1538-1547.
49. Tan J, Humphrey S, Vender R, et al. A treatment for severe nodular acne: a randomized investigator-blinded, controlled, noninferiority trial comparing fixed-dose adapalene/benzoyl peroxide plus doxycycline vs. oral isotretinoin. *Br J Dermatol.* 2014;171(6):1508-1516.
50. Mease P, Genovese MC, Gladstein G, et al. Abatacept in the treatment of patients with psoriatic arthritis: results of a six-month, multicenter, randomized, double-blind, placebo-controlled, phase II trial. *Arthritis Rheum.* 2011;63(4):939-948.
51. Siddiqi K, Khan A, Ahmad M, et al. Action to stop smoking in suspected tuberculosis (ASSIST) in Pakistan: a cluster randomized, controlled trial. *Ann Intern Med.* 2013;158(9):667-675.
52. Brouwer WP, Xie Q, Sonneveld MJ, et al. Adding pegylated interferon to entecavir for hepatitis B e antigen-positive chronic hepatitis B: A multicenter randomized trial (ARES study). *Hepatology.* 2015;61(5):1512-1522.
53. Kruidenier LM, Nicolai SP, Rouwet EV, Peters RJ, Prins MH, Teijink JA. Additional supervised exercise therapy after a percutaneous vascular intervention for peripheral arterial disease: a randomized clinical trial. *J Vasc Interv Radiol.* 2011;22(7):961-968.
54. Vansteenkiste J, Zielinski M, Linder A, et al. Adjuvant MAGE-A3 immunotherapy in resected non-small-cell lung cancer: phase II randomized study results. *J Clin Oncol.* 2013;31(19):2396-2403.
55. Bruix J, Takayama T, Mazzaferro V, et al. Adjuvant sorafenib for hepatocellular carcinoma after resection or ablation (STORM): a phase 3, randomised, double-blind, placebo-controlled trial. *The Lancet Oncology.* 2015;16(13):1344-1354.
56. Watson JM, Crosby H, Dale VM, et al. AESOPS: a randomised controlled trial of the clinical effectiveness and cost-effectiveness of opportunistic screening and stepped care interventions for older hazardous alcohol users in primary care. *Health Technol Assess.* 2013;17(25):1-158.
57. Aliti GB, Rabelo ER, Clausell N, Rohde LE, Biolo A, Beck-da-Silva L. Aggressive fluid and sodium restriction in acute decompensated heart failure: a randomized clinical trial. *JAMA Intern Med.* 2013;173(12):1058-1064.
58. Higgins P, Walters MR, Murray HM, et al. Allopurinol reduces brachial and central blood pressure, and carotid intima-media thickness progression after ischaemic stroke and transient ischaemic attack: a randomised controlled trial. *Heart.* 2014;100(14):1085-1092.
59. Parker C, Nilsson S, Heinrich D, et al. Alpha emitter radium-223 and survival in metastatic prostate cancer. *N Engl J Med.* 2013;369(3):213-223.
60. McMurray JJ, Packer M, Desai AS, et al. Angiotensin-neprilysin inhibition versus enalapril in heart failure. *N Engl J Med.* 2014;371(11):993-1004.
61. Connolly SJ, Eikelboom J, Joyner C, et al. Apixaban in patients with atrial fibrillation. *N Engl J Med.* 2011;364(9):806-817.
62. Burt RK, Shah SJ, Dill K, et al. Autologous non-myeloablative haemopoietic stem-cell transplantation compared with pulse cyclophosphamide once per month for systemic sclerosis (ASSIST): an open-label, randomised phase 2 trial. *The Lancet.* 2011;378(9790):498-506.
63. Kojima T, Miyauchi K, Yokoyama T, et al. Azelnidipine and Amlodipine Anti-Coronary Atherosclerosis Trial in Hypertensive Patients Undergoing Coronary Intervention by Serial Volumetric Intravascular Ultrasound Analysis in Juntendo University (ALPS-J). *Circulation Journal.* 2011;75(5):1071-1079.

© 2019 Chen T et al. *JAMA Network Open*.

64. Albert RK, Connett J, Bailey WC, et al. Azithromycin for prevention of exacerbations of COPD. *N Engl J Med.* 2011;365(8):689-698.
65. Papi A, Corradi M, Pigeon-Francisco C, et al. Beclometasone-formoterol as maintenance and reliever treatment in patients with asthma: a double-blind, randomised controlled trial. *Lancet Respir Med.* 2013;1(1):23-31.
66. Hanania NA, Crater GD, Morris AN, Emmett AH, O'Dell DM, Niewoehner DE. Benefits of adding fluticasone propionate/salmeterol to tiotropium in moderate to severe COPD. *Respir Med.* 2012;106(1):91-101.
67. Foley JE, Bunck MC, Moller-Goede DL, et al. Beta cell function following 1 year vildagliptin or placebo treatment and after 12 week washout in drug-naive patients with type 2 diabetes and mild hyperglycaemia: a randomised controlled trial. *Diabetologia.* 2011;54(8):1985-1991.
68. Elgadi MM, Piliero PJ. Boosted tipranavir versus darunavir in treatment-experienced patients: observational data from the randomized POTENT trial. *Drugs R D.* 2011;11(4):295-302.
69. Corte TJ, Keir GJ, Dimopoulos K, et al. Bosentan in pulmonary hypertension associated with fibrotic idiopathic interstitial pneumonia. *Am J Respir Crit Care Med.* 2014;190(2):208-217.
70. Lawrance IC, Willert RP, Murray K. Bowel cleansing for colonoscopy: prospective randomized assessment of efficacy and of induced mucosal abnormality with three preparation agents. *Endoscopy.* 2011;43(5):412-418.
71. Coleman RE, Marshall H, Cameron D, et al. Breast-cancer adjuvant therapy with zoledronic acid. *N Engl J Med.* 2011;365(15):1396-1405.
72. Kunkler IH, Williams LJ, Jack WJL, Cameron DA, Dixon JM. Breast-conserving surgery with or without irradiation in women aged 65 years or older with early breast cancer (PRIME II): a randomised controlled trial. *The Lancet Oncology.* 2015;16(3):266-273.
73. Husain N, Afsar S, Ara J, et al. Brief psychological intervention after self-harm: randomised controlled trial from Pakistan. *Br J Psychiatry.* 2014;204(6):462-470.
74. Gelderblom H, Blay JY, Seddon BM, et al. Brostallicin versus doxorubicin as first-line chemotherapy in patients with advanced or metastatic soft tissue sarcoma: an European Organisation for Research and Treatment of Cancer Soft Tissue and Bone Sarcoma Group randomised phase II and pharmacogenetic study. *Eur J Cancer.* 2014;50(2):388-396.
75. Shin WG, Kim SJ, Choi MH, et al. Can rebamipide and proton pump inhibitor combination therapy promote the healing of endoscopic submucosal dissection-induced ulcers? A randomized, prospective, multicenter study. *Gastrointest Endosc.* 2012;75(4):739-747.
76. Yohannan J, Munoz B, Mkocha H, et al. Can we stop mass drug administration prior to 3 annual rounds in communities with low prevalence of trachoma?: PRET Ziada trial results. *JAMA Ophthalmol.* 2013;131(4):431-436.
77. Mogul HR, Freeman R, Nguyen K, et al. Carbohydrate modified diet & insulin sensitizers reduce body weight & modulate metabolic syndrome measures in EMPOWIR (enhance the metabolic profile of women with insulin resistance): a randomized trial of normoglycemic women with midlife weight gain. *PLoS One.* 2014;9(9):e108264.
78. Quoix E, Zalcman G, Oster J-P, et al. Carboplatin and weekly paclitaxel doublet chemotherapy compared with monotherapy in elderly patients with advanced non-small-cell lung cancer: IFCT-0501 randomised, phase 3 trial. *The Lancet.* 2011;378(9796):1079-1088.
79. Zwar NA, Hermiz O, Comino E, et al. Care of patients with a diagnosis of chronic obstructive pulmonary disease: a cluster randomised controlled trial. *Med J Aust.* 2012;197(7):394-398.

© 2019 Chen T et al. *JAMA Network Open*.

80. Costa JA, Menier M, Doran TJ, Kohler TS. Catheter length preference in wheelchair-using men who perform routine clean intermittent catheterization. *Spinal Cord.* 2013;51(10):772-775.
81. James ND, Sydes MR, Mason MD, et al. Celecoxib plus hormone therapy versus hormone therapy alone for hormone-sensitive prostate cancer: first results from the STAMPEDE multiarm, multistage, randomised controlled trial. *The Lancet Oncology.* 2012;13(5):549-558.
82. Wang IJ, Wang JY. Children with atopic dermatitis show clinical improvement after Lactobacillus exposure. *Clin Exp Allergy.* 2015;45(4):779-787.
83. Kean RJ, Lamport DJ, Dodd GF, et al. Chronic consumption of flavanone-rich orange juice is associated with cognitive benefits: an 8-wk, randomized, double-blind, placebo-controlled trial in healthy older adults. *Am J Clin Nutr.* 2015;101(3):506-514.
84. Brand PL, Luz Garcia-Garcia M, Morison A, Vermeulen JH, Weber HC. Ciclesonide in wheezy preschool children with a positive asthma predictive index or atopy. *Respir Med.* 2011;105(11):1588-1595.
85. Ryan D, Price D, Musgrave SD, et al. Clinical and cost effectiveness of mobile phone supported self monitoring of asthma: multicentre randomised controlled trial. *BMJ.* 2012;344:e1756.
86. Wessel DL, Berger F, Li JS, et al. Clopidogrel in infants with systemic-to-pulmonary-artery shunts. *N Engl J Med.* 2013;368(25):2377-2384.
87. Forster A, Young J, Chapman K, et al. Cluster Randomized Controlled Trial: Clinical and Cost-Effectiveness of a System of Longer-Term Stroke Care. *Stroke.* 2015;46(8):2212-2219.
88. Houston TK, Delaughter KL, Ray MN, et al. Cluster-randomized trial of a web-assisted tobacco quality improvement intervention of subsequent patient tobacco product use: a National Dental PBRN study. *BMC Oral Health.* 2013;13:13.
89. Wood JJ, Ehrenreich-May J, Alessandri M, et al. Cognitive behavioral therapy for early adolescents with autism spectrum disorders and clinical anxiety: a randomized, controlled trial. *Behav Ther.* 2015;46(1):7-19.
90. Ramon JM, Morchon S, Baena A, Masuet-Aumatell C. Combining varenicline and nicotine patches: a randomized controlled trial study in smoking cessation. *BMC Med.* 2014;12:172.
91. Harmsen CG, Kristiansen IS, Larsen PV, et al. Communicating risk using absolute risk reduction or prolongation of life formats: cluster-randomised trial in general practice. *Br J Gen Pract.* 2014;64(621):e199-207.
92. Rini BI, Escudier B, Tomczak P, et al. Comparative effectiveness of axitinib versus sorafenib in advanced renal cell carcinoma (AXIS): a randomised phase 3 trial. *The Lancet.* 2011;378(9807):1931-1939.
93. Ostovaneh MR, Saeidi B, Hajifathalian K, et al. Comparing omeprazole with fluoxetine for treatment of patients with heartburn and normal endoscopy who failed once daily proton pump inhibitors: double-blind placebo-controlled trial. *Neurogastroenterol Motil.* 2014;26(5):670-678.
94. Tehraninejad ES, Hafezi M, Arabipoor A, Aziminekoo E, Chehrazi M, Bahmanabadi A. Comparison of cabergoline and intravenous albumin in the prevention of ovarian hyperstimulation syndrome: a randomized clinical trial. *J Assist Reprod Genet.* 2012;29(3):259-264.
95. Hilton TJ, Ferracane JL, Mancl L, Northwest Practice-based Research Collaborative in Evidence-based D. Comparison of CaOH with MTA for direct pulp capping: a PBRN randomized clinical trial. *J Dent Res.* 2013;92(7 Suppl):16S-22S.
96. Allen RP, Chen C, Garcia-Borreguero D, et al. Comparison of pregabalin with pramipexole for restless legs syndrome. *N Engl J Med.* 2014;370(7):621-631.

© 2019 Chen T et al. *JAMA Network Open*.

97. Zhang H, Zhang K, Zhang X, et al. Comparison of two hyaluronic acid formulations for safety and efficacy (CHASE) study in knee osteoarthritis: a multicenter, randomized, double-blind, 26-week non-inferiority trial comparing Durolane to Artz. *Arthritis Res Ther.* 2015;17:51.
98. Prestmo A, Hagen G, Sletvold O, et al. Comprehensive geriatric care for patients with hip fractures: a prospective, randomised, controlled trial. *The Lancet.* 2015;385(9978):1623-1633.
99. Dmitrovic R, Kunselman AR, Legro RS. Continuous compared with cyclic oral contraceptives for the treatment of primary dysmenorrhea: a randomized controlled trial. *Obstet Gynecol.* 2012;119(6):1143-1150.
100. Preti RC, Ramirez LM, Monteiro ML, Carra MK, Pelayes DE, Takahashi WY. Contrast sensitivity evaluation in high risk proliferative diabetic retinopathy treated with panretinal photocoagulation associated or not with intravitreal bevacizumab injections: a randomised clinical trial. *Br J Ophthalmol.* 2013;97(7):885-889.
101. Morgan GJ, Davies FE, Gregory WM, et al. Cyclophosphamide, thalidomide, and dexamethasone (CTD) as initial therapy for patients with multiple myeloma unsuitable for autologous transplantation. *Blood.* 2011;118(5):1231-1238.
102. Huttner B, Haustein T, Uckay I, et al. Decolonization of intestinal carriage of extended-spectrum beta-lactamase-producing Enterobacteriaceae with oral colistin and neomycin: a randomized, double-blind, placebo-controlled trial. *J Antimicrob Chemother.* 2013;68(10):2375-2382.
103. Hughes TP, Lipton JH, Spector N, et al. Deep molecular responses achieved in patients with CML-CP who are switched to nilotinib after long-term imatinib. *Blood.* 2014;124(5):729-736.
104. Corbacioglu S, Cesaro S, Faraci M, et al. Defibrotide for prophylaxis of hepatic veno-occlusive disease in paediatric haemopoietic stem-cell transplantation: an open-label, phase 3, randomised controlled trial. *The Lancet.* 2012;379(9823):1301-1309.
105. Hsu CH, Liao YL, Lin SC, Tsai TH, Huang CJ, Chou P. Does supplementation with green tea extract improve insulin resistance in obese type 2 diabetics? A randomized, double-blind, and placebo-controlled clinical trial. *Altern Med Rev.* 2011;16(2):157-163.
106. Prieto-Jimenez CA, Cardenas VM, Fischbach LA, et al. Double-blind randomized trial of quadruple sequential Helicobacter pylori eradication therapy in asymptomatic infected children in El Paso, Texas. *J Pediatr Gastroenterol Nutr.* 2011;52(3):319-325.
107. Mezhebovsky I, Magi K, She F, Datto C, Eriksson H. Double-blind, randomized study of extended release quetiapine fumarate (quetiapine XR) monotherapy in older patients with generalized anxiety disorder. *Int J Geriatr Psychiatry.* 2013;28(6):615-625.
108. Judson I, Verweij J, Gelderblom H, et al. Doxorubicin alone versus intensified doxorubicin plus ifosfamide for first-line treatment of advanced or metastatic soft-tissue sarcoma: a randomised controlled phase 3 trial. *The Lancet Oncology.* 2014;15(4):415-423.
109. Trouillet JL, Luyt CE, Guiguet M, et al. Early percutaneous tracheotomy versus prolonged intubation of mechanically ventilated patients after cardiac surgery: a randomized trial. *Ann Intern Med.* 2011;154(6):373-383.
110. Blakely ML, Williams R, Dassinger MS, et al. Early vs interval appendectomy for children with perforated appendicitis. *Arch Surg.* 2011;146(6):660-665.
111. Giugliano RP, Ruff CT, Braunwald E, et al. Edoxaban versus warfarin in patients with atrial fibrillation. *N Engl J Med.* 2013;369(22):2093-2104.
112. Coburn KD, Marcantonio S, Lazansky R, Keller M, Davis N. Effect of a community-based nursing intervention on mortality in chronically ill older adults: a randomized controlled trial. *PLoS Med.* 2012;9(7):e1001265.

© 2019 Chen T et al. *JAMA Network Open*.

113. Griffin SJ, Borch-Johnsen K, Davies MJ, et al. Effect of early intensive multifactorial therapy on 5-year cardiovascular outcomes in individuals with type 2 diabetes detected by screening (ADDITION-Europe): a cluster-randomised trial. *The Lancet.* 2011;378(9786):156-167.
114. Salam RA, Darmstadt GL, Bhutta ZA. Effect of emollient therapy on clinical outcomes in preterm neonates in Pakistan: a randomised controlled trial. *Arch Dis Child Fetal Neonatal Ed.* 2015;100(3):F210-215.
115. Sureshkumar KK, Hussain SM, Ko TY, Thai NL, Marcus RJ. Effect of high-dose erythropoietin on graft function after kidney transplantation: a randomized, double-blind clinical trial. *Clin J Am Soc Nephrol.* 2012;7(9):1498-1506.
116. Boisson S, Stevenson M, Shapiro L, et al. Effect of household-based drinking water chlorination on diarrhoea among children under five in Orissa, India: a double-blind randomised placebo-controlled trial. *PLoS Med.* 2013;10(8):e1001497.
117. Devoogdt N, Christiaens MR, Geraerts I, et al. Effect of manual lymph drainage in addition to guidelines and exercise therapy on arm lymphoedema related to breast cancer: randomised controlled trial. *BMJ.* 2011;343:d5326.
118. Reignier J, Mercier E, Le Gouge A, et al. Effect of not monitoring residual gastric volume on risk of ventilator-associated pneumonia in adults receiving mechanical ventilation and early enteral feeding: a randomized controlled trial. *JAMA.* 2013;309(3):249-256.
119. Wiegersma M, Panman CM, Kollen BJ, Berger MY, Lisman-Van Leeuwen Y, Dekker JH. Effect of pelvic floor muscle training compared with watchful waiting in older women with symptomatic mild pelvic organ prolapse: randomised controlled trial in primary care. *BMJ.* 2014;349:g7378.
120. Redfield MM, Chen HH, Borlaug BA, et al. Effect of phosphodiesterase-5 inhibition on exercise capacity and clinical status in heart failure with preserved ejection fraction: a randomized clinical trial. *JAMA.* 2013;309(12):1268-1277.
121. Bennell KL, Egerton T, Martin J, et al. Effect of physical therapy on pain and function in patients with hip osteoarthritis: a randomized clinical trial. *JAMA.* 2014;311(19):1987-1997.
122. Eftekhar M, Rahsepar M, Rahmani E. Effect of progesterone supplementation on natural frozen-thawed embryo transfer cycles: a randomized controlled trial. *Int J Fertil Steril.* 2013;7(1):13-20.
123. Thirumurthy H, Masters SH, Rao S, et al. Effect of providing conditional economic compensation on uptake of voluntary medical male circumcision in Kenya: a randomized clinical trial. *JAMA.* 2014;312(7):703-711.
124. Ikramuddin S, Blackstone RP, Brancatisano A, et al. Effect of reversible intermittent intra-abdominal vagal nerve blockade on morbid obesity: the ReCharge randomized clinical trial. *JAMA.* 2014;312(9):915-922.
125. Witham MD, Adams F, Kabir G, Kennedy G, Belch JJ, Khan F. Effect of short-term vitamin D supplementation on markers of vascular health in South Asian women living in the UK--a randomised controlled trial. *Atherosclerosis.* 2013;230(2):293-299.
126. Pinggera GM, Frauscher F, Paduch DA, et al. Effect of tadalafil once daily on prostate blood flow and perfusion in men with lower urinary tract symptoms secondary to benign prostatic hyperplasia: a randomized, double-blind, multicenter, placebo-controlled trial. *Urology.* 2014;84(2):412-419.
127. Nicholls SJ, Ballantyne CM, Barter PJ, et al. Effect of two intensive statin regimens on progression of coronary disease. *N Engl J Med.* 2011;365(22):2078-2087.
128. Gheorghiade M, Greene SJ, Butler J, et al. Effect of Vericiguat, a Soluble Guanylate Cyclase Stimulator, on Natriuretic Peptide Levels in Patients With Worsening Chronic Heart Failure and Reduced Ejection Fraction: The SOCRATES-REDUCED Randomized Trial. *JAMA.* 2015;314(21):2251-2262.

© 2019 Chen T et al. *JAMA Network Open*.

129. Marteau TM, Aveyard P, Munafo MR, et al. Effect on adherence to nicotine replacement therapy of informing smokers their dose is determined by their genotype: a randomised controlled trial. *PLoS One.* 2012;7(4):e35249.
130. Laing BY, Mangione CM, Tseng CH, et al. Effectiveness of a smartphone application for weight loss compared with usual care in overweight primary care patients: a randomized, controlled trial. *Ann Intern Med.* 2014;161(10 Suppl):S5-12.
131. Coffeng JK, Boot CR, Duijts SF, Twisk JW, van Mechelen W, Hendriksen IJ. Effectiveness of a worksite social & physical environment intervention on need for recovery, physical activity and relaxation; results of a randomized controlled trial. *PLoS One.* 2014;9(12):e114860.
132. Barkun AN, Bhat M, Armstrong D, et al. Effectiveness of disseminating consensus management recommendations for ulcer bleeding: a cluster randomized trial. *CMAJ.* 2013;185(3):E156-166.
133. Zanger P, Nurjadi D, Gabor J, Gaile M, Kremsner PG. Effectiveness of rifaximin in prevention of diarrhoea in individuals travelling to south and southeast Asia: a randomised, double-blind, placebo-controlled, phase 3 trial. *The Lancet Infectious Diseases.* 2013;13(11):946-954.
134. Shorr RI, Chandler AM, Mion LC, et al. Effects of an intervention to increase bed alarm use to prevent falls in hospitalized patients: a cluster randomized trial. *Ann Intern Med.* 2012;157(10):692-699.
135. Grinsztejn B, Hosseinipour MC, Ribaudo HJ, et al. Effects of early versus delayed initiation of antiretroviral treatment on clinical outcomes of HIV-1 infection: results from the phase 3 HPTN 052 randomised controlled trial. *The Lancet Infectious Diseases.* 2014;14(4):281-290.
136. Bolliger CT, Issa JS, Posadas-Valay R, et al. Effects of varenicline in adult smokers: a multinational, 24-week, randomized, double-blind, placebo-controlled study. *Clin Ther.* 2011;33(4):465-477.
137. Sieper J, van der Heijde D, Dougados M, et al. Efficacy and safety of adalimumab in patients with non-radiographic axial spondyloarthritis: results of a randomised placebo-controlled trial (ABILITY-1). *Ann Rheum Dis.* 2013;72(6):815-822.
138. Ishigooka J, Nakamura J, Fujii Y, et al. Efficacy and safety of aripiprazole once-monthly in Asian patients with schizophrenia: a multicenter, randomized, double-blind, non-inferiority study versus oral aripiprazole. *Schizophr Res.* 2015;161(2-3):421-428.
139. Whittaker S, Ortiz P, Dummer R, et al. Efficacy and safety of bexarotene combined with psoralen-ultraviolet A (PUVA) compared with PUVA treatment alone in stage IB-IIA mycosis fungoides: final results from the EORTC Cutaneous Lymphoma Task Force phase III randomized clinical trial (NCT00056056). *Br J Dermatol.* 2012;167(3):678-687.
140. Vazquez JA, Gonzalez Patzan LD, Stricklin D, et al. Efficacy and safety of ceftazidime-avibactam versus imipenem-cilastatin in the treatment of complicated urinary tract infections, including acute pyelonephritis, in hospitalized adults: results of a prospective, investigator-blinded, randomized study. *Curr Med Res Opin.* 2012;28(12):1921-1931.
141. Girerd X, Rosenbaum D, Aoun J. Efficacy and safety of early versus late titration of fixed-dose irbesartan/hydrochlorothiazide: ACTUAL study. *Blood Pressure.* 2011;20(sup2):22-29.
142. Woodcock A, Bleecker ER, Lotvall J, et al. Efficacy and safety of fluticasone furoate/vilanterol compared with fluticasone propionate/salmeterol combination in adult and adolescent patients with persistent asthma: a randomized trial. *Chest.* 2013;144(4):1222-1229.
143. Sieper J, Lenaerts J, Wollenhaupt J, et al. Efficacy and safety of infliximab plus naproxen versus naproxen alone in patients with early, active axial spondyloarthritis: results from the double-blind, placebo-controlled INFAST study, Part 1. *Ann Rheum Dis.* 2014;73(1):101-107.

© 2019 Chen T et al. *JAMA Network Open*.

144. Buhl R, Gessner C, Schuermann W, et al. Efficacy and safety of once-daily QVA149 compared with the free combination of once-daily tiotropium plus twice-daily formoterol in patients with moderate-to-severe COPD (QUANTIFY): a randomised, non-inferiority study. *Thorax.* 2015;70(4):311-319.
145. Fonseca V, Staels B, Morgan JD, 2nd, et al. Efficacy and safety of sitagliptin added to ongoing metformin and pioglitazone combination therapy in a randomized, placebo-controlled, 26-week trial in patients with type 2 diabetes. *J Diabetes Complications.* 2013;27(2):177-183.
146. Altenberger J, Parissis JT, Costard-Jaeckle A, et al. Efficacy and safety of the pulsed infusions of levosimendan in outpatients with advanced heart failure (LevoRep) study: a multicentre randomized trial. *Eur J Heart Fail.* 2014;16(8):898-906.
147. Jones PW, Singh D, Bateman ED, et al. Efficacy and safety of twice-daily aclidinium bromide in COPD patients: the ATTAIN study. *Eur Respir J.* 2012;40(4):830-836.
148. Charbonnel B, Steinberg H, Eymard E, et al. Efficacy and safety over 26 weeks of an oral treatment strategy including sitagliptin compared with an injectable treatment strategy with liraglutide in patients with type 2 diabetes mellitus inadequately controlled on metformin: a randomised clinical trial. *Diabetologia.* 2013;56(7):1503-1511.
149. Fuhr R, Magnussen H, Sarem K, et al. Efficacy of aclidinium bromide 400 mug twice daily compared with placebo and tiotropium in patients with moderate to severe COPD. *Chest.* 2012;141(3):745-752.
150. Braga TD, da Silva GA, de Lira PI, de Carvalho Lima M. Efficacy of Bifidobacterium breve and Lactobacillus casei oral supplementation on necrotizing enterocolitis in very-low-birth-weight preterm infants: a double-blind, randomized, controlled trial. *Am J Clin Nutr.* 2011;93(1):81-86.
151. Papas AS, Vollmer WM, Gullion CM, et al. Efficacy of chlorhexidine varnish for the prevention of adult caries: a randomized trial. *J Dent Res.* 2012;91(2):150-155.
152. Barrett PN, Berezuk G, Fritsch S, et al. Efficacy, safety, and immunogenicity of a Vero-cell-culture-derived trivalent influenza vaccine: a multicentre, double-blind, randomised, placebo-controlled trial. *The Lancet.* 2011;377(9767):751-759.
153. Beer TM, Armstrong AJ, Rathkopf DE, et al. Enzalutamide in metastatic prostate cancer before chemotherapy. *N Engl J Med.* 2014;371(5):424-433.
154. Jouve P, Bazin JE, Petit A, et al. Epidural versus continuous preperitoneal analgesia during fast-track open colorectal surgery: a randomized controlled trial. *Anesthesiology.* 2013;118(3):622-630.
155. Songprakun W, McCann TV. Evaluation of a cognitive behavioural self-help manual for reducing depression: a randomized controlled trial. *J Psychiatr Ment Health Nurs.* 2012;19(7):647-653.
156. Shah GR, Chaudhari MV, Patankar SB, Pensalwar SV, Sabale VP, Sonawane NA. Evaluation of a multi-herb supplement for erectile dysfunction: a randomized double-blind, placebo-controlled study. *BMC Complement Altern Med.* 2012;12:155.
157. Kaine J, Gladstein G, Strusberg I, et al. Evaluation of abatacept administered subcutaneously in adults with active rheumatoid arthritis: impact of withdrawal and reintroduction on immunogenicity, efficacy and safety (phase Iiib ALLOW study). *Ann Rheum Dis.* 2012;71(1):38-44.
158. Orwoll ES, Shapiro J, Veith S, et al. Evaluation of teriparatide treatment in adults with osteogenesis imperfecta. *J Clin Invest.* 2014;124(2):491-498.
159. Domar AD, Gross J, Rooney K, Boivin J. Exploratory randomized trial on the effect of a brief psychological intervention on emotions, quality of life, discontinuation, and pregnancy rates in in vitro fertilization patients. *Fertil Steril.* 2015;104(2):440-451 e447.

© 2019 Chen T et al. *JAMA Network Open*.

160. Morton DL, Thompson JF, Cochran AJ, et al. Final trial report of sentinel-node biopsy versus nodal observation in melanoma. *N Engl J Med.* 2014;370(7):599-609.
161. Poulsen SK, Due A, Jordy AB, et al. Health effect of the New Nordic Diet in adults with increased waist circumference: a 6-mo randomized controlled trial. *Am J Clin Nutr.* 2014;99(1):35-45.
162. Chellamuthu P, Sharma S, Jain P, Kaushik JS, Seth A, Aneja S. High dose (4 mg/kg/day) versus usual dose (2 mg/kg/day) oral prednisolone for treatment of infantile spasms: an open-label, randomized controlled trial. *Epilepsy Res.* 2014;108(8):1378-1384.
163. Asfar P, Meziani F, Hamel JF, et al. High versus low blood-pressure target in patients with septic shock. *N Engl J Med.* 2014;370(17):1583-1593.
164. Nagata N, Niikura R, Shimbo T, et al. High-dose barium impaction therapy for the recurrence of colonic diverticular bleeding: a randomized controlled trial. *Ann Surg.* 2015;261(2):269-275.
165. Tiplady S, Jones G, Campbell M, Johnson S, Ledger W. Home ovulation tests and stress in women trying to conceive: a randomized controlled trial. *Hum Reprod.* 2013;28(1):138-151.
166. Teut M, Dahler J, Hirschberg U, Luedtke R, Albrecht H, Witt CM. Homeopathic drug proving of Okoubaka aubrevillei: a randomised placebo-controlled trial. *Trials.* 2013;14:96.
167. Bingham CO, 3rd, Rizzo W, Kivitz A, Hassanali A, Upmanyu R, Klearman M. Humoral immune response to vaccines in patients with rheumatoid arthritis treated with tocilizumab: results of a randomised controlled trial (VISARA). *Ann Rheum Dis.* 2015;74(5):818-822.
168. Hausleiter J, Meyer TS, Martuscelli E, et al. Image quality and radiation exposure with prospectively ECG-triggered axial scanning for coronary CT angiography: the multicenter, multivendor, randomized PROTECTION-III study. *JACC Cardiovasc Imaging.* 2012;5(5):484-493.
169. Zhu F-C, Liang Z-L, Li X-L, et al. Immunogenicity and safety of an enterovirus 71 vaccine in healthy Chinese children and infants: a randomised, double-blind, placebo-controlled phase 2 clinical trial. *The Lancet.* 2013;381(9871):1037-1045.
170. Nash P, Nayiager S, Genovese MC, et al. Immunogenicity, safety, and efficacy of abatacept administered subcutaneously with or without background methotrexate in patients with rheumatoid arthritis: results from a phase III, international, multicenter, parallel-arm, open-label study. *Arthritis Care Res (Hoboken).* 2013;65(5):718-728.
171. Howman A, Chapman TL, Langdon MM, et al. Immunosuppression for progressive membranous nephropathy: a UK randomised controlled trial. *The Lancet.* 2013;381(9868):744-751.
172. Dugravier R, Tubach F, Saias T, et al. Impact of a manualized multifocal perinatal home-visiting program using psychologists on postnatal depression: the CAPEDP randomized controlled trial. *PLoS One.* 2013;8(8):e72216.
173. Lau AY, Sintchenko V, Crimmins J, Magrabi F, Gallego B, Coiera E. Impact of a web-based personally controlled health management system on influenza vaccination and health services utilization rates: a randomized controlled trial. *J Am Med Inform Assoc.* 2012;19(5):719-727.
174. Cohn SM, McCarthy J, Stewart RM, Jonas RB, Dent DL, Michalek JE. Impact of low-dose vasopressin on trauma outcome: prospective randomized study. *World J Surg.* 2011;35(2):430-439.
175. Ganguly S, Saha P, Guha SK, et al. In vivo therapeutic efficacy of chloroquine alone or in combination with primaquine against vivax malaria in Kolkata, West Bengal, India, and polymorphism in pvmdr1 and pvcrt-o genes. *Antimicrob Agents Chemother.* 2013;57(3):1246-1251.

© 2019 Chen T et al. *JAMA Network Open*.

176. Walsh TS, Salisbury LG, Merriweather JL, et al. Increased Hospital-Based Physical Rehabilitation and Information Provision After Intensive Care Unit Discharge: The RECOVER Randomized Clinical Trial. *JAMA Intern Med.* 2015;175(6):901-910.
177. Hajek P, McRobbie H, Myers Smith K, Phillips A, Cornwall D, Dhanji AR. Increasing varenicline dose in smokers who do not respond to the standard dosage: a randomized clinical trial. *JAMA Intern Med.* 2015;175(2):266-271.
178. Mourvillier B, Tubach F, van de Beek D, et al. Induced hypothermia in severe bacterial meningitis: a randomized clinical trial. *JAMA.* 2013;310(20):2174-2183.
179. Detert J, Bastian H, Listing J, et al. Induction therapy with adalimumab plus methotrexate for 24 weeks followed by methotrexate monotherapy up to week 48 versus methotrexate therapy alone for DMARD-naive patients with early rheumatoid arthritis: HIT HARD, an investigator-initiated study. *Ann Rheum Dis.* 2013;72(6):844-850.
180. Ratner R, Wynne A, Nakhle S, Brusco O, Vlajnic A, Rendell M. Influence of preprandial vs. postprandial insulin glulisine on weight and glycaemic control in patients initiating basal-bolus regimen for type 2 diabetes: a multicenter, randomized, parallel, open-label study (NCT00135096). *Diabetes Obes Metab.* 2011;13(12):1142-1148.
181. Bilton D, Robinson P, Cooper P, et al. Inhaled dry powder mannitol in cystic fibrosis: an efficacy and safety study. *Eur Respir J.* 2011;38(5):1071-1080.
182. Vance G, Janowitz B, Chen M, et al. Integrating family planning messages into immunization services: a cluster-randomized trial in Ghana and Zambia. *Health Policy Plan.* 2014;29(3):359-366.
183. Thiele H, Wöhrle J, Hambrecht R, et al. Intracoronary versus intravenous bolus abciximab during primary percutaneous coronary intervention in patients with acute ST-elevation myocardial infarction: a randomised trial. *The Lancet.* 2012;379(9819):923-931.
184. Nickel JC, Crossland A, Davis E, et al. Investigation of a Ca2+ channel alpha2delta ligand for the treatment of interstitial cystitis: results of a randomized, double-blind, placebo controlled phase II trial. *J Urol.* 2012;188(3):817-823.
185. Hodi FS, Lee S, McDermott DF, et al. Ipilimumab plus sargramostim vs ipilimumab alone for treatment of metastatic melanoma: a randomized clinical trial. *JAMA.* 2014;312(17):1744-1753.
186. Harrison C, Kiladjian JJ, Al-Ali HK, et al. JAK inhibition with ruxolitinib versus best available therapy for myelofibrosis. *N Engl J Med.* 2012;366(9):787-798.
187. Kristeller JL, Zavorsky GS, Prior JE, et al. Lack of effectiveness of sodium bicarbonate in preventing kidney injury in patients undergoing cardiac surgery: a randomized controlled trial. *Pharmacotherapy.* 2013;33(7):710-717.
188. Writing Committee for the American Lung Association Asthma Clinical Research C, Holbrook JT, Wise RA, et al. Lansoprazole for children with poorly controlled asthma: a randomized controlled trial. *JAMA.* 2012;307(4):373-381.
189. Galmiche JP, Hatlebakk J, Attwood S, et al. Laparoscopic antireflux surgery vs esomeprazole treatment for chronic GERD: the LOTUS randomized clinical trial. *JAMA.* 2011;305(19):1969-1977.
190. Murphy GJ, Pike K, Rogers CA, et al. Liberal or restrictive transfusion after cardiac surgery. *N Engl J Med.* 2015;372(11):997-1008.
191. Carson JL, Terrin ML, Noveck H, et al. Liberal or restrictive transfusion in high-risk patients after hip surgery. *N Engl J Med.* 2011;365(26):2453-2462.

192. Osteras N, Hagen KB, Grotle M, Sand-Svartrud AL, Mowinckel P, Kjeken I. Limited effects of exercises in people with hand osteoarthritis: results from a randomized controlled trial. *Osteoarthritis Cartilage.* 2014;22(9):1224-1233.
193. Fassnacht M, Berruti A, Baudin E, et al. Linsitinib (OSI-906) versus placebo for patients with locally advanced or metastatic adrenocortical carcinoma: a double-blind, randomised, phase 3 study. *The Lancet Oncology.* 2015;16(4):426-435.
194. Van Herpe T, Mesotten D, Wouters PJ, et al. LOGIC-insulin algorithm-guided versus nurse-directed blood glucose control during critical illness: the LOGIC-1 single-center, randomized, controlled clinical trial. *Diabetes Care.* 2013;36(2):188-194.
195. Pepin JL, Cockcroft JR, Midwinter D, Sharma S, Rubin DB, Andreas S. Long-acting bronchodilators and arterial stiffness in patients with COPD: a comparison of fluticasone furoate/vilanterol with tiotropium. *Chest.* 2014;146(6):1521-1530.
196. Subotnik KL, Casaus LR, Ventura J, et al. Long-Acting Injectable Risperidone for Relapse Prevention and Control of Breakthrough Symptoms After a Recent First Episode of Schizophrenia. A Randomized Clinical Trial. *JAMA Psychiatry.* 2015;72(8):822-829.
197. Aitken ML, Bellon G, De Boeck K, et al. Long-term inhaled dry powder mannitol in cystic fibrosis: an international randomized study. *Am J Respir Crit Care Med.* 2012;185(6):645-652.
198. Tubiana R, Mandelbrot L, Le Chenadec J, et al. Lopinavir/ritonavir monotherapy as a nucleoside analogue-sparing strategy to prevent HIV-1 mother-to-child transmission: the ANRS 135 PRIMEVA phase 2/3 randomized trial. *Clin Infect Dis.* 2013;57(6):891-902.
199. Chamberlain JM, Okada P, Holsti M, et al. Lorazepam vs diazepam for pediatric status epilepticus: a randomized clinical trial. *JAMA.* 2014;311(16):1652-1660.
200. Brighton TA, Eikelboom JW, Mann K, et al. Low-dose aspirin for preventing recurrent venous thromboembolism. *N Engl J Med.* 2012;367(21):1979-1987.
201. Chen HH, Anstrom KJ, Givertz MM, et al. Low-dose dopamine or low-dose nesiritide in acute heart failure with renal dysfunction: the ROSE acute heart failure randomized trial. *JAMA.* 2013;310(23):2533-2543.
202. Buttgereit F, Mehta D, Kirwan J, et al. Low-dose prednisone chronotherapy for rheumatoid arthritis: a randomised clinical trial (CAPRA-2). *Ann Rheum Dis.* 2013;72(2):204-210.
203. Gligorov J, Doval D, Bines J, et al. Maintenance capecitabine and bevacizumab versus bevacizumab alone after initial first-line bevacizumab and docetaxel for patients with HER2-negative metastatic breast cancer (IMELDA): a randomised, open-label, phase 3 trial. *The Lancet Oncology.* 2014;15(12):1351-1360.
204. Stupp R, Taillibert S, Kanner AA, et al. Maintenance Therapy With Tumor-Treating Fields Plus Temozolomide vs Temozolomide Alone for Glioblastoma: A Randomized Clinical Trial. *JAMA.* 2015;314(23):2535-2543.
205. Vercelletto M, Boutoleau-Bretonniere C, Volteau C, et al. Memantine in behavioral variant frontotemporal dementia: negative results. *J Alzheimers Dis.* 2011;23(4):749-759.
206. Chak A, Buttar NS, Foster NR, et al. Metformin does not reduce markers of cell proliferation in esophageal tissues of patients with Barrett's esophagus. *Clin Gastroenterol Hepatol.* 2015;13(4):665-672 e661-664.
207. Finckh A, Mc Carthy GM, Madigan A, et al. Methotrexate in chronic-recurrent calcium pyrophosphate deposition disease: no significant effect in a randomized crossover trial. *Arthritis Res Ther.* 2014;16(5):458.

© 2019 Chen T et al. *JAMA Network Open*.

208. Hallsworth K, Thoma C, Hollingsworth KG, et al. Modified high-intensity interval training reduces liver fat and improves cardiac function in non-alcoholic fatty liver disease: a randomized controlled trial. *Clin Sci (Lond).* 2015;129(12):1097-1105.
209. Ostrosky-Zeichner L, Shoham S, Vazquez J, et al. MSG-01: A randomized, double-blind, placebo-controlled trial of caspofungin prophylaxis followed by preemptive therapy for invasive candidiasis in high-risk adults in the critical care setting. *Clin Infect Dis.* 2014;58(9):1219-1226.
210. Dekker LR, van der Voort PH, Simmers TA, et al. New image processing and noise reduction technology allows reduction of radiation exposure in complex electrophysiologic interventions while maintaining optimal image quality: a randomized clinical trial. *Heart Rhythm.* 2013;10(11):1678-1682.
211. Johnston RD, Stephenson MC, Crossland H, et al. No difference between high-fructose and high-glucose diets on liver triacylglycerol or biochemistry in healthy overweight men. *Gastroenterology.* 2013;145(5):1016-1025 e1012.
212. Jacobs AK, Normand SL, Massaro JM, et al. Nonemergency PCI at hospitals with or without on-site cardiac surgery. *N Engl J Med.* 2013;368(16):1498-1508.
213. Borel JC, Tamisier R, Gonzalez-Bermejo J, et al. Noninvasive ventilation in mild obesity hypoventilation syndrome: a randomized controlled trial. *Chest.* 2012;141(3):692-702.
214. van Lunzen J, Maggiolo F, Arribas JR, et al. Once daily dolutegravir (S/GSK1349572) in combination therapy in antiretroviral-naive adults with HIV: planned interim 48 week results from SPRING-1, a dose-ranging, randomised, phase 2b trial. *The Lancet Infectious Diseases.* 2012;12(2):111-118.
215. Sandborn WJ, Travis S, Moro L, et al. Once-daily budesonide MMX(R) extended-release tablets induce remission in patients with mild to moderate ulcerative colitis: results from the CORE I study. *Gastroenterology.* 2012;143(5):1218-1226 e1212.
216. Inagaki N, Onouchi H, Maezawa H, Kuroda S, Kaku K. Once-weekly trelagliptin versus daily alogliptin in Japanese patients with type 2 diabetes: a randomised, double-blind, phase 3, non-inferiority study. *The Lancet Diabetes & Endocrinology.* 2015;3(3):191-197.
217. Joensuu H, Eriksson M, Sundby Hall K, et al. One vs three years of adjuvant imatinib for operable gastrointestinal stromal tumor: a randomized trial. *JAMA.* 2012;307(12):1265-1272.
218. Adriaenssens T, Dens J, Ughi G, et al. Optical coherence tomography study of healing characteristics of paclitaxel-eluting balloons vs. everolimus-eluting stents for in-stent restenosis: the SEDUCE (Safety and Efficacy of a Drug elUting balloon in Coronary artery rEstenosis) randomised clinical trial. *EuroIntervention.* 2014;10(4):439-448.
219. Agweyu A, Gathara D, Oliwa J, et al. Oral amoxicillin versus benzyl penicillin for severe pneumonia among kenyan children: a pragmatic randomized controlled noninferiority trial. *Clin Infect Dis.* 2015;60(8):1216-1224.
220. Leung SS, Leung C, Lam TH, et al. Outcome of a postnatal depression screening programme using the Edinburgh Postnatal Depression Scale: a randomized controlled trial. *J Public Health (Oxf).* 2011;33(2):292-301.
221. Hong YS, Nam B-H, Kim K-p, et al. Oxaliplatin, fluorouracil, and leucovorin versus fluorouracil and leucovorin as adjuvant chemotherapy for locally advanced rectal cancer after preoperative chemoradiotherapy (ADORE): an open-label, multicentre, phase 2, randomised controlled trial. *The Lancet Oncology.* 2014;15(11):1245-1253.
222. van Nielen M, Feskens EJ, Rietman A, Siebelink E, Mensink M. Partly replacing meat protein with soy protein alters insulin resistance and blood lipids in postmenopausal women with abdominal obesity. *J Nutr.* 2014;144(9):1423-1429.

© 2019 Chen T et al. *JAMA Network Open*.

223. Calvert SB, Kramer JM, Anstrom KJ, Kaltenbach LA, Stafford JA, Allen LaPointe NM. Patient-focused intervention to improve long-term adherence to evidence-based medications: a randomized trial. *Am Heart J.* 2012;163(4):657-665 e651.
224. Calabresi PA, Kieseier BC, Arnold DL, et al. Pegylated interferon beta-1a for relapsing-remitting multiple sclerosis (ADVANCE): a randomised, phase 3, double-blind study. *The Lancet Neurology.* 2014;13(7):657-665.
225. Liu CH, Huang CF, Liu CJ, et al. Pegylated interferon-alpha2a with or without low-dose ribavirin for treatment-naive patients with hepatitis C virus genotype 1 receiving hemodialysis: a randomized trial. *Ann Intern Med.* 2013;159(11):729-738.
226. French JA, Krauss GL, Wechsler RT, et al. Perampanel for tonic-clonic seizures in idiopathic generalized epilepsy A randomized trial. *Neurology.* 2015;85(11):950-957.
227. Gallagher TQ, Hill C, Ojha S, et al. Perioperative dexamethasone administration and risk of bleeding following tonsillectomy in children: a randomized controlled trial. *JAMA.* 2012;308(12):1221-1226.
228. Duggal N, Poddatoori V, Noroozkhani S, Siddik-Ahmad RI, Caughey AB. Perioperative oxygen supplementation and surgical site infection after cesarean delivery: a randomized trial. *Obstet Gynecol.* 2013;122(1):79-84.
229. Marfella R, Sasso FC, Siniscalchi M, et al. Peri-procedural tight glycemic control during early percutaneous coronary intervention is associated with a lower rate of in-stent restenosis in patients with acute ST-elevation myocardial infarction. *J Clin Endocrinol Metab.* 2012;97(8):2862-2871.
230. Ruzsa A, Sen M, Evans M, et al. Phase 2, open-label, 1:1 randomized controlled trial exploring the efficacy of EMD 1201081 in combination with cetuximab in second-line cetuximab-naive patients with recurrent or metastatic squamous cell carcinoma of the head and neck (R/M SCCHN). *Invest New Drugs.* 2014;32(6):1278-1284.
231. Pfutzner A, Schondorf T, Tschope D, et al. PIOfix-study: effects of pioglitazone/metformin fixed combination in comparison with a combination of metformin with glimepiride on diabetic dyslipidemia. *Diabetes Technol Ther.* 2011;13(6):637-643.
232. Liu PY, Lin LY, Lin HJ, et al. Pitavastatin and Atorvastatin double-blind randomized comPArative study among hiGh-risk patients, including thOse with Type 2 diabetes mellitus, in Taiwan (PAPAGO-T Study). *PLoS One.* 2013;8(10):e76298.
233. Kinney MA, Mantilla CB, Carns PE, et al. Preoperative gabapentin for acute post-thoracotomy analgesia: a randomized, double-blinded, active placebo-controlled study. *Pain Pract.* 2012;12(3):175-183.
234. Scheiman JM, Devereaux PJ, Herlitz J, et al. Prevention of peptic ulcers with esomeprazole in patients at risk of ulcer development treated with low-dose acetylsalicylic acid: a randomised, controlled trial (OBERON). *Heart.* 2011;97(10):797-802.
235. Meredith IT, Verheye S, Dubois CL, et al. Primary endpoint results of the EVOLVE trial: a randomized evaluation of a novel bioabsorbable polymer-coated, everolimus-eluting coronary stent. *J Am Coll Cardiol.* 2012;59(15):1362-1370.
236. Zinner RG, Obasaju CK, Spigel DR, et al. PRONOUNCE: randomized, open-label, phase III study of first-line pemetrexed + carboplatin followed by maintenance pemetrexed versus paclitaxel + carboplatin + bevacizumab followed by maintenance bevacizumab in patients ith advanced nonsquamous non-small-cell lung cancer. *J Thorac Oncol.* 2015;10(1):134-142.
237. Heo K, Kang JK, Choi CM, Lee MS, Noh KW, Kim SB. Prophylactic effect of erythropoietin injection to prevent acute mountain sickness: an open-label randomized controlled trial. *J Korean Med Sci.* 2014;29(3):416-422.

© 2019 Chen T et al. *JAMA Network Open*.

238. Rojas MA, Lozano JM, Rojas MX, et al. Prophylactic probiotics to prevent death and nosocomial infection in preterm infants. *Pediatrics.* 2012;130(5):e1113-1120.
239. Prymula R, Bergsaker MR, Esposito S, et al. Protection against varicella with two doses of combined measles-mumps-rubella-varicella vaccine versus one dose of monovalent varicella vaccine: a multicentre, observer-blind, randomised, controlled trial. *The Lancet.* 2014;383(9925):1313-1324.
240. Read TR, Hocking JS, Bradshaw CS, et al. Provision of rapid HIV tests within a health service and frequency of HIV testing among men who have sex with men: randomised controlled trial. *BMJ.* 2013;347:f5086.
241. Galace de Freitas D, Marcondes FB, Monteiro RL, Rosa SG, Maria de Moraes Barros Fucs P, Fukuda TY. Pulsed electromagnetic field and exercises in patients with shoulder impingement syndrome: a randomized, double-blind, placebo-controlled clinical trial. *Arch Phys Med Rehabil.* 2014;95(2):345-352.
242. Kianimehr G, Fatehi F, Hashempoor S, et al. Raloxifene adjunctive therapy for postmenopausal women suffering from chronic schizophrenia: a randomized double-blind and placebo controlled trial. *Daru.* 2014;22:55.
243. De Waele JJ, Tellado JM, Alder J, et al. Randomised clinical trial of moxifloxacin versus ertapenem in complicated intra-abdominal infections: results of the PROMISE study. *Int J Antimicrob Agents.* 2013;41(1):57-64.
244. Holm NR, Sindberg B, Schou M, et al. Randomised comparison of manual compression and FemoSeal vascular closure device for closure after femoral artery access coronary angiography: the CLOSure dEvices Used in everyday Practice (CLOSE-UP) study. *EuroIntervention.* 2014;10(2):183-190.
245. Cox SE, Nweneka CV, Doherty CP, Fulford AJ, Moore SE, Prentice AM. Randomised controlled trial of weekly chloroquine to re-establish normal erythron iron flux and haemoglobin recovery in postmalarial anaemia. *BMJ Open.* 2013;3(7).
246. Bilkhu PS, Naroo SA, Wolffsohn JS. Randomised masked clinical trial of the MGDRx EyeBag for the treatment of meibomian gland dysfunction-related evaporative dry eye. *Br J Ophthalmol.* 2014;98(12):1707-1711.
247. Souglakos J, Ziras N, Kakolyris S, et al. Randomised phase-II trial of CAPIRI (capecitabine, irinotecan) plus bevacizumab vs FOLFIRI (folinic acid, 5-fluorouracil, irinotecan) plus bevacizumab as first-line treatment of patients with unresectable/metastatic colorectal cancer (mCRC). *Br J Cancer.* 2012;106(3):453-459.
248. Echeverria P, Bonjoch A, Puig J, et al. Randomised study to assess the efficacy and safety of once-daily etravirine-based regimen as a switching strategy in HIV-infected patients receiving a protease inhibitor-containing regimen. Etraswitch study. *PLoS One.* 2014;9(2):e84676.
249. Wolthuis AM, Fieuws S, Van Den Bosch A, de Buck van Overstraeten A, D'Hoore A. Randomized clinical trial of laparoscopic colectomy with or without natural-orifice specimen extraction. *Br J Surg.* 2015;102(6):630-637.
250. Sorensen LS, Thorlacius-Ussing O, Schmidt EB, et al. Randomized clinical trial of perioperative omega-3 fatty acid supplements in elective colorectal cancer surgery. *Br J Surg.* 2014;101(2):33-42.
251. Liu N, Cadilhac DA, Andrew NE, et al. Randomized controlled trial of early rehabilitation after intracerebral hemorrhage stroke: difference in outcomes within 6 months of stroke. *Stroke.* 2014;45(12):3502-3507.
252. Huang YH, Hsiao LT, Hong YC, et al. Randomized controlled trial of entecavir prophylaxis for rituximab-associated hepatitis B virus reactivation in patients with lymphoma and resolved hepatitis B. *J Clin Oncol.* 2013;31(22):2765-2772.

© 2019 Chen T et al. *JAMA Network Open*.

253. McCollum PT, Bush JA, James G, et al. Randomized phase II clinical trial of avotermin versus placebo for scar improvement. *Br J Surg.* 2011;98(7):925-934.
254. Morizane C, Okusaka T, Mizusawa J, et al. Randomized phase II study of gemcitabine plus S-1 versus S-1 in advanced biliary tract cancer: a Japan Clinical Oncology Group trial (JCOG 0805). *Cancer Sci.* 2013;104(9):1211-1216.
255. Reid RD, Morrin LI, Beaton LJ, et al. Randomized trial of an internet-based computer-tailored expert system for physical activity in patients with heart disease. *Eur J Prev Cardiol.* 2012;19(6):1357-1364.
256. Bronowicki JP, Ratziu V, Gadano A, et al. Randomized trial of asunaprevir plus peginterferon alfa and ribavirin for previously untreated genotype 1 or 4 chronic hepatitis C. *J Hepatol.* 2014;61(6):1220-1227.
257. Bocquet N, Sergent Alaoui A, Jais JP, et al. Randomized trial of oral versus sequential IV/oral antibiotic for acute pyelonephritis in children. *Pediatrics.* 2012;129(2):e269-275.
258. Steindorf K, Schmidt ME, Klassen O, et al. Randomized, controlled trial of resistance training in breast cancer patients receiving adjuvant radiotherapy: results on cancer-related fatigue and quality of life. *Ann Oncol.* 2014;25(11):2237-2243.
259. Draganov PV, Chang MN, Alkhasawneh A, et al. Randomized, controlled trial of standard, large-capacity versus jumbo biopsy forceps for polypectomy of small, sessile, colorectal polyps. *Gastrointest Endosc.* 2012;75(1):118-126.
260. Oldach D, Clark K, Schranz J, et al. Randomized, double-blind, multicenter phase 2 study comparing the efficacy and safety of oral solithromycin (CEM-101) to those of oral levofloxacin in the treatment of patients with community-acquired bacterial pneumonia. *Antimicrob Agents Chemother.* 2013;57(6):2526-2534.
261. Bornhäuser M, Kienast J, Trenschel R, et al. Reduced-intensity conditioning versus standard conditioning before allogeneic haemopoietic cell transplantation in patients with acute myeloid leukaemia in first complete remission: a prospective, open-label randomised phase 3 trial. *The Lancet Oncology.* 2012;13(10):1035-1044.
262. Hougaard KD, Hjort N, Zeidler D, et al. Remote ischemic perconditioning as an adjunct therapy to thrombolysis in patients with acute ischemic stroke: a randomized trial. *Stroke.* 2014;45(1):159-167.
263. Vesikari T, Karvonen A, Borrow R, et al. Results from a randomized clinical trial of coadministration of RotaTeq, a pentavalent rotavirus vaccine, and NeisVac-C, a meningococcal serogroup C conjugate vaccine. *Clin Vaccine Immunol.* 2011;18(5):878-884.
264. Rock CL, Flatt SW, Byers TE, et al. Results of the Exercise and Nutrition to Enhance Recovery and Good Health for You (ENERGY) Trial: A Behavioral Weight Loss Intervention in Overweight or Obese Breast Cancer Survivors. *J Clin Oncol.* 2015;33(28):3169-3176.
265. van der Made SM, Plat J, Mensink RP. Resveratrol does not influence metabolic risk markers related to cardiovascular health in overweight and slightly obese subjects: a randomized, placebo-controlled crossover trial. *PLoS One.* 2015;10(3):e0118393.
266. Chiu TF, Chen LL, Su DH, et al. Rhodiola crenulata extract for prevention of acute mountain sickness: a randomized, double-blind, placebo-controlled, crossover trial. *BMC Complement Altern Med.* 2013;13:298.
267. Iveson T, Donehower RC, Davidenko I, et al. Rilotumumab in combination with epirubicin, cisplatin, and capecitabine as first-line treatment for gastric or oesophagogastric junction adenocarcinoma: an open-label, dose de-escalation phase 1b study and a double-blind, randomised phase 2 study. *The Lancet Oncology.* 2014;15(9):1007-1018.

© 2019 Chen T et al. *JAMA Network Open*.

268. Boesten JE, Kaper J, Stoffers HE, Kroon AA, van Schayck OC. Rimonabant improves obesity but not the overall cardiovascular risk and quality of life; results from CARDIO-REDUSE (CArdiometabolic Risk reDuctIOn by Rimonabant: the Effectiveness in Daily practice and its USE). *Fam Pract.* 2012;29(5):521-527.
269. Ghofrani HA, D'Armini AM, Grimminger F, et al. Riociguat for the treatment of chronic thromboembolic pulmonary hypertension. *N Engl J Med.* 2013;369(4):319-329.
270. Guillevin L, Pagnoux C, Karras A, et al. Rituximab versus azathioprine for maintenance in ANCA-associated vasculitis. *N Engl J Med.* 2014;371(19):1771-1780.
271. Devos D, Moreau C, Maltete D, et al. Rivastigmine in apathetic but dementia and depression-free patients with Parkinson's disease: a double-blind, placebo-controlled, randomised clinical trial. *J Neurol Neurosurg Psychiatry.* 2014;85(6):668-674.
272. Capussotti L, Ferrero A, Russolillo N, Langella S, Lo Tesoriere R, Vigano L. Routine anterior approach during right hepatectomy: results of a prospective randomised controlled trial. *J Gastrointest Surg.* 2012;16(7):1324-1332.
273. Munster JM, Leenders AC, Hamilton CJ, et al. Routine screening for Coxiella burnetii infection during pregnancy: a clustered randomised controlled trial during an outbreak, the Netherlands, 2010. *Euro Surveill.* 2013;18(24).
274. McCready J, Chow AW, Lowe MD, et al. Safety and efficacy of multipolar pulmonary vein ablation catheter vs. irrigated radiofrequency ablation for paroxysmal atrial fibrillation: a randomized multicentre trial. *Europace.* 2014;16(8):1145-1153.
275. Rigby W, Tony HP, Oelke K, et al. Safety and efficacy of ocrelizumab in patients with rheumatoid arthritis and an inadequate response to methotrexate: results of a forty-eight-week randomized, double-blind, placebo-controlled, parallel-group phase III trial. *Arthritis Rheum.* 2012;64(2):350-359.
276. Gray GE, Allen M, Moodie Z, et al. Safety and efficacy of the HVTN 503/Phambili Study of a clade-B-based HIV-1 vaccine in South Africa: a double-blind, randomised, placebo-controlled test-of-concept phase 2b study. *The Lancet Infectious Diseases.* 2011;11(7):507-515.
277. Siebler M, Hennerici MG, Schneider D, et al. Safety of Tirofiban in acute Ischemic Stroke: the SaTIS trial. *Stroke.* 2011;42(9):2388-2392.
278. Sitbon O, Channick R, Chin KM, et al. Selexipag for the Treatment of Pulmonary Arterial Hypertension. *N Engl J Med.* 2015;373(26):2522-2533.
279. Zhang M, Tong G, Liu Y, et al. Sequential Versus Continual Purified Urinary FSH/hCG in Men With Idiopathic Hypogonadotropic Hypogonadism. *J Clin Endocrinol Metab.* 2015;100(6):2449-2455.
280. Jenkins R, Othieno C, Okeyo S, et al. Short structured general mental health in service training programme in Kenya improves patient health and social outcomes but not detection of mental health problems - a pragmatic cluster randomised controlled trial. *Int J Ment Health Syst.* 2013;7(1):25.
281. Imaz A, Olmo M, Penaranda M, et al. Short-term and long-term clinical and immunological consequences of stopping antiretroviral therapy in HIV-infected patients with preserved immune function. *Antivir Ther.* 2013;18(1):125-130.
282. Denham JW, Joseph D, Lamb DS, et al. Short-term androgen suppression and radiotherapy versus intermediate-term androgen suppression and radiotherapy, with or without zoledronic acid, in men with locally advanced prostate cancer (TROG 03.04 RADAR): an open-label, randomised, phase 3 factorial trial. *The Lancet Oncology.* 2014;15(10):1076-1089.
283. Andersen MJ, Ersboll M, Axelsson A, et al. Sildenafil and diastolic dysfunction after acute myocardial infarction in patients with preserved ejection fraction: the Sildenafil and Diastolic Dysfunction After Acute Myocardial Infarction (SIDAMI) trial. *Circulation.* 2013;127(11):1200-1208.

© 2019 Chen T et al. *JAMA Network Open*.

284. Kim ST, Kang JH, Lee J, et al. Simvastatin plus capecitabine-cisplatin versus placebo plus capecitabine-cisplatin in patients with previously untreated advanced gastric cancer: a double-blind randomised phase 3 study. *Eur J Cancer.* 2014;50(16):2822-2830.
285. Friman S, Arns W, Nashan B, et al. Sotrastaurin, a novel small molecule inhibiting protein-kinase C: randomized phase II study in renal transplant recipients. *Am J Transplant.* 2011;11(7):1444-1455.
286. Gralnek IM, Siersema PD, Halpern Z, et al. Standard forward-viewing colonoscopy versus full-spectrum endoscopy: an international, multicentre, randomised, tandem colonoscopy trial. *The Lancet Oncology.* 2014;15(3):353-360.
287. Patel A, Stern L, Unger Z, et al. Staying on track: a cluster randomized controlled trial of automated reminders aimed at increasing human papillomavirus vaccine completion. *Vaccine.* 2014;32(21):2428-2433.
288. Camenzind E, Wijns W, Mauri L, et al. Stent thrombosis and major clinical events at 3 years after zotarolimus-eluting or sirolimus-eluting coronary stent implantation: a randomised, multicentre, open-label, controlled trial. *The Lancet.* 2012;380(9851):1396-1405.
289. Kivitz A, Olech E, Borofsky M, et al. Subcutaneous tocilizumab versus placebo in combination with disease-modifying antirheumatic drugs in patients with rheumatoid arthritis. *Arthritis Care Res (Hoboken).* 2014;66(11):1653-1661.
290. Smith PK, Puskas JD, Ascheim DD, et al. Surgical treatment of moderate ischemic mitral regurgitation. *N Engl J Med.* 2014;371(23):2178-2188.
291. Mackeen AD, Khalifeh A, Fleisher J, et al. Suture compared with staple skin closure after cesarean delivery: a randomized controlled trial. *Obstet Gynecol.* 2014;123(6):1169-1175.
292. Jourdain G, Le Coeur S, Ngo-Giang-Huong N, et al. Switching HIV treatment in adults based on CD4 count versus viral load monitoring: a randomized, non-inferiority trial in Thailand. *PLoS Med.* 2013;10(8):e1001494.
293. Kim YC, Chin HJ, Koo HS, Kim S. Tacrolimus decreases albuminuria in patients with IgA nephropathy and normal blood pressure: a double-blind randomized controlled trial of efficacy of tacrolimus on IgA nephropathy. *PLoS One.* 2013;8(8):e71545.
294. Biondi D, Xiang J, Benson C, Etropolski M, Moskovitz B, Rauschkolb C. Tapentadol immediate release versus oxycodone immediate release for treatment of acute low back pain. *Pain Physician.* 2013;16(3):E237-246.
295. Verweij LM, Proper KI, Weel AN, Hulshof CT, van Mechelen W. The application of an occupational health guideline reduces sedentary behaviour and increases fruit intake at work: results from an RCT. *Occup Environ Med.* 2012;69(7):500-507.
296. Ou G, Svarta S, Chan C, Galorport C, Qian H, Enns R. The effect of chewing gum on small-bowel transit time in capsule endoscopy: a prospective, randomized trial. *Gastrointest Endosc.* 2014;79(4):630-636.
297. Son I, Oh CS, Choi JW, Kim SH. The effect of sufentanil administration on remifentanil-based anaesthesia during laparoscopic gynaecological surgery: a double-blind randomized controlled trial. *ScientificWorldJournal.* 2014;2014:701329.
298. Leaker BR, Singh D, Ali FY, Barnes PJ, O'Connor B. The effect of the novel phosphodiesterase-4 inhibitor MEM 1414 on the allergen induced responses in mild asthma. *BMC Pulm Med.* 2014;14:166.
299. Kinley J, Stone L, Dewey M, et al. The effect of using high facilitation when implementing the Gold Standards Framework in Care Homes programme: a cluster randomised controlled trial. *Palliat Med.* 2014;28(9):1099-1109.
300. Blumenthal JA, Emery CF, Smith PJ, et al. The effects of a telehealth coping skills intervention on outcomes in chronic obstructive pulmonary disease: primary results from the INSPIRE-II study. *Psychosom Med.* 2014;76(8):581-592.

© 2019 Chen T et al. *JAMA Network Open*.

301. Lee EB, Fleischmann R, Hall S, et al. Tofacitinib versus methotrexate in rheumatoid arthritis. *N Engl J Med.* 2014;370(25):2377-2386.
302. Danese S, Rudzinski J, Brandt W, et al. Tralokinumab for moderate-to-severe UC: a randomised, double-blind, placebo-controlled, phase IIa study. *Gut.* 2015;64(2):243-249.
303. Prick BW, Jansen AJ, Steegers EA, et al. Transfusion policy after severe postpartum haemorrhage: a randomised non-inferiority trial. *BJOG.* 2014;121(8):1005-1014.
304. Rejeski WJ, Brubaker PH, Goff DC, Jr., et al. Translating weight loss and physical activity programs into the community to preserve mobility in older, obese adults in poor cardiovascular health. *Arch Intern Med.* 2011;171(10):880-886.
305. Walter MC, Reilich P, Thiele S, et al. Treatment of dysferlinopathy with deflazacort: a double-blind, placebo-controlled clinical trial. *Orphanet J Rare Dis.* 2013;8:26.
306. Chetchotisakd P, Chierakul W, Chaowagul W, et al. Trimethoprim-sulfamethoxazole versus trimethoprim-sulfamethoxazole plus doxycycline as oral eradicative treatment for melioidosis (MERTH): a multicentre, double-blind, non-inferiority, randomised controlled trial. *The Lancet.* 2014;383(9919):807-814.
307. Duiverman ML, Wempe JB, Bladder G, et al. Two-year home-based nocturnal noninvasive ventilation added to rehabilitation in chronic obstructive pulmonary disease patients: a randomized controlled trial. *Respir Res.* 2011;12:112.
308. Chappell LC, Gurung V, Seed PT, et al. Ursodeoxycholic acid versus placebo, and early term delivery versus expectant management, in women with intrahepatic cholestasis of pregnancy: semifactorial randomised clinical trial. *BMJ.* 2012;344:e3799.
309. Buse JB, Bergenstal RM, Glass LC, et al. Use of twice-daily exenatide in Basal insulin-treated patients with type 2 diabetes: a randomized, controlled trial. *Ann Intern Med.* 2011;154(2):103-112.
310. Yi TJ, Shannon B, Chieza L, et al. Valacyclovir therapy does not reverse herpes-associated alterations in cervical immunology: a randomized, placebo-controlled crossover trial. *J Infect Dis.* 2014;210(5):708-712.
311. Larsen M, Schmidt-Erfurth U, Lanzetta P, et al. Verteporfin plus ranibizumab for choroidal neovascularization in age-related macular degeneration: twelve-month MONT BLANC study results. *Ophthalmology.* 2012;119(5):992-1000.
312. Yu TC, Hamill JK, Liley A, Hill AG. Warm, humidified carbon dioxide gas insufflation for laparoscopic appendicectomy in children: a double-blinded randomized controlled trial. *Ann Surg.* 2013;257(1):44-53.
313. Valgimigli M, Patialiakas A, Thury A, et al. Zotarolimus-eluting versus bare-metal stents in uncertain drug-eluting stent candidates. *J Am Coll Cardiol.* 2015;65(8):805-815.
314. Cleland JG, Calvert M, Freemantle N, et al. The Heart Failure Revascularisation Trial (HEART). *Eur J Heart Fail.* 2011;13(2):227-233.
315. Guarany FC, Picon PD, Guarany NR, et al. A double-blind, randomised, crossover trial of two botulinum toxin type a in patients with spasticity. *PLoS One.* 2013;8(2):e56479.
316. Blanch N, Clifton PM, Petersen KS, Willoughby SR, Keogh JB. Effect of high potassium diet on endothelial function. *Nutr Metab Cardiovasc Dis.* 2014;24(9):983-989.
317. Maki KC, Lawless AL, Kelley KM, et al. Effects of prescription omega-3-acid ethyl esters on fasting lipid profile in subjects with primary hypercholesterolemia. *J Cardiovasc Pharmacol.* 2011;57(4):489-494.

© 2019 Chen T et al. *JAMA Network Open*.

318. Shechter A, Rising R, Albu JB, St-Onge MP. Experimental sleep curtailment causes wake-dependent increases in 24-h energy expenditure as measured by whole-room indirect calorimetry. *Am J Clin Nutr.* 2013;98(6):1433-1439.
319. Green EA, Black BK, Biaggioni I, et al. Melatonin reduces tachycardia in postural tachycardia syndrome: a randomized, crossover trial. *Cardiovasc Ther.* 2014;32(3):105-112.
320. Vlassara H, Uribarri J, Cai W, et al. Effects of sevelamer on HbA1c, inflammation, and advanced glycation end products in diabetic kidney disease. *Clin J Am Soc Nephrol.* 2012;7(6):934-942.
321. Nadjarzadeh A, Sadeghi MR, Amirjannati N, et al. Coenzyme Q10 improves seminal oxidative defense but does not affect on semen parameters in idiopathic oligoasthenoteratozoospermia: a randomized double-blind, placebo controlled trial. *J Endocrinol Invest.* 2011;34(8):e224-228.
322. Saharkhiz N, Nikbakht R, Salehpour S. Comparison between Conventional Blind Embryo Transfer and Embryo Transfer Based on Previously Measured Uterine Length. *Int J Fertil Steril.* 2014;8(3):249-254.
323. Buhl M, Bosnjak E, Vendelbo MH, et al. Direct effects of locally administered lipopolysaccharide on glucose, lipid, and protein metabolism in the placebo-controlled, bilaterally infused human leg. *J Clin Endocrinol Metab.* 2013;98(5):2090-2099.
324. Petersen M, Andersen JT, Jimenez-Solem E, et al. Effect of the Arg389Gly beta(1)-adrenoceptor polymorphism on plasma renin activity and heart rate, and the genotype-dependent response to metoprolol treatment. *Clin Exp Pharmacol Physiol.* 2012;39(9):779-785.
325. Hong DM, Jeon Y, Lee C-S, et al. Effects of Remote Ischemic Preconditioning With Postconditioning in Patients Undergoing Off-Pump Coronary Artery Bypass Surgery. *Circulation Journal.* 2012;76(4):884-890.
326. Poleshuck EL, Gamble SA, Bellenger K, et al. Randomized controlled trial of interpersonal psychotherapy versus enhanced treatment as usual for women with co-occurring depression and pelvic pain. *J Psychosom Res.* 2014;77(4):264-272.
327. Ramaiya KJ, Blinder KJ, Ciulla T, Cooper B, Shah GK. Ranibizumab versus photodynamic therapy for presumed ocular histoplasmosis syndrome. *Ophthalmic Surg Lasers Imaging Retina.* 2013;44(1):17-21.
328. Monteiro SM, Jancey J, Dhaliwal SS, et al. Results of a randomized controlled trial to promote physical activity behaviours in mothers with young children. *Prev Med.* 2014;59:12-18.
329. Tensil MD, Jonas B, Struber E. Two fully automated web-based interventions for risky alcohol use: randomized controlled trial. *J Med Internet Res.* 2013;15(6):e110.
330. Van Braeckel E, Bourguignon P, Koutsoukos M, et al. An adjuvanted polyprotein HIV-1 vaccine induces polyfunctional cross-reactive CD4+ T cell responses in seronegative volunteers. *Clin Infect Dis.* 2011;52(4):522-531.
331. Landewe R, Braun J, Deodhar A, et al. Efficacy of certolizumab pegol on signs and symptoms of axial spondyloarthritis including ankylosing spondylitis: 24-week results of a double-blind randomised placebo-controlled Phase 3 study. *Ann Rheum Dis.* 2014;73(1):39-47.
332. Sands BE, Sandborn WJ, Creed TJ, et al. Basiliximab does not increase efficacy of corticosteroids in patients with steroid-refractory ulcerative colitis. *Gastroenterology.* 2012;143(2):356-364 e351.
333. Wong BS, Camilleri M, McKinzie S, Burton D, Graffner H, Zinsmeister AR. Effects of A3309, an ileal bile acid transporter inhibitor, on colonic transit and symptoms in females with functional constipation. *Am J Gastroenterol.* 2011;106(12):2154-2164.

© 2019 Chen T et al. *JAMA Network Open*.

334. Rogoveanu OC, Mogosanu GD, Bejenaru C, et al. Effects of Calcium Fructoborate on Levels of C-Reactive Protein, Total Cholesterol, Low-Density Lipoprotein, Triglycerides, IL-1beta, IL-6, and MCP-1: a Double-blind, Placebo-controlled Clinical Study. *Biol Trace Elem Res.* 2015;163(1-2):124-131.
335. Kerwin EM, D'Urzo AD, Gelb AF, et al. Efficacy and safety of a 12-week treatment with twice-daily aclidinium bromide in COPD patients (ACCORD COPD I). *COPD.* 2012;9(2):90-101.
336. Khullar V, Amarenco G, Angulo JC, et al. Efficacy and tolerability of mirabegron, a beta(3)-adrenoceptor agonist, in patients with overactive bladder: results from a randomised European-Australian phase 3 trial. *Eur Urol.* 2013;63(2):283-295.
337. Hsu PI, Lai KH, Liu CP. Esomeprazole with clopidogrel reduces peptic ulcer recurrence, compared with clopidogrel alone, in patients with atherosclerosis. *Gastroenterology.* 2011;140(3):791-798.
338. Somers JM, Rezansoff SN, Moniruzzaman A, Palepu A, Patterson M. Housing first reduces re-offending among formerly homeless adults with mental disorders: results of a randomized controlled trial. *PLoS One.* 2013;8(9):e72946.
339. Ferrara LA, Pacioni D, Di Fronzo V, et al. Low-lipid diet reduces frequency and severity of acute migraine attacks. *Nutr Metab Cardiovasc Dis.* 2015;25(4):370-375.
340. Roussell MA, Hill AM, Gaugler TL, et al. Beef in an Optimal Lean Diet study: effects on lipids, lipoproteins, and apolipoproteins. *Am J Clin Nutr.* 2012;95(1):9-16.
341. Andersen TR, Schmidt JF, Nielsen JJ, et al. Effect of football or strength training on functional ability and physical performance in untrained old men. *Scand J Med Sci Sports.* 2014;24 Suppl 1:76-85.
342. Birnbaum A, Schechter C, Tufaro V, Touger R, Gallagher EJ, Bijur P. Efficacy of patient-controlled analgesia for patients with acute abdominal pain in the emergency department: a randomized trial. *Acad Emerg Med.* 2012;19(4):370-377.
343. Aiello AE, Perez V, Coulborn RM, Davis BM, Uddin M, Monto AS. Facemasks, hand hygiene, and influenza among young adults: a randomized intervention trial. *PLoS One.* 2012;7(1):e29744.
344. Flynn PM, Cunningham CK, Rudy B, et al. Hepatitis B vaccination in HIV-infected youth: a randomized trial of three regimens. *J Acquir Immune Defic Syndr.* 2011;56(4):325-332.
345. Clemson L, Fiatarone Singh MA, Bundy A, et al. Integration of balance and strength training into daily life activity to reduce rate of falls in older people (the LiFE study): randomised parallel trial. *BMJ.* 2012;345:e4547.
346. Robidoux A, Tang G, Rastogi P, et al. Lapatinib as a component of neoadjuvant therapy for HER2-positive operable breast cancer (NSABP protocol B-41): an open-label, randomised phase 3 trial. *The Lancet Oncology.* 2013;14(12):1183-1192.
347. von Pawel J, Harvey JH, Spigel DR, et al. Phase II trial of mapatumumab, a fully human agonist monoclonal antibody to tumor necrosis factor-related apoptosis-inducing ligand receptor 1 (TRAIL-R1), in combination with paclitaxel and carboplatin in patients with advanced non-small-cell lung cancer. *Clin Lung Cancer.* 2014;15(3):188-196 e182.
348. Investigators NETiPDF-Z. Pioglitazone in early Parkinson's disease: a phase 2, multicentre, double-blind, randomised trial. *Lancet Neurol.* 2015;14(8):795-803.
349. Bartelmaos T, Chabanel A, Leger J, et al. Plasma transfusion in liver transplantation: a randomized, double-blind, multicenter clinical comparison of three virally secured plasmas. *Transfusion.* 2013;53(6):1335-1345.

© 2019 Chen T et al. *JAMA Network Open*.

350. Matsuzaki M, Ogihara T, Umemoto S, et al. Prevention of cardiovascular events with calcium channel blocker-based combination therapies in patients with hypertension: a randomized controlled trial. *J Hypertens.* 2011;29(8):1649-1659.
351. Yelland MJ, Sweeting KR, Lyftogt JA, Ng SK, Scuffham PA, Evans KA. Prolotherapy injections and eccentric loading exercises for painful Achilles tendinosis: a randomised trial. *Br J Sports Med.* 2011;45(5):421-428.
352. Hasin DS, Aharonovich E, O'Leary A, et al. Reducing heavy drinking in HIV primary care: a randomized trial of brief intervention, with and without technological enhancement. *Addiction.* 2013;108(7):1230-1240.
353. Watanaveeradej V, Gibbons RV, Simasathien S, et al. Safety and immunogenicity of a rederived, live-attenuated dengue virus vaccine in healthy adults living in Thailand: a randomized trial. *Am J Trop Med Hyg.* 2014;91(1):119-128.
354. Barber JP, Barrett MS, Gallop R, Rynn MA, Rickels K. Short-term dynamic psychotherapy versus pharmacotherapy for major depressive disorder: a randomized, placebo-controlled trial. *J Clin Psychiatry.* 2012;73(1):66-73.
355. Emery P, Hammoudeh M, FitzGerald O, et al. Sustained remission with etanercept tapering in early rheumatoid arthritis. *N Engl J Med.* 2014;371(19):1781-1792.
356. Négrier S, Gravis G, Pérol D, et al. Temsirolimus and bevacizumab, or sunitinib, or interferon alfa and bevacizumab for patients with advanced renal cell carcinoma (TORAVA): a randomised phase 2 trial. *The Lancet Oncology.* 2011;12(7):673-680.
357. Adachi M, Taniguchi H, Tohda Y, et al. The efficacy and tolerability of intravenous montelukast in acute asthma exacerbations in Japanese patients. *J Asthma.* 2012;49(6):649-656.
358. Koenig MK, Hebert AA, Roberson J, et al. Topical rapamycin therapy to alleviate the cutaneous manifestations of tuberous sclerosis complex: a double-blind, randomized, controlled trial to evaluate the safety and efficacy of topically applied rapamycin. *Drugs R D.* 2012;12(3):121-126.
359. Donnez J, Tomaszewski J, Vazquez F, et al. Ulipristal acetate versus leuprolide acetate for uterine fibroids. *N Engl J Med.* 2012;366(5):421-432.
360. Gadde KM, Kopping MF, Wagner HR, 2nd, Yonish GM, Allison DB, Bray GA. Zonisamide for weight reduction in obese adults: a 1-year randomized controlled trial. *Arch Intern Med.* 2012;172(20):1557-1564.
361. Mahler DA, Decramer M, D'Urzo A, et al. Dual bronchodilation with QVA149 reduces patient-reported dyspnoea in COPD: the BLAZE study. *Eur Respir J.* 2014;43(6):1599-1609.
362. Mahableshwarkar AR, Jacobsen PL, Serenko M, Chen Y, Trivedi MH. A randomized, double-blind, placebo-controlled study of the efficacy and safety of 2 doses of vortioxetine in adults with major depressive disorder. *J Clin Psychiatry.* 2015;76(5):583-591.
363. Lee CM, Chen CY, Chien RN, et al. A double-blind randomized controlled study to evaluate the efficacy of low-dose oral interferon-alpha in preventing hepatitis C relapse. *J Interferon Cytokine Res.* 2014;34(3):187-194.
364. Blondell RD, Frydrych LM, Jaanimagi U, et al. A randomized trial of two behavioral interventions to improve outcomes following inpatient detoxification for alcohol dependence. *J Addict Dis.* 2011;30(2):136-148.
365. Torp-Pedersen C, Raev DH, Dickinson G, Butterfield NN, Mangal B, Beatch GN. A randomized, placebo-controlled study of vernakalant (oral) for the prevention of atrial fibrillation recurrence after cardioversion. *Circ Arrhythm Electrophysiol.* 2011;4(5):637-643.
366. Bateman LA, Slentz CA, Willis LH, et al. Comparison of aerobic versus resistance exercise training effects on metabolic syndrome (from the Studies of a Targeted Risk Reduction Intervention Through Defined Exercise - STRRIDE-AT/RT). *Am J Cardiol.* 2011;108(6):838-844.

© 2019 Chen T et al. *JAMA Network Open*.

367. Collins EG, Jelinek C, O'Connell S, et al. Contrasting breathing retraining and helium-oxygen during pulmonary rehabilitation in COPD: a randomized clinical trial. *Respir Med.* 2014;108(2):297-306.
368. Ison MG, Szakaly P, Shapira MY, Krivan G, Nist A, Dutkowski R. Efficacy and safety of oral oseltamivir for influenza prophylaxis in transplant recipients. *Antivir Ther.* 2012;17(6):955-964.
369. Cushman WC, Bakris GL, White WB, et al. Azilsartan medoxomil plus chlorthalidone reduces blood pressure more effectively than olmesartan plus hydrochlorothiazide in stage 2 systolic hypertension. *Hypertension.* 2012;60(2):310-318.
370. Teshima CW, Zepeda-Gomez S, AlShankiti SH, Sandha GS. Magnetic imaging-assisted colonoscopy vs conventional colonoscopy: a randomized controlled trial. *World J Gastroenterol.* 2014;20(36):13178-13184.
371. Galie N, Barbera JA, Frost AE, et al. Initial Use of Ambrisentan plus Tadalafil in Pulmonary Arterial Hypertension. *N Engl J Med.* 2015;373(9):834-844.
372. Mansour R, Tawab N, Kamal O, et al. Intrauterine injection of human chorionic gonadotropin before embryo transfer significantly improves the implantation and pregnancy rates in in vitro fertilization/intracytoplasmic sperm injection: a prospective randomized study. *Fertil Steril.* 2011;96(6):1370-1374 e1371.
373. Qian G, Xue K, Tang L, et al. Mitigation of oxidative damage by green tea polyphenols and Tai Chi exercise in postmenopausal women with osteopenia. *PLoS One.* 2012;7(10):e48090.
374. Patrick K, Norman GJ, Davila EP, et al. Outcomes of a 12-month technology-based intervention to promote weight loss in adolescents at risk for type 2 diabetes. *J Diabetes Sci Technol.* 2013;7(3):759-770.
375. Constantine NA, Jerman P, Berglas NF, Angulo-Olaiz F, Chou CP, Rohrbach LA. Short-term effects of a rights-based sexuality education curriculum for high-school students: a cluster-randomized trial. *BMC Public Health.* 2015;15:293.
376. Rains TM, Leidy HJ, Sanoshy KD, Lawless AL, Maki KC. A randomized, controlled, crossover trial to assess the acute appetitive and metabolic effects of sausage and egg-based convenience breakfast meals in overweight premenopausal women. *Nutr J.* 2015;14:17.
377. Speich B, Ame SM, Ali SM, et al. Efficacy and safety of nitazoxanide, albendazole, and nitazoxanide-albendazole against Trichuris trichiura infection: a randomized controlled trial. *PLoS Negl Trop Dis.* 2012;6(6):e1685.
378. Dziedzic K, Nicholls E, Hill S, et al. Self-management approaches for osteoarthritis in the hand: a 2x2 factorial randomised trial. *Ann Rheum Dis.* 2015;74(1):108-118.
379. Du Toit G, Roberts G, Sayre PH, et al. Randomized trial of peanut consumption in infants at risk for peanut allergy. *N Engl J Med.* 2015;372(9):803-813.
380. Diamond MP, Carr B, Dmowski WP, et al. Elagolix treatment for endometriosis-associated pain: results from a phase 2, randomized, double-blind, placebo-controlled study. *Reprod Sci.* 2014;21(3):363-371.
381. Ibrahim SA, Hanusa BH, Hannon MJ, Kresevic D, Long J, Kent Kwoh C. Willingness and access to joint replacement among African American patients with knee osteoarthritis: a randomized, controlled intervention. *Arthritis Rheum.* 2013;65(5):1253-1261.
382. Fried MW, Buti M, Dore GJ, et al. Once-daily simeprevir (TMC435) with pegylated interferon and ribavirin in treatment-naive genotype 1 hepatitis C: the randomized PILLAR study. *Hepatology.* 2013;58(6):1918-1929.

383. Dobbins RL, Shearn SP, Byerly RL, et al. GSK256073, a selective agonist of G-protein coupled receptor 109A (GPR109A) reduces serum glucose in subjects with type 2 diabetes mellitus. *Diabetes Obes Metab.* 2013;15(11):1013-1021.
384. Marrazzo JM, Ramjee G, Richardson BA, et al. Tenofovir-based preexposure prophylaxis for HIV infection among African women. *N Engl J Med.* 2015;372(6):509-518.
385. Martinez FJ, Boscia J, Feldman G, et al. Fluticasone furoate/vilanterol (100/25; 200/25 mug) improves lung function in COPD: a randomised trial. *Respir Med.* 2013;107(4):550-559.
386. Chiva-Blanch G, Urpi-Sarda M, Ros E, et al. Effects of red wine polyphenols and alcohol on glucose metabolism and the lipid profile: a randomized clinical trial. *Clin Nutr.* 2013;32(2):200-206.
387. Donnelly JE, Honas JJ, Smith BK, et al. Aerobic exercise alone results in clinically significant weight loss for men and women: midwest exercise trial 2. *Obesity (Silver Spring).* 2013;21(3):E219-228.
388. Bays HE, Averna M, Majul C, et al. Efficacy and safety of ezetimibe added to atorvastatin versus atorvastatin uptitration or switching to rosuvastatin in patients with primary hypercholesterolemia. *Am J Cardiol.* 2013;112(12):1885-1895.
389. Sykes AP, Kemp GL, Dobbins R, et al. Randomized efficacy and safety trial of once-daily remogliflozin etabonate for the treatment of type 2 diabetes. *Diabetes Obes Metab.* 2015;17(1):98-101.
390. de Silva HA, Pathmeswaran A, Ranasinha CD, et al. Low-dose adrenaline, promethazine, and hydrocortisone in the prevention of acute adverse reactions to antivenom following snakebite: a randomised, double-blind, placebo-controlled trial. *PLoS Med.* 2011;8(5):e1000435.
391. Kullgren JT, Dicks TN, Fu X, et al. Financial incentives for completion of fecal occult blood tests among veterans: a 2-stage, pragmatic, cluster, randomized, controlled trial. *Ann Intern Med.* 2014;161(10 Suppl):S35-43.
392. Lodha R, Mukherjee A, Singh V, et al. Effect of micronutrient supplementation on treatment outcomes in children with intrathoracic tuberculosis: a randomized controlled trial. *Am J Clin Nutr.* 2014;100(5):1287-1297.
393. Fuchs-Buder T, Baumann C, De Guis J, Guerci P, Meistelman C. Low-dose neostigmine to antagonise shallow atracurium neuromuscular block during inhalational anaesthesia: A randomised controlled trial. *Eur J Anaesthesiol.* 2013;30(10):594-598.

© 2019 Chen T et al. *JAMA Network Open*.
